# Microarray and Proteomic Analyses of Myeloproliferative Neoplasms with a Highlight on the mTOR Signaling Pathway

**DOI:** 10.1371/journal.pone.0135463

**Published:** 2015-08-14

**Authors:** Vladan P. Čokić, Pascal Mossuz, Jing Han, Nuria Socoro, Bojana B. Beleslin-Čokić, Olivera Mitrović, Tijana Subotički, Miloš Diklić, Danijela Leković, Mirjana Gotić, Raj K. Puri, Constance Tom Noguchi, Alan N. Schechter

**Affiliations:** 1 Institute for Medical Research, University of Belgrade, Belgrade, Serbia; 2 Département d'Hématologie, Institut de Biologie et Pathologie, CHU Grenoble, Grenoble, France; 3 Tumor vaccines and Biotechnology Branch, Division of Cellular and Gene Therapies, Center for Biologics Evaluation and Research, Food and Drug Administration, Bethesda, MD, United States of America; 4 Laboratoire TIMC IMAG, Faculté de Médecine de Grenoble, La Tronche Cedex, Grenoble, France; 5 Clinic of Endocrinology, Diabetes and Diseases of Metabolism, Clinical Center of Serbia, Belgrade, Serbia; 6 Clinic of Hematology, Clinical Center of Serbia, Belgrade, Serbia; 7 Medical Faculty, University of Belgrade, Belgrade, Serbia; 8 Molecular Medicine Branch, National Institute of Diabetes and Digestive and Kidney Diseases, National Institutes of Health, Bethesda, MD, United States of America; French Blood Institute, FRANCE

## Abstract

The gene and protein expression profiles in myeloproliferative neoplasms (MPNs) may reveal gene and protein markers of a potential clinical relevance in diagnosis, treatment and prediction of response to therapy. Using cDNA microarray analysis of 25,100 unique genes, we studied the gene expression profile of CD34^+^ cells and granulocytes obtained from peripheral blood of subjects with essential thrombocythemia (ET), polycythemia vera (PV) and primary myelofibrosis (PMF). The microarray analyses of the CD34^+^ cells and granulocytes were performed from 20 de novo MPN subjects: JAK2 positive ET, PV, PMF subjects, and JAK2 negative ET/PMF subjects. The granulocytes for proteomic studies were pooled in 4 groups: PV with JAK2 mutant allele burden above 80%, ET with JAK2 mutation, PMF with JAK2 mutation and ET/PMF with no JAK2 mutation. The number of differentially regulated genes was about two fold larger in CD34^+^ cells compared to granulocytes. Thirty-six genes (including *RUNX1*, *TNFRSF19*) were persistently highly expressed, while 42 genes (including *FOXD4*, *PDE4A*) were underexpressed both in CD34^+^ cells and granulocytes. Using proteomic studies, significant up-regulation was observed for MAPK and PI3K/AKT signaling regulators that control myeloid cell apoptosis and proliferation: RAC2, MNDA, S100A8/9, CORO1A, and GNAI2. When the status of the mTOR signaling pathway related genes was analyzed, PI3K/AKT regulators were preferentially up-regulated in CD34^+^ cells of MPNs, with down-regulated major components of the protein complex EIF4F. Molecular profiling of CD34^+^ cells and granulocytes of MPN determined gene expression patterns beyond their recognized function in disease pathogenesis that included dominant up-regulation of PI3K/AKT signaling.

## Introduction

Myeloproliferative neoplasm (MPN) is a group of stem/progenitor cell-derived clonal disorders with defective regulation of myeloid cell proliferation, explained by an overproduction of mature blood cells. The classic BCR/ABL-negative MPNs include polycythemia vera (PV), essential thrombocythaemia (ET) and primary myelofibrosis (PMF). Their molecular pathogenesis has been associated with an acquired gain-of-function mutations, majority in the Janus kinase 2 (JAK2) activating the JAK/STAT pathway [1,2]. *JAK2V617F* mutation was linked to significantly augmented levels of phosphorylated STAT5 and AKT in bone marrow hematopoietic cells [3]. The AKT, downstream of phosphoinositide-3-kinase (PI3K), is a key regulator of cell survival, proliferation and differentiation, and has been found dysregulated in various cancer cells [4]. The main target of activated AKT was the serine/threonine kinase mammalian target of rapamycin (mTOR). JAK inhibition abolished phosphorylation of JAK/STAT and PI3K/mTOR pathway members in acute lymphoblastic leukemia (ALL), suggesting an interconnection between these signaling networks [5]. PI3K/mTOR inhibition induced a positive feedback response that lead to the activation of JAK2/STAT5 (restores pAKT), consequently diminishing the reaction of the PI3K inhibitors [6]. PI3K/AKT/mTOR inhibitors induced cell-cycle growth arrest, apoptosis and blocked colony development from MPN hematopoietic progenitors, whereas co-treatment with JAK2-inhibitors synergistically induced apoptosis of the primary CD34^+^ MPN cells and significantly reduced erythropoietin-independent colony growth in patients with PV [7,8].

Categorizing a specific gene signature may provide new insights into pathogenesis of MPN. Previous studies were focused on various cells to examine gene expression profile in MPN: whole blood [9,10], CD34^+^ cells [11–13], granulocytes [11,14–18], and bone marrow CD34-derived megakaryocytic cells [19]. Some studies described a general gene expression profile in MPNs [9,10], while others were concentrated into PV [12–15,18], ET [16,17,19] or PMF [11,12]. A few studies explored the gene expression profile of JAK2 mutation positive and negative subjects only in ET [9,16,17]. Microarray analyses have been performed on MPN subjects with cytoreductive therapy [9,10,14], de novo MPN subjects [13,16] and combined [12,19]. Serum proteome of ET subjects was not influenced by the presence of *JAK2V617F*, as determined by mass spectrometry-based analysis of serum protein profiles [20]. Moreover, the proteomic screening of granulocyte proteins from MPN subjects, showed differential expression of 60 proteins in PV and ET [21].

Previous microarray and proteomic studies did not represent an overview of all types of MPN in correlation to *JAK2V617F* mutation, and did not explore primitive and mature hematopoietic cells simultaneously. To compare these diversities, we combined extensive gene expression and proteomic profiling in CD34^+^ cells and granulocytes from JAK2 mutation positive MPN (PV, ET and PMF) and JAK2 negative MPN (ET and PMF) subjects. Furthermore, we focused the gene expression profiling to the mTOR signaling pathway in MPN. Since the aberrant activity of the PI3K/AKT/mTOR pathway was commonly observed in cancers, and mTOR inhibitors were used in clinical trials, our current comprehensive results offer an opportunity to discern the molecular mechanism involved in inhibition of the signaling pathway in MPN.

## Materials and Methods

### Isolation of CD34^+^ cells and granulocytes from the peripheral blood of de novo MPN subjects

Written informed consent was obtained from all patients being included in the study. All patients had signed the consent form approved by the ethical committee, Faculty of medicine, University of Belgrade. All study de novo MPN patients were subject to 30 ml of peripheral blood draw on one occasion, collected in 10% sodium citrate. The maximum time interval between venepuncture of patients and samples arrival in the laboratory was 2 hours. Each 30 ml of diluted lymphocytes and other mononuclear cells (1:1,2 ratio of blood with Ca^2+^/Mg^2+^-free PBS) was then layered gently on top of 15 ml lymphocyte separation medium (LSM, PAA Laboratories GmbH, Pasching, Austria). After centrifugation (400g, 30 min, 20°C), the interface containing mononuclear cells was collected and washed with PBS. The CD34^+^ cells have been isolated from the collected mononuclear cells using a magnetic separation column (Super Macs II, Miltenyi Biotec, Bergisch Gladbach, Germany) and a mixture of magnetic microbeads conjugated with an antibody against CD34 (Miltenyi Biotec) according to the manufacturer's instructions. The formed pellet, during centrifugation with LSM, was comprised mostly of erythrocytes and granulocytes that migrated through the gradient. Contaminating erythrocytes were removed by lysing solution (0.15 M NH_4_Cl, 0.1 mM Na_2_EDTA, 12 mM NaHCO_3_). High quality of purified granulocytes was confirmed by cytospin preparations and Wright–Giemsa staining. The viable CD34^+^ cell and granulocyte counts were performed with the use of a trypan-blue exclusion technique (BioWhittaker). The purity of recovered cells was determined by flow cytometry using PE–anti-CD34 mAb (BD Biosciences, San Jose, CA, USA) and was over 80% in samples for microarray analysis. Karyotype analysis did not show any chromosome aberrations in samples for microarray analysis.

### Isolation of total RNA

We used RNeasy protocol for isolation of total RNA from CD34^+^ cells and granulocytes according to the manufacturer's instructions (Qiagen GmbH, Hilden, Germany). Concentration and integrity of total RNA was assessed using NanoDrop spectrophotometer (Thermo Fisher Scientific Inc., Wilmington, Delaware, USA) and Agilent 2100 Bioanalyzer Software (Agilent Technologies, Waldbronn, Germany) comparing the ratio of 28S and 18S RNA peaks to ensure that there was a minimal degradation of the RNA sample.

### DNA sequencing

Genomic DNA was extracted from peripheral blood granulocytes of MPN subjects by using the proteinase K and phenol-chloroform technique. Single nucleotide mutation *JAK2V617F* was characterized by DNA sequencing after PCR amplification. PCR amplification was performed with wild-type JAK-2-specific forward primer 5′- tggcagagagaattttctgaact -3′ and reverse primer 5′- ttcattgctttcctttttcaca-3′, confirmed by electrophoresis on an ethidium bromide–impregnated 1% agarose gel. PCR amplified samples were analyzed by sequencing on an automated ABI PRISM 3130 Genetic Analyzer (Applied Biosystems; Life Technologies, Carlsbad, CA, USA) with AB DNA Sequencing Analysis Software (v5.2) by using the Big Dye Terminator v3.1 Ready Reaction Cycle Sequencing Kit.

### Isolation of proteins from granulocytes of MPNs

For protein isolation, we formed four groups of de novo MPN subjects: PV with *JAK2V617F* mutant allele burden above 80% (6 subjects: per 3 females and males, average age 60), ET with JAK2 mutation (5 subjects: 4 females and 1 male, average age 49), PMF with JAK2 mutation (6 subjects: 4 females and 2 males, average age 66) and ET/PMF with no JAK2 mutation (Mut0, 7 subjects: 4 females and 3 males, average age 47). A cold lysis buffer (50 mM Tris pH 7.5, 150 mM NaCl, 1% NP-40, 2mM EDTA, 50 mM NaF) was prepared in advance with protease inhibitors (1 mM PMSF, 10 mM ε-aminocaproic acid, 2 μg/ml aprotinin, 50 μg/ml leupeptin, 1 μM pepstatin A) and Na-orthovanadat (1 mM). The granulocytes of MPN subjects were washed twice with PBS and resuspended in 10ml cold PBS with 2 mM EDTA and 1 mM Na-orthovanadat (centrifugation 10 min, 1500 rpm). The cell pellet was dried by inverting over paper towel, vortexed and placed on ice. The cold lysis buffer (with protease inhibitors) was added to the cell pellet, vortexed and placed in refrigerator for 30 min incubation. The cell suspensions were removed in prechilled eppendorf tubes, centrifugation 10000g for 15 min at 4°C. The supernatant was than transfered to a cold eppendorf, mixed and aliquoted into prechilled small eppendorf tubes and frozen at -70°C.

### Protein quantification

Quantification of proteins isolated from granulocytes of MPN subjects was performed using 8-plex iTRAQ (Applied Biosystems). First, the same quantity of protein was taken from each patient to obtain four groups of MPN, with a mixture of 250μg. After tryptic digestion overnight, each sample was split in duplicate leading to 8 aliquots. 100μg of each aliquot was labeled according to the manufacturer’s instructions. The individually labeled digests were then combined into a single sample mixture and subjected to off gel separation (off gel 3100 Agilent) and desalted on micro spin-columns C18 (Harvard Apparatus) before mass spectrometry analysis. The peptide mixtures were analysed by nano-LC-MS/MS using an ultimate3000 system (Dionex, Amsterdam, The Netherlands) coupled to an LTQ-Orbitrap Velos mass spectrometer (Thermo Scientific, Bremen, Germany). The LTQ-Orbitrap operated in data-dependent acquisition mode with the Xcalibur software. Survey scan MS spectra were acquired in the Orbitrap on the 300–2000 m/z range with the resolution set to a value of 60,000. The five most intense ions per survey were selected for CID fragmentation, and the resulting fragments were analysed in the linear trap (LTQ). Dynamic exclusion was used within 60 seconds to prevent repetitive selection of the same peptide.

### Database search and data analysis

The Mascot Daemon software (version 2.3.0, Matrix Science, London, UK) was used to perform database search in batch mode with all the sample raw files. The following parameters were set for creation of the peak lists: parent ions in the mass range 400–4500, no grouping of MS/MS scans, and threshold at 1000. A peak list was created for each sample analysed, and individual Mascot searches were performed. Data were examined alongside homo sapiens entries in Uniprot protein database (http://www.uniprot.org/). Protein hits were automatically validated if they satisfy one of the following criteria: identification with at least one top ranking peptide with a Mascot score of more than 39 (p-value < 0.001) or at least two top ranking peptides each with a Mascot score of more than 22 (p-value< 0.05). When several proteins matched exactly the same set of peptides, only one member of the protein group was reported in the final list. To evaluate the false positive rate in these experiments, all the initial database searches were performed using the "decoy" option of Mascot. For quantification of peptide, the intensity of reporter ions (m/z: 113; 114; 115; 116; 118; 119 and 121) was divided by the intensity of the reporter ion (m/z: 117) for each measured compound. All ratios were normalized against the median intensity of reporters. Only proteins quantified with at least two peptides ratio for each condition were validated. Ratio were transformed into natural logarithms and plotted against the number of peptides subjected to MS/MS analysis. Gaussian curves were fitted on the smoothed histograms. Proteins with natural log-transformed ion ratios differing by at least 2×SD (95% confidence) were considered significantly different from the random variation. Student t test was performed for comparison of ratio corresponding to each replicate (i.e. 116/117 *vs*. 121/117). Only proteins without a significant difference between the replicates were maintained for comparison between subgroups of patients.

### Microarray analysis

The human oligo probe set was purchased from Operon Human Genome Array-Ready Oligo Set Version 4.0 (Eurofins MWG Operon, Huntsville, AL, USA) which contained 35,035 oligonucleotide probes, representing approximately 25,100 unique genes. The human version 4.0 was constructed based on the Ensembl human database build (NCBI-35c), with a full coverage on NCBI human Refseq dataset. The arrays were produced in house at the Center for Biologics Evaluation and Research, U.S. Food and Drug Administration by spotting oligonucleotides on poly-L-lysine coated glass slides by Gene Machines robotics (Omnigrid, San Carlos, CA). The MIAME (minimum information about a microarray experiment) guideline was adopted for the data presentation. Total human universal RNA (HuURNA, BD Biosciences, Palo Alto, CA) served as a universal reference control in the competitive hybridization. Also, our prior experience with primary cell cultures includes quantitative PCR with housekeeping genes (S16 and HPRT) to establish similar efficiency of cDNA synthesis and PCR (data not shown). In microarray studies, for determination of gene expression in CD34^+^ cells of:

- PV origin with JAK2 mutation, we used 7 biological and 2 technical replicates (3 females and 4 males, average age 62);
- ET origin with JAK2 mutation, we used 5 biological and one technical replicate (5 females, average age 59);
- PMF origin with JAK2 mutation, we used 3 biological and one technical replicate (3 females, average age 72);
- ET/PMF origin with no JAK2 mutation (Mut0), we used 5 biological replicates (3 females and 2 males, average age 53).

For determination of gene expression in granulocytes, we used per two biological replicates of ET and PMF origin with *JAK2V617F* mutation (4 females, average age 66), three biological replicates of PV origin with *JAK2V617F* mutation (2 females and 1 male, average age 70), and for Mut0 group (ET and PMF origin without *JAK2V617F*) we used per two biological and technical replicates (1 female and 1 male, average age 51). The biological replicates measure a quantity from different donors, while the technical replicates represent RNA samples from one donor hybridized by multiple arrays (independently labeled aliquots from a single RNA sample). CD34^+^ cells yielded lower amounts of total RNA, insufficient for microarray analysis. Therefore, we amplified total RNA using the Amino Allyl MessageAmp II amplified RNA (aRNA) Amplification kit (Life Technologies Corp., Carlsbad, CA, US). This amplification protocol was performed both in CD34^+^ cells and granulocytes of MPN subjects, for parallel studies, according to the manufacturer's instructions and using 300 ng of total RNA for amplification. For microarray analyses we used 3 μg of amplified RNA. Labeled cDNA probes were produced as described [22]. The prepared hybridization mixture of cDNA probe and aRNA was added on the array in slide and placed in MAUI hybridization chamber (BioMicro Systems, Inc., Salt Lake City, UT, USA) and incubated overnight at 42°C. Data Filtration, normalization, and analysis were performed as already described [22]. The microarray data were available from Gene Expression Omnibus (http://www.ncbi.nlm.nih.gov/geo; accession no. GSE55976). Within each group the average correlation was high: PV– 0.91; ET– 0.87; PMF– 0.75; Mut0–0.78 (S1 Table). The correlation was also very high for examined technical replicates of CD34^+^ cells—above 0.92. Also, the average correlation coefficient was high among MPN samples in granulocytes: PV– 0.96; ET– 0.98; PMF– 0.74; Mut0–0.96 (not shown).

### Statistical analysis

For microarray data management and analysis, we used NCI/CIT microArray database (mAdb) system. The one way ANOVA was applied using mAdb software for measurement of statistical significance in gene expression among MPN. For mAdb hierarchical clustering we used uncentered correlation with a modified Pearson correlation equation that assumes the means are 0.

## Results

### Gene expression in CD34^+^ cells and granulocytes of MPN

Reversibly transcribed amplified mRNAs from circulating CD34^+^ cells and granulocytes of de novo MPN subjects (labeled with Cy5 dye) and cDNA derived from HuURNA (labeled with Cy3 dye) were mixed and hybridized with oligonucleotide in microarrays. Results were analyzed for gene expression. The total number of expressed genes in MPNs was presented in Table 1 (first column, after 50% filtering). The total gene expression was about two fold larger in CD34^+^ cells compared to granulocytes. A total of 87 genes were highly overexpressed (four-fold and higher compared to HuURNA) in ET, 84 genes in PV, 68 in PMF with *JAK2V617F* mutation and 42 genes in ET/PMF *JAK2V617F* negative (Mut0)-derived CD34^+^ cells, while in granulocytes even higher quantity of overexpressed genes were observed (Table 1). Out of that, we made a list of 94 and 68 common highly up-regulated and defined genes in CD34^+^ cells and granulocytes of MPNs, respectively (S2 Table). The 36 genes were persistently highly up-regulated both in CD34^+^ cells and granulocytes: runt-related transcription factor 1 (*RUNX1*), tumor necrosis factor receptor superfamily 19 (*TNFRSF19*), STE20-related kinase adaptor beta (*STRADB*), synuclein beta (*SNCB*, S2 Table). In addition, we presented a list of 101 and 60 common highly down-regulated and defined genes in CD34^+^ cells and granulocytes, respectively (S3 Table). Forty-two genes were persistently highly down-regulated both in CD34^+^ cells and granulocytes: forkhead box D4 (*FOXD4*), phosphodiesterase 4A, cAMP-specific (*PDE4A*, S3 Table).

**Table 1:** Total number of genes and quantification of highly up-regulated and down-regulated genes versus HuURNA in MPN with or without filtering.

Filtering		>50%						
Cells	MPN	total	2–3 fold		3–4 fold		≥ 4 fold	
			UR	DR	UR	DR	UR	DR
**CD34** ^**+**^	**ET**	4921	380	252	85	101	87	21
	**PV**	5522	459	233	117	117	84	38
	**PMF**	4277	227	365	48	81	68	5
	**Mut0**	4744	267	198	54	79	42	68
**Granulocytes**	**ET**	2059	297	352	145	20	149	0
	**PV**	2050	354	398	164	14	131	0
	**PMF**	2485	233	365	108	81	52	5
	**Mut0**	1538	215	305	98	36	118	1

UR—up-regulated and DR—down-regulated genes

**Fig 1:**
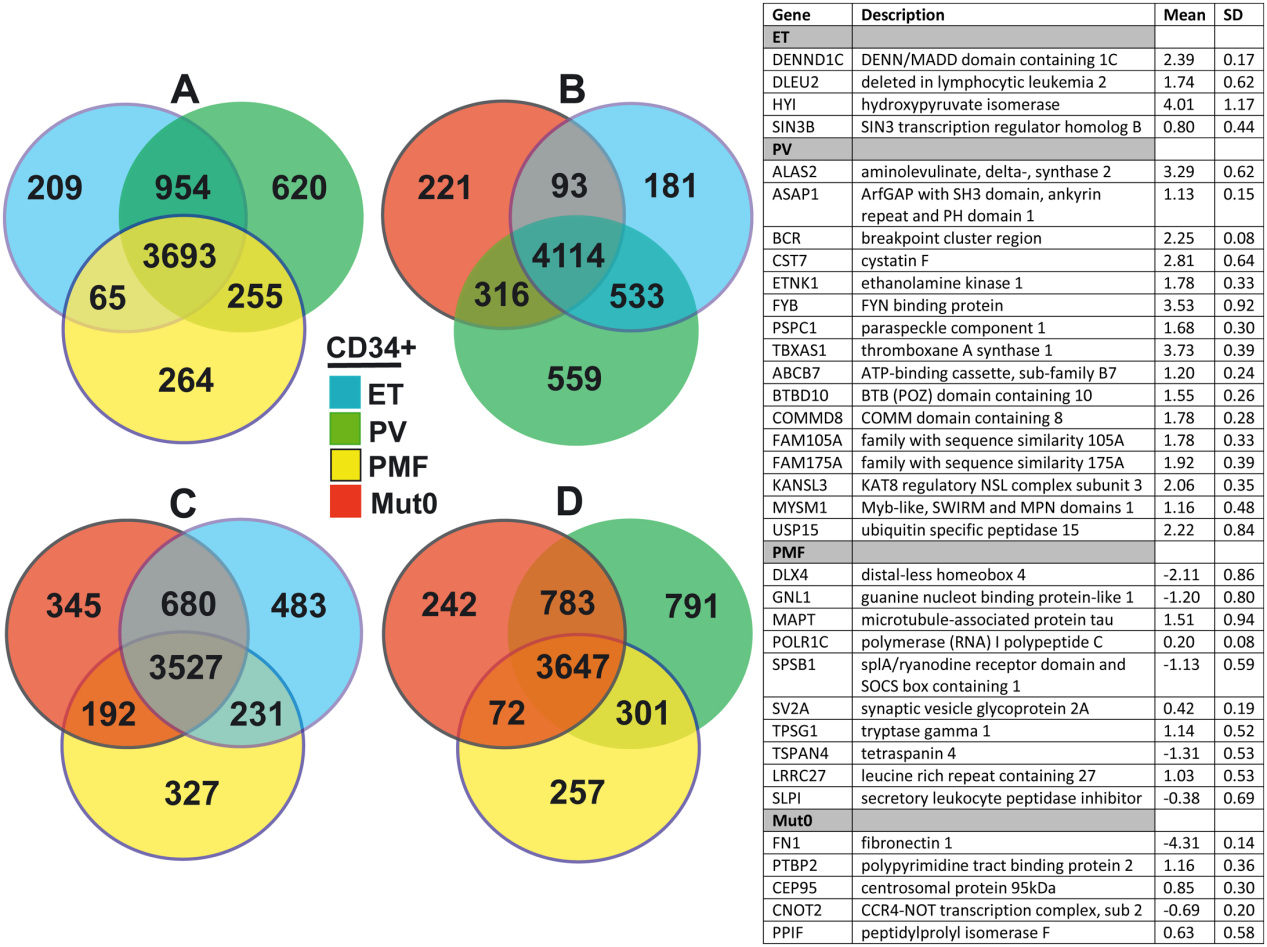
Comparison of total gene expression in CD34^+^ cells among MPNs by Venn diagram after 50% filtration. A) Among *JAK2V617F*+ ET, PV and PMF; Among *JAK2V617F* negative ET/PMF (Mut0) and B) *JAK2V617F*+ ET and PV; C) *JAK2V617F*+ ET and PMF; D) *JAK2V617F*+ PV and PMF. Table represents the genes exclusively expressed in a single MPN disorder.

**Fig 2:**
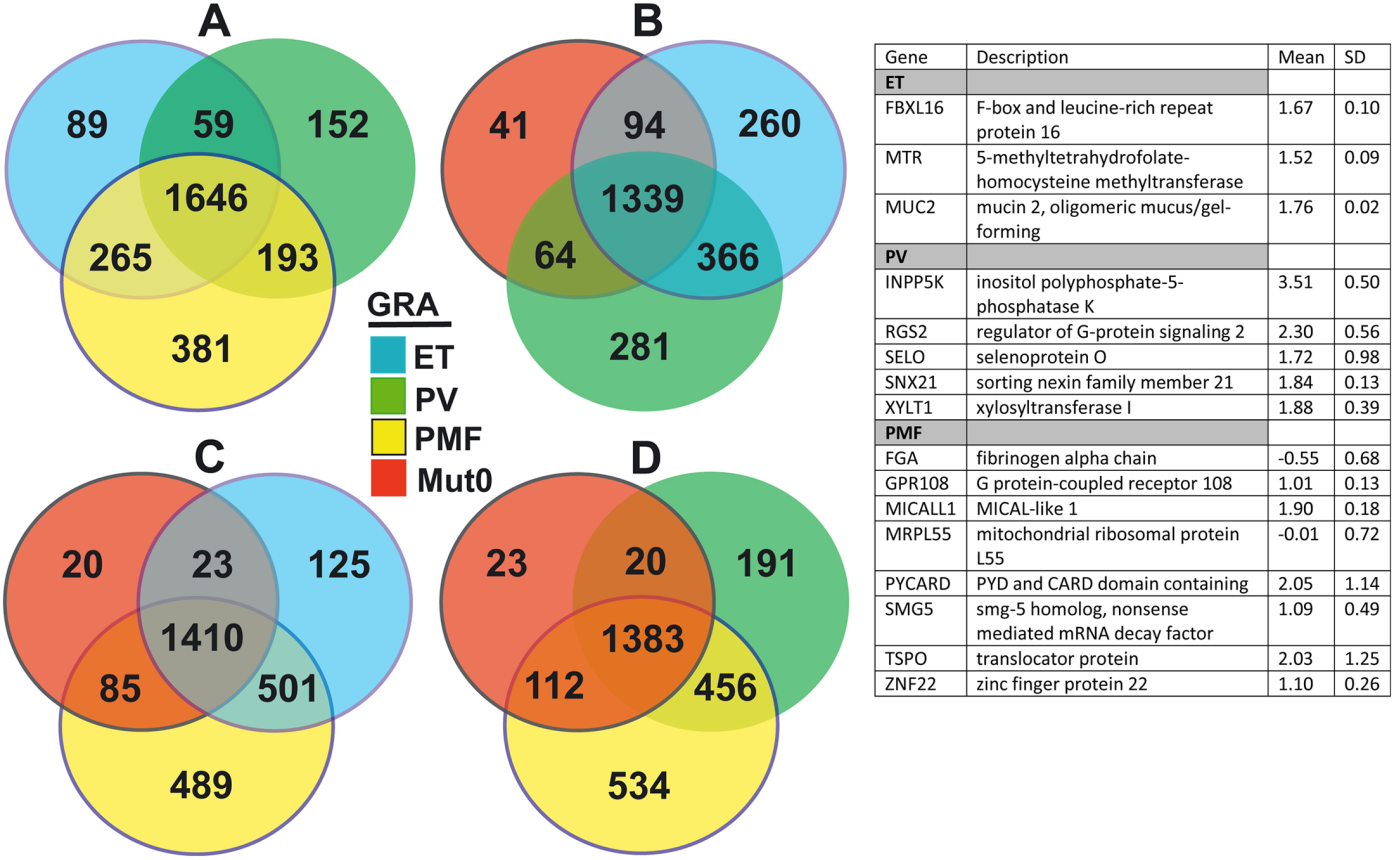
Comparison of total gene expression in granulocytes among MPNs by Venn diagram after 50% filtration. A) Among *JAK2V617F*+ ET, PV and PMF; Among *JAK2V617F* negative ET/PMF (Mut0) and B) *JAK2V617F*+ ET and PV; C) *JAK2V617F*+ ET and PMF; D) *JAK2V617F*+ PV and PMF. Table represents the genes exclusively expressed in a single MPN disorder.

Distribution and overlapping of gene expression in CD34^+^ cells and granulocytes, of MPNs, have been demonstrated by Venn diagram (Figs 1 and 2). According to Venn diagram, the genes exclusively overexpressed (not even sporadic after 50% filtration) in CD34^+^ cells of PV (*ALAS2*, *FYB*, *TBXAS1*) and ET (*HYI*, *DENND1C*) origin were presented in table of Fig 1. The genes only present and up-regulated in CD34^+^ cells of *JAK2V617F* positive PMF origin were: *TPSG1*, *LRRC27*, *MAPT*, while down-regulated were: *SPSB1*, *DLX4*, *TSPAN4*, and *GNL1*. The genes solely up-regulated in CD34^+^ cells of *JAK2V617F* negative ET/PMF origin were *CEP95*, *PTBP2*, while down-regulated were *CNOT2* and *FN1* (Fig 1). In addition, the genes exclusively overexpressed in granulocytes of ET (*MUC2*), PV (*INPP5K*) and PMF (*PYCARD*) origin were presented in table of Fig 2.

### Expression of genes that regulate myeloid cell proliferation, differentiation and apoptosis

In accordance with highly up-regulated and down-regulated genes (S2 and S3 Tables), we analyzed genes that regulate myeloid cell proliferation, differentiation and apoptosis in CD34^+^ cells and granulocytes of MPNs. Expression of myeloid differentiation stimulating genes were up-regulated for: *RUNX1*, v-yes-1 Yamaguchi sarcoma viral related oncogene homolog (*LYN*), hematopoietic cell-specific Lyn substrate 1 (*HCLS1*); as well as myeloid differentiation inhibiting genes such as Kruppel-like factor 13 (*KLF13*) and nuclear factor of kappa light polypeptide gene enhancer in B-cells inhibitor, alpha (*NFKBIA*) in MPNs (Table 2). Among up-regulated genes that stimulates cell proliferation were MAS1 oncogene (*MAS1*), ras-related C3 botulinum toxin substrate 2 (*RAC2*) and signal transducer and activator of transcription 1 (*STAT1*); as well as those that inhibits cell proliferation: retinoic acid receptor responder 3 (*RARRES3*), and transducer of ERBB2 1 (*TOB1*, Table 2). Expression of apoptosis-stimulating genes S100 calcium binding protein A8 (*S100A8*) and *TNFRSF19* was up-regulated, as well as the expression of apoptosis-inhibiting genes NLR family apoptosis inhibitory protein (*NAIP*, absent in PMF) and tumor necrosis factor, alpha-induced protein 3 (*TNFAIP3*). Inhibition of apoptosis can be achieved via the PI3K/AKT pathway, stimulated by up-regulated coiled-coil domain containing 88A (*CCDC88A*) and coronin 1A (*CORO1A*) genes, while inhibited by up-regulated *KLF13* gene in CD34^+^ cells and granulocytes of MPNs (Table 2). The MAPK pathway played a critical role in regulation of cell proliferation [23]. MAPK pathway was stimulated by up-regulated chemokine (C-X-C motif) receptor 4 (*CXCR4*), growth arrest and DNA-damage-inducible, beta (*GADD45B*) and *MAPK10* genes expression. In addition, MAPK pathway was inhibited by up-regulated dual specificity phosphatase 1 (*DUSP1*) and protein tyrosine phosphatase, receptor type J (*PTPRJ*) genes (Table 2).

**Table 2:** Cell differentiation, proliferation and apoptosis related genes expression in CD34^+^ cells and granulocytes of MPN subjects.

			PV		ET		PMF		Mut0	
	Genes	Cells	Mean	SD	Mean	SD	Mean	SD	Mean	SD
1	**Myeloid cell differentiation**									
A	HCLS1 ^4A^	CD34	4.36	*0*.*35*	4.28	*0*.*76*	3.77	*0*.*10*	3.70	*0*.*81*
A	JAK2 ^3A, 4A^	CD34	4.02	*0*.*45*	3.54	*0*.*81*	3.01	*0*		
A	LYN ^2A, 4A, 5B^	CD34	3.81	*0*.*44*	3.35	*0*.*95*	3.41	*0*	3.69	*0*.*74*
A	PTPRC	CD34	3.86	*0*.*32*	4.55	*0*	3.68	*0*	4.34	*0*
		**GRA**	**-1.27**	***0*.*45***	**-1.08**	***0***	**-2.32**	***0***	**-0.27**	***0***
A	RUNX1	CD34	4.54	*0*.*45*	4.85	*0*.*50*	5.34	*0*.*25*	4.47	*0*.*35*
		**GRA**	**5.81**	***1*.*05***	**5.71**	***0***	**5.18**	***0*.*15***	**5.74**	***0***
A	SPI1	CD34	3.78	*0*.*55*	2.92	*0*.*73*			3.09	*1*.*34*
B	KLF13 ^2B^	CD34	3.74	*0*.*45*	3.15	*0*.*55*	2.69	*0*.*17*	3.4	*0*.*73*
B	NFKBIA	CD34	3.62	*0*.*73*	4.21	*1*.*04*	3.34	*0*.*51*	3.30	*1*.*10*
B	NME1 ^2B^	CD34	-2.16	*0*.*5*	-2.39	*0*.*5*	-1.27	*0*.*5*	-2.04	*0*.*57*
		**GRA**	**-1.42**	***0*.*33***	**-1.14**	***0*.*14***	**-1.80**	***0*.*32***	**-1.41**	***0*.*27***
2	**Cell proliferation**									
A	CYBA	CD34	2.25	*0*.*80*	2.68	*0*.*51*	1.90	*0*.*16*	2.45	*0*.*41*
A	EIF5A	CD34	-2.60	*0*.*20*	-2.77	*0*	-2.13	*0*.*12*	-1.81	*0*.*06*
A	MAS1	CD34	4.12	*0*.*92*	5.00	*0*.*77*	5.49	*0*.*14*	4.95	*0*.*19*
A	PGF	CD34	-3.47	*0*.*49*	-2.62	*0*.*55*	-3.64	*0*.*95*	-2.94	*0*.*78*
		**GRA**	**-1.39**	***0*.*16***	**-1.59**	***0*.*10***	**-2.22**	***1*.*25***	**-0.84**	***0*.*52***
A	PLAC8 ^3B^	CD34	2.97	*0*.*68*	3.33	*0*.*56*			4.20	*0*
A	PRAME ^1B, 3B^	CD34	-3.39	*0*.*32*	-3.04	*0*.*46*	-3.01	*0*.*21*	-2.97	*0*.*42*
		**GRA**	**-1.42**	***0*.*01***	**-1.17**	***0*.*03***	**-2.65**	***0*.*75***	**-1.48**	***0*.*35***
A	PTEN ^3B, 4B^	CD34	3.08	*0*.*26*	2.86	*0*.*56*	3.01	*0*.*37*	2.53	*0*.*78*
A	PTPRC	CD34	3.86	*0*.*32*	4.55	*0*	3.68	*0*	4.34	*0*
		**GRA**	**-1.27**	***0*.*45***	**-1.08**	***0***	**-2.32**	***0***	**-0.27**	***0***
A	RAC2	CD34	3.43	*0*.*29*	3.22	*0*.*49*	2.97	*0*.*32*	3.36	*0*.*46*
		**GRA**			**0.92**	***0***	**3.18**	***1*.*62***	**1.40**	***0*.*21***
A	SAT1	CD34	3.63	*0*.*63*	4.12	*0*.*99*	1.93	*1*.*17*	3.01	*0*.*76*
A	STAT1	CD34	4.76	*0*.*50*	4.68	*0*.*85*	4.18	*0*.*39*	3.35	*1*.*10*
B	IL8	CD34	3.73	*1*.*6*	3.75	*2*.*11*	2.11	*2*.*85*	3.84	*1*.*91*
		**GRA**	**2.76**	***0***			**6.16**	***0***	**1.52**	***0*.*63***
B	KLF11 ^3A^	CD34	2.50	*0*.*14*	2.74	*1*.*07*	4.09	*0*	2.14	*0*
B	RARRES3	CD34	3.79	*0*.*57*	3.47	*0*.*55*	3.44	*0*.*20*	3.04	*0*.*12*
B	RBM5	CD34	3.04	*0*.*18*	2.67	*0*.*44*	1.94	*0*	2.4	*0*.*48*
B	SERPINE2 ^4A^	CD34	-3.25	*0*.*74*	-3.15	*0*.*55*	-3.02	*0*.*54*	-2.87	*0*.*62*
		**GRA**	**-1.51**	***0*.*12***	**-2.00**	***0*.*16***	**-3.02**	***0*.*54***	**-1.66**	***0*.*52***
B	SKAP2	CD34	2.86	*0*.*44*	3.16	*0*.*74*	2.73	*0*.*33*	2.59	*0*.*18*
B	SOD2	CD34	3.02	*0*.*71*	3.32	*0*.*52*	3.40	*0*.*93*	3.50	*0*.*02*
		**GRA**	**0.24**	***0*.*40***	**-0.15**	***0***	**1.7**	***2*.*06***	**0.23**	***0*.*27***
B	TOB1	CD34	2.33	*1*.*09*	2.72	*1*.*30*	4.15	*0*.*23*	2.87	*0*.*63*
		**GRA**	**3.93**	***1*.*61***	**2.38**	***0*.*11***	**1.26**	***0*.*58***	**2.34**	***0*.*86***
3	**Apoptotic process**									
A	MNDA	CD34	4.23	*0*.*36*	3.14	*0*.*61*				
A	S100A8	CD34	6.07	*0*.*46*	5.67	*1*.*16*	6.36	*0*.*17*	5.58	*0*.*26*
A	S100A9	CD34	4.98	*0*.*40*	5.14	*0*.*60*	4.43	*0*.*62*	4.95	*0*.*65*
		**GRA**	**5.10**	***0*.*20***	**2.04**	***0*.*33***	**5.64**	***1*.*53***	**2.52**	***1*.*06***
A	TNFRSF19	CD34	2.27	*1*.*08*	2.98	*1*.*10*	4.11	*0*.*61*	2.34	*1*.*34*
		**GRA**	**4.36**	***1*.*04***	**4.33**	***0*.*35***	**3.59**	***0*.*80***	**4.55**	***0*.*43***
A	TXNIP	CD34	2.68	*0*.*70*	2.91	*0*.*27*	2.34	*0*	2.31	*1*.*16*
B	NAIP	CD34	2.78	*0*.*62*	2.81	*0*.*31*			2.37	0.2
B	TNFAIP3	CD34	2.57	*0*.*83*	2.71	*1*.*13*	1.00	*0*.*53*	3.18	*0*.*76*
4	PI3K / AKT activity									
A	CCDC88A	CD34	4.06	*0*.*64*	4.06	*0*.*83*	5.32	*0*	3.35	*1*.*13*
		**GRA**	**5.02**	***0*.*73***	**4.87**	***0*.*05***	**5.16**	***0*.*11***	**5.08**	***0*.*44***
A	CORO1A	CD34	4.15	*0*.*44*	4.02	*0*.*52*	3.57	*0*.*14*	4.01	*0*.*95*
		**GRA**	**1.28**	***0*.*35***	**0.5**	***0***	**3.22**	***2*.*52***	**0.40**	***0***
A	SEMA4D ^3B^	CD34	2.76	*0*.*56*	2.8	*0*.*94*	2.29	*0*.*02*	2.11	*0*.*85*
B	KLF13 ^2B^	CD34	3.74	*0*.*45*	3.15	*0*.*55*	2.69	*0*.*17*	3.4	*0*.*73*
5	**MAP kinase activity**									
A	CCL3 ^4A^	CD34	1.81	*0*.*29*	3.06	*0*.*77*	1.46	*0*.*75*	2.37	*0*.*78*
		**GRA**	**2.65**	***0*.*45***	**2.64**	***0*.*07***	**2.37**	***0*.*60***	**3.02**	***0*.*78***
A	CXCR4	CD34	3.80	*0*.*84*	3.37	*1*.*14*	3.71	*0*	3.45	*1*.*08*
A	FPR1	CD34	4.86	*0*.*48*	4.59	*1*.*30*				
A	GADD45B ^1A^	CD34	2.09	*0*.*64*	2.97	*0*.*62*	1.03	*0*.*98*	2.1	*0*.*85*
		**GRA**	**0.46**	***0***	**0.25**	***0***	**2.45**	***1*.*47***	**0.75**	***0***
A	GNAI2 ^2A^	CD34	2.92	*0*.*32*	2.89	*0*.*60*	2.21	*0*.*34*	2.79	*0*.*87*
		**GRA**	**-0.02**	***0*.*45***			**1.26**	***2*.*14***	**-0.87**	***0***
A	IGF2 ^2A, 4A^	CD34	-3.52	*0*.*57*	-3.13	*0*.*45*	2.08	*1*.*33*	-2.93	*0*.*31*
		**GRA**	**-1.38**	***0*.*31***	**-1.51**	***0*.*45***	**-2.63**	***0*.*28***	**1.75**	***0*.*52***
A	MAPK10	CD34	2.69	*0*.*56*	2.67	*0*.*52*	2.82	*1*.*08*	2.45	*1*.*22*
		**GRA**	**4.55**	***0*.*33***	**4.72**	***0*.*11***	**4.03**	***0*.*56***	**4.62**	***0*.*03***
A	PYCARD ^3A^	CD34	2.52	*0*.*35*	2.94	*0*.*54*	2.32	*0*.*073*	2.30	*0*.*33*
A	SSTR4 ^2B^	CD34	-3.86	*0*.*39*	-4.14	*0*.*52*	-4.13	*0*.*81*	-3.92	*0*
		**GRA**	**-2.85**	***0***	**-2.4**	***0*.*11***	**-4.18**	***0***	**-1.94**	***0***
A	VEGFA ^2A, 3B^	CD34	-2.83	*0*.*55*	-2.74	*0*.*59*	-2.76	*1*.*25*	-2.77	*0*.*81*
B	DUSP1 ^3A^	CD34	4.19	*1*.*05*	4.35	*0*.*93*	4.85	*0*	3.66	*0*.*89*
B	DUSP2	CD34	2.90	*0*.*57*	3.00	*1*.*30*	2.29	*0*	3.58	*0*
B	DUSP6 ^1A, 3A^	CD34	3.08	*0*.*58*	3.33	*0*.*75*	2.38	*0*.*69*	3.32	*0*.*74*
B	PTPRJ ^4B^	CD34	3.17	*0*.*64*	3.33	*1*.*14*	2.18	*0*	3.62	*0*.*31*

Superscripts next to genes correspond also to presented groups (1–5); stimulating (A) and inhibiting (B) factors; GRA—granulocytes.

### Statistical analysis of gene expression in MPNs determined by microarray analysis

**Fig 3:**
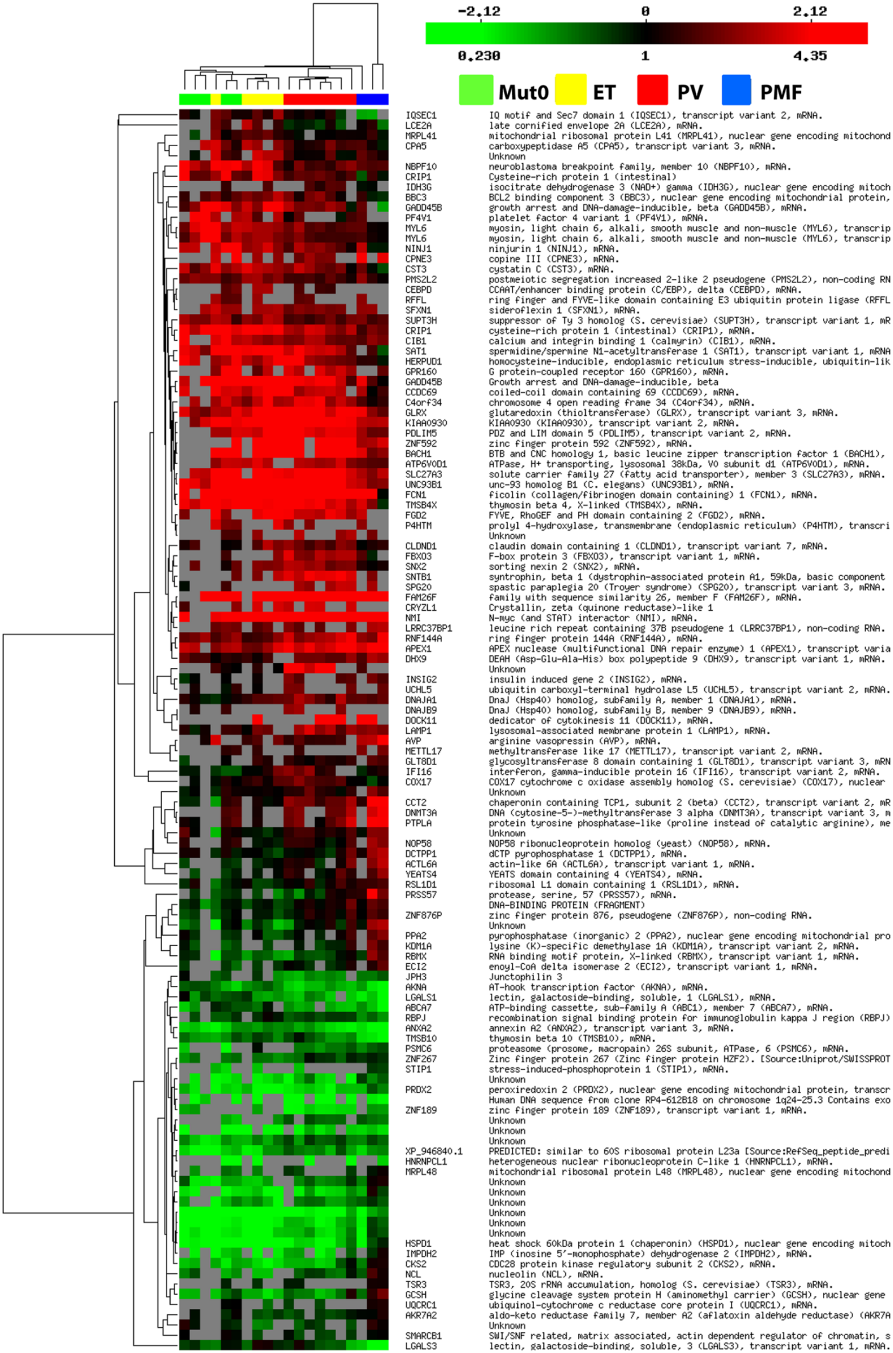
Hierarchical clustering of highly significant genes expressed in CD34^+^ cells of MPNs. Statistical significance (p<0.01) was determined among *JAK2V617F* positive ET (yellow), PV (red), PMF (blue) and *JAK2V617F* negative ET/PMF (Mut0, green) by one way ANOVA. The numbers on top and below the color bar represent intensity of contrasts with offsets at 0 and 1. The total gene expression of MPNs is also clustered (above image) representing similarities among examined cells. The gene and array correlations were uncentered. The gene description is provided in Table 3.

We examined the gene expression in CD34^+^ cells and granulocytes of *JAK2V617F* positive and negative MPN subjects. We determined significantly different genes among MPNs in CD34^+^ cells (p<0.01, Table 3). In addition, these significant genes were also shown in hierarchical clustering analysis to illustrate their associations (Fig 3). A significant gene overexpression was observed for *CRIP1* and *GADD45B* in JAK2 positive ET subjects, while *GLRX*, *IFI16* and *LAMP1* were over-expressed in PV subjects. In JAK2 positive PMF subjects a significant gene overexpression was observed for *CPNE3*, *APEX1* and *KDM1A*, while in JAK2 negative MPN subjects were over-expressed *CIB1*, *NBPF10* and *GPR160* (Table 3, Fig 3). Also, the array clustering of significant genes was in accordance with examined MPN groups, except one *JAK2V617F* positive ET sample mixed with Mut0 group that contained *JAK2V617F* negative ET patients (Fig 3). In granulocytes of JAK2 positive MPN subjects a significant gene over-expression was determined for phosphoribosyl pyrophosphate synthetase-associated protein (*PRPSAP2*), myosin VIIB retinoblastoma-like 2 (*RBL2*), *S100A9*, and microRNA 424 genes (*MIR424*, S4 Table).

**Table 3:** The statistically significant genes (p<0.01) among MPNs in CD34^+^ cells determined by microarray analysis.

			Group A—ET		Group B—PV		Group C—PMF		Group D -Mut0	
Genes	MD	BG	Mean	SD	Mean	SD	Mean	SD	Mean	SD
ABCA7	1.16	A-C	-0.54	0.33	-1.47	0.17	-1.67	0.39	-1.04	0.48
ACTL6A	1.86	C-D	-0.21	0.15	0.28	0.46	1.38	0.24	-0.48	0.09
AKNA	0.89	A-C	-1.04	0.16	-1.59	0.19	-1.93	0.48	-1.09	0.49
AKR7A2	0.69	C-A	-0.63	0.14	-0.53	0.18	0.06	0.15	-0.15	0.19
ANXA2	1.22	A-C	-1.36	0.38	-1.60	0.35	-2.59	0.57	-1.66	0.25
APEX1	1.32	C-A	1.21	0.38	2.23	0.50	2.54	0.63	1.56	0.08
ATP6V0D1	0.91	D-C	1.55	0.25	1.43	0.22	0.75	0.08	1.67	0.02
BACH1	1.86	D-C	2.79	0.43	2.72	0.51	1.36	0.41	3.22	0.06
BBC3	1.18	A-C	1.13	0.26	0.71	0.42	-0.05	0.37	0.57	0.18
CCDC69	1.51	A-C	2.16	0.44	1.72	0.36	0.65	0.61	1.85	0.04
CCT2	1.93	C-A	-0.29	0.61	0.96	0.29	1.63	0.90	0.06	0.39
CEBPD	0.86	A-B	1.43	0.02	0.57	0.09			0.85	0.12
CIB1	1.28	D-C	1.46	0.26	1.09	0.27	0.95	0.23	2.23	0.61
CKS2	1.56	C-A	-1.79	0.30	-1.26	0.59	-0.23	0.60	-1.64	0.23
CLDND1	0.88	A-C	1.09	0.36	0.91	0.26	0.21	0.18	0.26	0.22
COX17	0.88	B-C	0.31	0.31	0.66	0.32	-0.22	0.29	0.08	0.17
CPA5	1.28	A-C	1.47	0.26	0.52	0.28	0.20	0.38	0.94	0.57
CPNE3	1.13	C-B	1.42	0.23	1.08	0.17	2.21	0.26	2.26	0.00
CRIP1	1.54	A-C	2.34	0.23	1.46	0.26	0.81	0.22	2.24	0.52
CRIP1	1.57	D-C	1.42	0.53	0.68	0.18	-0.13	0.52	1.44	0.81
CRYZL1	0.93	A-D	1.82	0.01	1.75	0.08			0.89	0.30
CST3	1.59	A-C	1.65	0.28	0.98	0.46	0.06	0.62	1.24	0.34
DCTPP1	1.30	C-A	-0.29	0.26	0.06	0.42	1.01	0.59	0.09	0.20
DHX9	0.82	C-A	0.31	0.23	0.94	0.17	1.14	0.35	0.55	0.21
DNAJA1	0.92	B-D	0.45	0.46	0.99	0.41	0.77	0.08	0.07	0.23
DNAJB9	0.85	B-D			1.23	0.10			0.39	0.11
DNMT3A	1.25	C-D	0.19	0.00	1.06	0.25	1.93	0.16	0.68	0.05
DOCK11	0.63	B-C	3.25	0.00	3.28	0.06	2.64	0.10		
ECI2	1.17	C-D	-0.48	0.04	-0.15	0.26	0.56	0.26	-0.62	0.21
FAM26F	1.99	B-D	3.43	0.68	4.53	0.46	3.22	0.61	2.54	0.38
FBXO3	1.20	B-C	1.17	0.00	1.41	0.29	0.21	0.50	0.53	0.00
FCN1	2.83	A-C	4.66	0.29	4.00	0.28	1.83	1.38	3.84	0.80
FGD2	1.11	A-D	2.23	0.17	1.74	0.27	1.68	0.00	1.12	0.08
GADD45B	2.20	A-C	3.12	0.55	1.98	0.68	0.92	0.82	2.45	0.53
GCSH	1.74	C-A	-1.61	0.19	-0.95	0.58	0.13	0.80	-1.14	0.42
GLRX	1.19	B-C	2.21	0.56	2.52	0.42	1.33	0.54	1.47	0.43
GLT8D1	0.97	C-D	0.38	0.12	0.29	0.14	0.70	0.08	-0.27	0.29
GPR160	1.11	D-A	1.16	0.34	1.63	0.23	0.82	0.00	2.27	0.00
HERPUD1	1.73	D-C	1.57	0.36	1.46	0.68	0.19	0.27	1.92	0.30
HNRNPCL1	0.45	B-D	-2.62	0.00	-2.27	0.04	-1.76	0.00	-2.72	0.00
HSPD1	1.60	C-A	-2.64	0.09	-1.95	0.59	-1.04	0.80	-2.53	0.39
IDH3G	0.72	B-C	0.68	0.00	0.62	0.17	-0.10	0.03	0.44	0.00
IFI16	1.29	B-D	0.16	0.48	0.81	0.40	0.03	0.28	-0.47	0.27
IMPDH2	2.08	C-A	-2.20	0.16	-1.52	0.47	-0.12	0.32	-1.74	0.22
INSIG2	0.98	B-D	0.12	0.00	0.82	0.18	0.81	0.16	-0.17	0.19
IQSEC1	2.14	A-C	0.72	0.45	0.09	0.29	-1.42	0.03	0.31	0.31
JPH3	0.96	D-B	-0.84	0.32	-1.29	0.17	-0.92	0.13	-0.33	0.26
KDM1A	1.39	C-D	-0.60	0.00	-0.18	0.35	0.80	0.26	-0.59	0.27
KIAA0930	1.29	A-C	2.84	0.55	2.75	0.52	1.56	0.04	1.81	0.47
LAMP1	1.10	B-D	0.62	0.47	1.33	0.37	1.12	0.08	0.23	0.30
LCE2A	1.28	A-B	0.95	0.43	-0.33	0.30	-0.17	0.58	0.53	0.00
LGALS1	1.55	D-C	-1.07	0.37	-1.47	0.26	-2.17	0.48	-0.62	0.43
LGALS3	2.20	A-C	-0.37	0.54	-0.62	0.49	-2.57	0.69	-0.42	0.30
LRRC37BP1	0.67	C-A	0.84	0.10	0.93	0.17	1.50	0.05	1.00	0.04
METTL17	0.61	C-D	0.44	0.00	0.49	0.00	1.04	0.03	0.43	0.05
MRPL41	0.78	A-D	0.76	0.24	0.06	0.09	0.14	0.27	-0.02	0.45
MRPL48	1.02	C-D	-1.08	0.00	-0.51	0.09	-0.02	0.14	-1.05	0.15
MYL6	1.14	A-C	1.43	0.40	0.68	0.21	0.29	0.24	1.41	0.67
MYL6	1.46	D-C	1.70	0.56	0.82	0.27	0.34	0.34	1.79	0.80
NBPF10	1.56	D-B	2.00	0.99	0.68	0.35	0.70	0.06	2.25	0.86
NCL	1.67	C-A	-1.65	0.01	-0.65	0.31	0.02	0.57	-1.11	0.32
NINJ1	1.87	D-C	1.45	0.26	0.89	0.42	0.40	0.86	2.27	0.69
NMI	0.98	B-D	2.75	0.49	3.38	0.27	3.19	0.17	2.40	0.18
NOP58	1.43	C-A	-0.19	0.07	0.25	0.32	1.24	0.14	0.38	0.08
P4HTM	0.89	A-D	1.90	0.06	1.15	0.07	1.20	0.00	1.01	0.03
PDLIM5	1.08	B-C	1.96	0.43	2.10	0.29	1.03	0.23	1.64	0.24
PF4V1	1.66	D-B	1.32	0.37	0.31	0.38	0.77	0.00	1.97	0.39
PMS2L2	0.64	A-B	1.17	0.18	0.52	0.19	0.57	0.47	0.86	0.24
PPA2	1.51	C-A	-0.55	0.41	0.00	0.36	0.96	0.18	-0.29	0.22
PRDX2	1.08	C-A	-2.21	0.36	-1.81	0.19	-1.13	0.37	-1.77	0.18
PRSS57	2.27	C-A	-0.87	0.25	-0.28	0.54	1.41	1.08	-0.41	0.06
PSMC6	1.04	C-D	-0.09	0.00	-1.01	0.17	-0.86	0.23	-1.90	0.23
PTPLA	1.76	C-D	0.22	0.00	0.95	0.55	2.15	0.03	0.38	0.34
RBMX	1.81	C-A	-1.33	0.58	-0.59	0.47	0.48	0.73	-0.47	0.14
RBPJ	0.78	A-C	-0.31	0.31	-0.56	0.13	-1.10	0.19	-0.59	0.16
RFFL	0.61	A-D	1.35	0.05	0.80	0.04	1.19	0.00	0.74	0.01
RNF144A	0.89	B-A	0.67	0.27	1.56	0.35	1.44	0.47	1.08	0.21
RSL1D1	0.99	C-A	-0.47	0.37	0.16	0.30	0.52	0.09	-0.15	0.26
SAT1	2.33	A-C	2.27	0.22	1.77	0.65	-0.06	0.64	2.01	0.66
SFXN1	0.48	A-B	1.50	0.15	1.02	0.10	1.23	0.01	1.32	0.08
SLC27A3	1.54	A-C	2.91	0.58	2.34	0.30	1.37	0.22	2.08	0.47
SMARCB1	0.50	D-B	-0.67	0.00	-0.49	0.20	-0.01	0.04	0.01	0.07
SNTB1	0.62	B-A	1.31	0.26	1.93	0.17	1.04	0.00	0.90	0.00
SNX2	1.05	B-C	1.10	0.05	1.24	0.24	0.19	0.11	0.63	0.32
SPG20	1.26	B-D			1.48	0.18	1.40	0.00	0.22	0.34
STIP1	1.08	C-A	-1.59	0.06	-1.65	0.00	-0.51	0.05	-1.85	0.00
SUPT3H	0.64	A-B	1.34	0.22	0.70	0.20	0.78	0.22	1.07	0.35
TMSB10	1.10	B-C	-0.97	0.35	-0.88	0.41	-1.99	0.12	-1.07	0.28
TMSB4X	1.62	A-C	2.47	0.34	2.07	0.46	0.86	0.93	2.14	0.31
TSR3	0.38	C-B	-0.42	0.00	-0.22	0.01	0.16	0.01	-0.27	0.00
UCHL5	0.84	B-D	0.04	0.00	0.99	0.05	0.86	0.00	0.15	0.24
UNC93B1	1.84	A-C	3.11	0.47	2.67	0.51	1.27	0.24	2.63	0.73
UQCRC1	0.78	C-B	-0.93	0.00	-0.55	0.04	0.23	0.04	-0.02	0.02
YEATS4	1.44	C-D	-0.14	0.08	0.28	0.34	0.97	0.33	-0.47	0.17
ZNF189	0.66	C-B	-1.38	0.00	-1.95	0.18	-1.29	0.02	-1.36	0.05
ZNF267	0.69	C-A	-1.06	0.14	-0.56	0.33	-0.36	0.09	-1.03	0.15
ZNF592	1.15	A-C	2.68	0.40	2.47	0.25	1.54	0.21	2.94	0.00
ZNF876P	0.90	C-A	-0.50	0.29	0.14	0.32	0.39	0.29	-0.38	0.24

The negative values represent down-regulated genes, while positive values represent up-regulated genes compared to HuURNA. BG—Between groups significance with p<0.01, MD—Maximal mean difference of corresponding BG.

### Proteomic analysis of granulocytes in MPNs

It has been analyzed protein expression in granulocytes and the corresponding gene expression in circulating CD34^+^ cells of MPN subjects (Table 4). The common and statistically significant tendencies at mRNA and protein levels, in MPNs ratio, were demonstrated for the following genes: ARP2 actin-related protein 2/3 homolog (ACTR2/3), ATP synthase, H+ transporting beta polypeptide (ATP5B), carbonic anhydrase I (CA1), filamin A, alpha (FLNA), HSP90AB1, moesin (MSN), protein disulfide isomerase family A, member 6 (PDIA6), PPPIA, proteasome activator subunit 2 (PSME2), and tyrosine 3-monooxygenase/tryptophan 5-monooxygenase activation protein, beta polypeptide (YWHAB, Table 4). Contrary, the inverse tendencies in MPNs ratio were demonstrated for annexin A5 (ANXA5), oxidative stress related catalase (CAT), GDP dissociation inhibitor 2 (GDI2), GLRX, heat shock 70kDa protein 1A (HSPA1A), keratin 10 (KRT10), lectin galactoside-binding soluble 1 (LGALS1), leukotriene A4 hydrolase (LTA4H), N-myc downstream regulated 1 (NDRG1), protein tyrosine phosphatase, non-receptor type 6 (PTPN6), RAB10, RAP2B, S100A4/6, and superoxide dismutase 1 (SOD1) genes (Table 4). The expanded list of proteins defined by proteomic analysis and corresponding genes defined by microarray in MPNs were presented in S5 Table. Using proteomic studies, a significant up-regulation was detected for proteins that control myeloid cell apoptosis and proliferation: RAC2, myeloid cell nuclear differentiation antigen (MNDA), S100A8/9, CORO1A, guanine nucleotide binding protein α inhibiting activity polypeptide (GNAI2, S5 Table), observed also by microarray analysis (Table 2). All genes defined by MPNs ratio in proteomic analysis were individually examined by microarray analysis in CD34^+^ cells and observed values were presented as mean difference in S6 Table. The following genes had increased expression both at protein and mRNA levels in JAK2 positive MPN subjects: ANXA1 in ET and PMF; CAI, charged multivesicular body protein 5 (CHMP5), ficolin 1 (FCN1), major vault protein (MVP) in PV; copine III (CPNE3), formin binding protein 1 (FNBP1), S100A4 in PMF; as well as in JAK2 negative MPN subjects: tropomyosin 3 (TPM3), CAT, ACTR2, and LTA4H (Table 4). Genes particularly increased at protein level in granulocytes of JAK2 positive MPNs were: ANXA5, KRT10, LGALS1, splicing factor 3b, subunit 2 (SF3B2) in PMF; chitinase 1 (CHIT1), HSPA1A, PSME2 in ET; and chaperonin containing TCP1, subunit 2 (CCT2) in PV; as well as in JAK2 negative MPN subjects: NDRG1, GDI2, glucose-6-phosphate isomerase (GPI), and PTPN6 (Table 4). In concomitantly analyzed gene expression in CD34^+^ cells of MPNs, the following genes showed highly significant (p<0.01) and increased expression in JAK2 positive MPN subjects: *APEX1*, chromobox homolog 1 (*CBX1*), *FCN1*, *TPM3*, dihydrolipoamide S-succinyltransferase (*DLST*), *GLRX*, malic enzyme 2, NAD(+)-dependent, mitochondrial (ME2), and *RAB10*. The following genes have increased expression in JAK2 negative MPN subjects: *FNBP1*, *GNAI2* and proliferation-associated 2G4 (*PA2G4*, Table 4). Regarding defined genes in granulocytes by proteomic study, the microarray analyses of them were performed in granulocytes of MPN subjects and presented in S7 Table.

**Table 4:** The common statistically significant genes in comparative proteomic (granulocytes) and microarray analyses (CD34^+^ cells).

Ratio	PV / ET		PV / PMF		ET / PMF		PV / Mut0		ET / Mut0		PMF / Mut0	
Genes	P	M	P	M	P	M	P	M	P	M	P	M
ACTR2	1.06	A1					0.94		0.91		0.94	
ACTR3	1.1	A1			0.93			A1	0.92			
ALB	0.9	B1										
ANXA1	0.95		0.97		**1.05**			A1	1.09		1.07	
ANXA2				A1			**0.77**					
ANXA5	0.89		0.83		0.95	A2			1.16		1.22	
APEX1		A2				B1					**0.96**	
ARPC3				A1	0.87							
ARPC5				A1				A1			**0.96**	
ATP5A1		A1							0.92			
ATP5B	1.08	A1							0.92			
ATP6V1E1	**1.11**									A1		
CA1	0.83		1.2	A1	1.44		1.22		1.47			
CANX	**1.08**	A1						A1	0.94			
CAT	0.93	A1					0.9		0.97		0.94	
CBX3		A2		B1				A1	**0.88**			
CCT2	1.12	A2			0.84				0.86			
CHIT1	**0.82**			B1	**1.21**						0.89	
CHMP5	**2.24**							A1				
CPNE3		B1		B2	**0.94**	B1					**1.08**	
DDT	**1.01**	A1							1.02	B1	**1.07**	
DLST								A2			0.91	
ENO1	0.95			B1						B1		
FBP1								B1			**1.2**	
FCN1	1.39			A2	0.74	A1			0.77			
FLNA							0.94	B1	0.94		0.94	
FNBP1								B2		B1	**1.08**	
GAPDH					0.98	B1	0.94		0.94	B1	0.98	
GDI2		A1					0.92	A1			0.94	
GLRX			0.91	A1	0.89			A2			**1.08**	
GNAI2	0.92			A1				B2				B2
GPI	**1.18**		0.81		0.73		**0.85**	B2	0.76	B1		
GSTP1	0.9		0.99					B1	0.94		0.91	
H2AFY	0.9									B1		
HBD	0.63	A1	1.25		1.82			A1	1.61		0.9	
HIST1H2BO	B1						**0.85**				**0.84**	
HSP90AB1	1.08	A1							0.93		**0.96**	
HSPA8								A1			0.98	
HSPA1A	0.84	A1	1.15		1.39		1.13	A1	1.35			
KRT10	1.45		1.38				0.7		0.55	B1	0.56	
LGALS1	0.83		0.64	A2	**0.77**	A2	0.8	B1		B1	**1.3**	B1
LTA4H	0.95						**0.94**	A1			0.94	
ME2	**0.93**							A2				
MIF	0.89									B1		
MSN	0.92	B2							1.06			
MVP	1.2					B1	1.38				1.22	
NDRG1	1.17	B1	0.71		0.66						**0.88**	
PA2G4								B1		B1	**0.96**	
PDIA6	**1.13**	A1										
PPIA	0.91				1.13	A1						
PPIB	0.74				1.5			B1	1.42		0.94	
PSME2					1.23	A2		A1	1.18	A2		
PTPN6					**0.95**		**0.93**		0.91	A1		
RAB10	0.89	A1						A2		A2		
RAP2B	0.8	A1										
RAB8A	0.9									A1		
RHOA	0.95		0.95				0.94					B1
S100A4	0.77		0.8	A1			0.84				**1.06**	
S100A6			0.9	A1								
SF3B2								A1			**1.18**	
SOD1	0.91	A1			1.09						**0.97**	
SPCS2		A1						A1			0.92	
TPM3				A2	0.9		0.89		0.87		**0.96**	
YWHAB				A1			0.89		**0.91**		0.9	B1

Scores—proteomic results (P) bolded (p<0.05) and non bolded (p<0.01); microarray results (M) with ratio > 1 (A1: p<0.05, A2: p<0.01) and ratio < 1 (B1: p<0.05, B2: p<0.01). PV/ET—polycythemia vera / *JAK2V617F*+ essential thrombocythemia ratio, PV/PMF—PV / *JAK2V617F*+ primary myelofibrosis ratio, PV / Mut0—PV / *JAK2V617F*- ET/PMF ratio.

### Expression of genes linked to mTOR signaling pathway in MPNs

**Fig 4:**
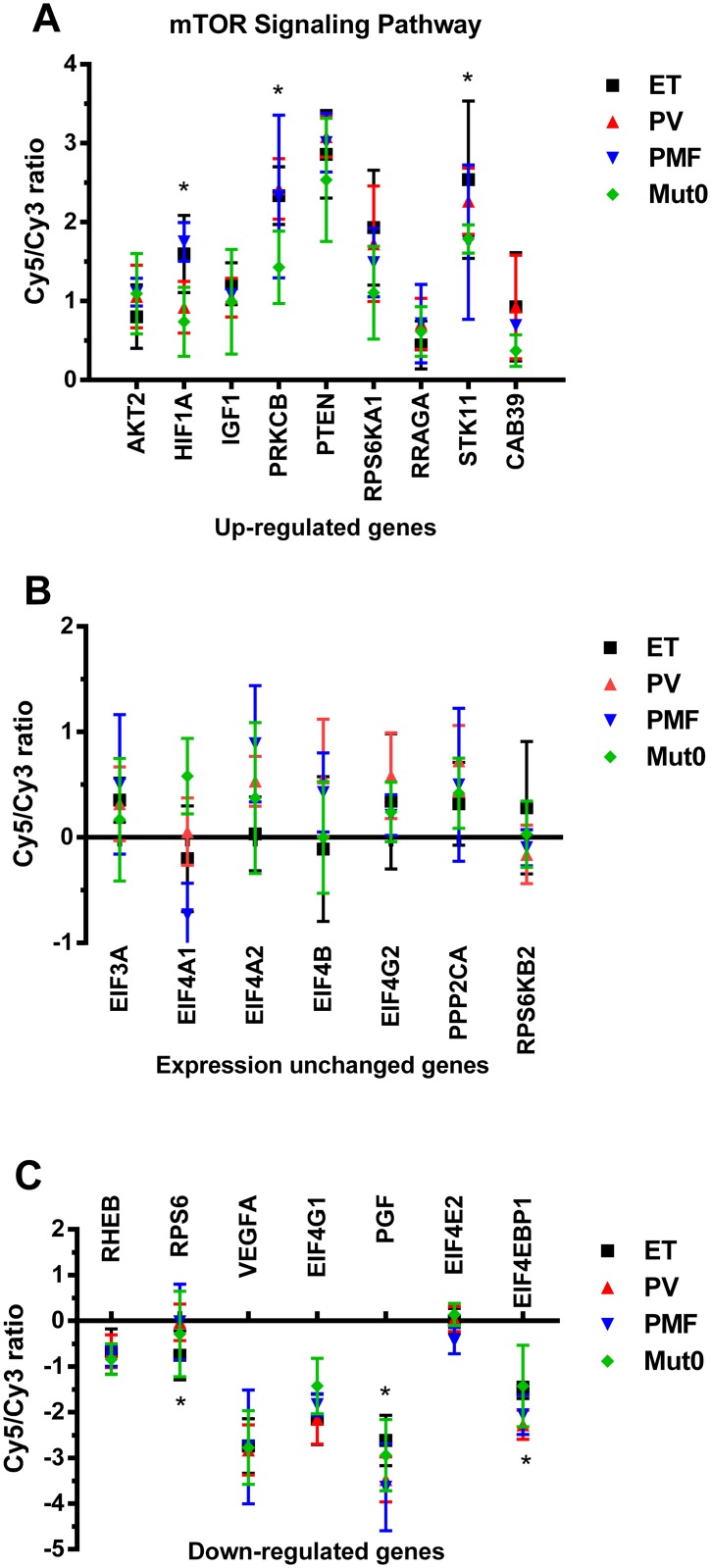
Expression of genes linked to mTOR signaling pathway in CD34^+^ cells of MPN origin. A) Up-regulated genes vs. HuURNA in ET, PV, PMF and ET/PMF subjects without *JAK2V617F* mutation (Mut0); B) Unchanged gene expression vs. HuURNA in ET, PV, PMF and Mut0 subjects; C) Down-regulated genes vs. HuURNA in ET, PV, PMF and Mut0 subjects analyzed by microarray. Values are mean ± SEM (n = 4–9). *P < 0.05 compared among MPNs.

**Fig 5:**
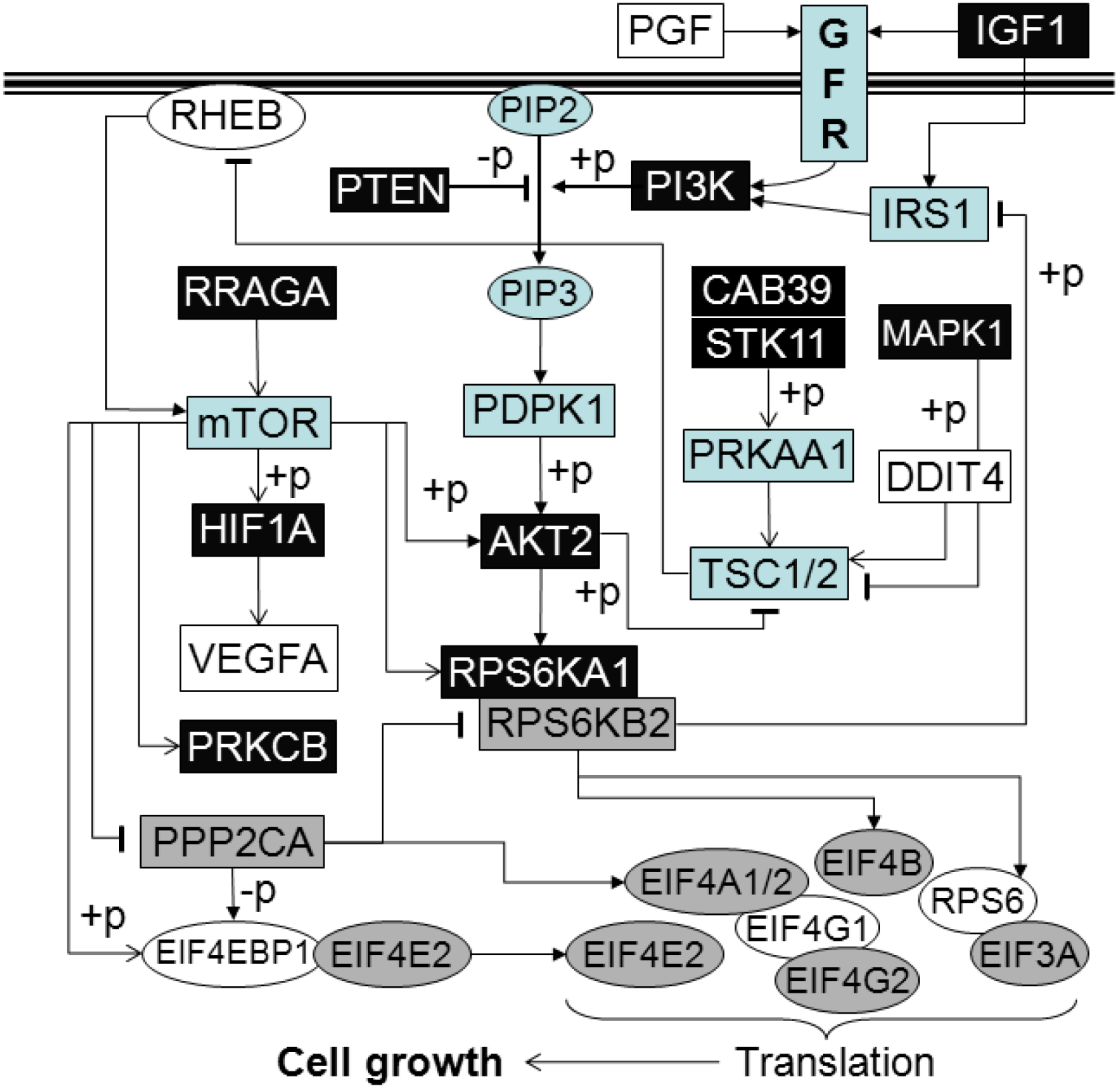
mTOR signaling pathway in CD34^+^ cells of MPN origin. (+p) phosphorylation, (-p) dephosphorylation; → stimulation, ┴ inhibition; white boxes represent down-regulated genes, gray boxes unchanged genes, black boxes up-regulated genes *vs*. HuURNA (corresponding to Fig 4), while blue boxes represent none expressed or sporadically expressed genes in MPNs. GFR—growth factor receptors.

The activity of PI3K/AKT and mTOR was closely related, and interact with MAPK signaling pathway [24]. Using microarray analysis, we analyzed mTOR signaling pathway related genes in CD34^+^ cells of MPNs as presented in Fig 4. Phosphatase and tensin homolog (*PTEN*) and protein kinase C β (*PRKCB*) had the most elevated expression of examined genes in MPNs, while *PRKCB* was significantly more expressed in PV in comparison to *JAK2V617F* negative MPN (Fig 4A). Phosphoinositide-3-kinase, catalytic, α polypeptide (*PIK3CA*) and regulatory subunit 1 (*PIK3R1*) were only overexpressed in PV subjects, as well as MAPK1. Hypoxia inducible factor α (*HIF1α*) was significantly more expressed in JAK2 positive ET and PMF subjects than in other MPN subjects (Fig 4A). Serine/threonine kinase 11 (*STK11*) gene expression was significantly up-regulated in CD34^+^ cells of PV in comparison to *JAK2V617F* negative MPN subjects (Fig 4A). The eukaryotic translation initiation factors did not have changed gene expression (Fig 4B) or were highly down-regulated genes such as eukaryotic translation initiation factor 4E binding protein 1 (*EIF4EBP1*) and 4γ1 (*EIF4G1*), as well as vascular endothelial growth factor A (*VEGFA*, Fig 4C). *EIF4EBP1* and *EIF4G1* genes expression were also down-regulated in granulocytes. *EIF4EBP1* and placental growth factor (*PGF*) gene expression were significantly more down-regulated in PV than ET subjects, while ribosomal protein S6 (*RPS6*) in ET and *JAK2V617F* negative MPNs (Fig 4C). Overview and connection among examined mTOR signaling pathway related genes was presented in Fig 5. Most of the genes were up-regulated and expressed in MPNs, while some of them were down-regulated and not detected. MAPK1 gene expression was increased almost exclusively in PV patients. Major PI3K/AKT signaling pathway related genes were up-regulated, including ribosomal protein S6 kinase, 90kDa, polypeptide 1 (*RPS6KA1*) and Ras-related GTP binding A (*RRAGA*, Fig 5).

## Discussion

Using microarray and proteomic analyses, we illustrated a broad gene and protein expression pattern in MPNs. A two times higher number of genes was detected in primitive CD34^+^ cells compared to mature granulocytes of MPNs. In addition to common genes, we observed distinctive genes for PV, ET, PMF and *JAK2V617F* mutation negative MPNs in CD34^+^ cells. Proteomic analyses revealed proteins with a statistically significant expression in granulocytes of MPN subjects in correlation with *JAK2V617F* mutation. In CD34^+^ cells of JAK2 positive ET subjects, a largely increased gene expression was observed for *CRIP1* and *GADD45B*, in PV subjects for *GLRX*, *IFI16*, *LAMP1*, in PMF subjects for *CPNE3*, *APEX1*, *KDM1A*, while in JAK2 negative MPN subjects for *CIB1*, *NBPF10* and *GPR160*. After hierarchical clustering, the arrays of statistically significant genes largely demonstrated tendency to stay gathered within the examined 4 MPN groups. We identified over-expression of genes that regulate myeloid cell proliferation (*MAS1*, *RAC2*), differentiation (*RUNX1*, *HCLS1*) and apoptosis (*S100A8*, *TNFRSF19*) of MPNs. Interestingly, a large number of mTOR signaling pathway related genes was observed in MPNs with up-regulated PI3K/AKT signaling.

Previous profile reports included various quantities of examined genes between 6000 and 14500, from purified primary cells of MPNs [11–19]. We performed parallel proteomic and microarray studies from the same MPN subjects to strengthen the comparability of a broad gene expression study with 25,100 genes. Actually, we explored both primitive CD34^+^ cells and mature granulocytes, isolated simultaneously from the peripheral blood of MPN subjects, to illustrate a continuous gene expression profile during hematopoiesis.

The PI3K/AKT/mTOR pathway is commonly activated in human cancers and has a vital role in cell growth, proliferation, survival, apoptosis, and angiogenesis [25]. The inhibition of PI3K or mTOR corrected the expansive myelopoiesis in the PTEN-deficient embryos [26]. The inactivation of PTEN activity was a result of PTEN phosphorylation associated with AKT phosphorylation and shorter overall survival. PTEN suppressed apoptosis and regulated cell growth controlling p70 S6 kinase (S6K) in the cytoplasm, while regulated cell cycle progression in the nucleus [27]. The activity of p70 S6K was controlled by multiple phosphorylation events mediated by mTOR [11]. The inhibition of PI3K/AKT/mTOR signaling suppressed the ribosomal protein S6 (RPS6) in acute leukemia subjects [28]. RPS6, 70 kDa, polypeptide 2 (RPS6KB2) and PTEN were strongly associated with the activation of the mTOR signaling pathway in cancer, while mTOR pathway activation demonstrated higher expression of *RPS6K*, *S6*, *4E-BP1* and *eIF-4G*, linked with more aggressive clinical behavior [29]. The phenotypic expression of the cancer genome involved translation by the eIF4E2-directed hypoxic protein synthesis mechanism [30]. *PTEN* was generally highly expressed in MPN, according to our results, while *RPS6* was down-regulated in JAK2 negative MPNs. *EIF4G1* gene expression was also down-regulated in MPN.

mTOR is a serine/threonine protein kinase that forms two distinct signaling complexes, mTORC1 and mTORC2, distinguished by their association with Raptor or Rictor, respectively. Growth factors, as important stimuli of the PI3K and Ras small GTPase pathways, activated the Akt and ERK serine/threonine kinases, derepressing Ras homolog enriched in brain (Rheb) and promoting mTORC1 signaling [31]. Rheb stimulated the activation of mTOR with an essential role in the regulation of S6K and 4EBP1 [32]. In presented results, *RHEB* gene expression was decreased, while *PTEN* increased in CD34^+^ cells. Tuberous sclerosis proteins 1 and 2 (TSC1/2) complex inhibited mTORC1 signaling through its GAP activity towards Rheb [33]. According to our results, the mTOR activation was stimulated by PI3K/AKT dependent inhibition of TSC1/2 complex and *RHEB*. mTOR signaling induced *RPS6KA1* gene expression was also up-regulated by the PI3K/AKT signaling pathway.

Increased erythropoiesis in PV was related to augmented phosphorylation of AKT [34]. It has been also demonstrated that mTOR regulated proliferation of megakaryocyte progenitors and the late stages of megakaryocyte differentiation. The mTOR was a critical downstream target of the thrombopoietin receptor, demonstrating contributions in the proliferation and differentiation of normal megakaryocyte cultures [35]. Phosphorylation of mTOR downstream effectors p70 S6K and 4E-BP1 by thrombopoietin demonstrated PI3K/mTOR dependence [36]. The increased mTOR phosphorylation has been observed during megakaryopoiesis of CD34^+^ cells isolated from ET and PMF subjects, as well as in immunohistochemical analyses performed on bone marrow samples of ET and PMF subjects [37]. In the presented results, *EIF4EBP1* gene expression was also down-regulated in MPNs, significantly more in PV than ET subjects.

The co-treatment of dual PI3K/mTOR inhibitor BEZ235 and JAK1/2 inhibitor ruxolitinib was more effective than single drugs in reducing the degree of disease and prolonging survival in conditional *JAK2V617F* knock-in mice [38]. JAK1/2 inhibitor inactivated the mTOR/p70S6K/4EBP1 pathway and reduced the inhibitory phosphorylation of GSK3 in leukemia cell line, which correlated with JAK1/2 inhibitor reduction of proliferation and survival of these cells [39]. PI3K/AKT/mTOR pathway inhibitor everolimus inhibited cell proliferation and clonogenic potential in human *JAK2V617F* mutated cell lines. In subjects with PV and PMF, hematopoietic progenitors were significantly more sensitive to everolimus than healthy control subjects. A multicenter phase 1/2 trial with everolimus in PMF demonstrated a well-tolerated clinical efficiency in terms of spleen size reduction and resolution of systemic symptoms and pruritus [40]. These reports showed that the PI3K/AKT/mTOR pathway may be a new target for treatment in MPN, in accordance with mTOR’s essential role for expansion of CD34^+^ cells during myelopoiesis [41].

In conclusion, investigation of the extent by which common or specific genes participate in the complex molecular and cellular mechanisms of MPN will lead to new insights of malignancy development. The molecular profiling of CD34^+^ cells and granulocytes of MPN subjects, in our study, revealed a gene expression pattern that was not previously recognized in disease pathogenesis. This profile may be helpful and related to subjects’ clinical characteristics with imminent prognostic relevance. In addition, our study releases that modulation of the mTOR signaling pathway may offer a new approach in therapy of MPNs.

## Supporting Information

S1 Table The correlation summary report among the MPN samples in CD34^+^ cells. (DOCX)

S2 Table Genes up-regulated more than 4 fold at least in one of MPN subtypes in CD34^+^ cells and granulocytes determined by microarray analyses. (DOCX)

S3 Table Genes down-regulated more than 3 fold at least in one of MPN subtypes in CD34^+^ cells and granulocytes determined by microarray analyses. (DOCX)

S4 Table The statistically significant genes (p<0.01) among MPNs in granulocytes determined by microarray analysis. (DOCX)

S5 Table Comparison of statistically significant protein expression in granulocytes, determined by proteomic analysis, with their counterpart gene expression determined by microarray analysis in CD34^+^ cells of MPNs. (DOCX)

S6 Table Gene expression previously determined by proteomic studies in CD34^+^ cells of MPNs, analyzed by microarray. (DOCX)

S7 Table Gene expression previously determined by proteomic studies in granulocytes of MPNs, analyzed by microarray. (DOCX)
