# Supplementary material for: Microarray and Proteomic Analyses of Myeloproliferative Neoplasms with a Highlight on the mTOR Signaling Pathway
Source: PLoS One. 2015 Aug 14;10(8):e0135463. doi: 10.1371/journal.pone.0135463 (PMC4537205; doi:10.1371/journal.pone.0135463)
Supplement: S1 Table — (DOCX) [file pone.0135463.s001.docx]

**S1 Table.** The correlation summary report among the biological replicates of MPN samples in CD34^+^ cells.

| **A1** | **A2** | **A3** | **A4** | **A5** | **B6** | **B7** | **B8** | **B9** | **B10** | **B11** | **B12** | **C13** | **C14** | **C15** | **D16** | **D17** | **D18** | **D19** | **D20** |  |
| --- | --- | --- | --- | --- | --- | --- | --- | --- | --- | --- | --- | --- | --- | --- | --- | --- | --- | --- | --- | --- |
| **A1** | 0.90 | 0.81 | 0.87 | 0.92 | 0.90 | 0.93 | 0.91 | 0.89 | 0.93 | 0.91 | 0.87 | 0.81 | 0.79 | 0.78 | 0.92 | 0.84 | 0.77 | 0.86 | 0.93 | **A1** |
|  | **A2** | 0.86 | 0.90 | 0.89 | 0.91 | 0.90 | 0.92 | 0.96 | 0.89 | 0.88 | 0.82 | 0.89 | 0.72 | 0.74 | 0.85 | 0.83 | 0.87 | 0.86 | 0.91 | **A2** |
|  |  | **A3** | 0.86 | 0.84 | 0.87 | 0.81 | 0.91 | 0.82 | 0.82 | 0.77 | 0.72 | 0.93 | 0.68 | 0.60 | 0.73 | 0.92 | 0.92 | 0.89 | 0.82 | **A3** |
|  |  |  | **A4** | 0.88 | 0.86 | 0.86 | 0.89 | 0.86 | 0.83 | 0.83 | 0.78 | 0.88 | 0.67 | 0.72 | 0.84 | 0.82 | 0.87 | 0.81 | 0.89 | **A4** |
|  |  |  |  | **A5** | 0.92 | 0.94 | 0.92 | 0.88 | 0.93 | 0.93 | 0.89 | 0.86 | 0.83 | 0.83 | 0.94 | 0.86 | 0.76 | 0.85 | 0.94 | **A5** |
|  |  |  |  |  | **B6** | 0.94 | 0.95 | 0.90 | 0.92 | 0.91 | 0.87 | 0.89 | 0.81 | 0.82 | 0.89 | 0.89 | 0.80 | 0.87 | 0.93 | **B6** |
|  |  |  |  |  |  | **B7** | 0.94 | 0.90 | 0.94 | 0.95 | 0.92 | 0.84 | 0.83 | 0.85 | 0.94 | 0.83 | 0.74 | 0.84 | 0.94 | **B7** |
|  |  |  |  |  |  |  | **B8** | 0.90 | 0.93 | 0.89 | 0.87 | 0.93 | 0.78 | 0.77 | 0.88 | 0.89 | 0.86 | 0.90 | 0.92 | **B8** |
|  |  |  |  |  |  |  |  | **B9** | 0.91 | 0.90 | 0.85 | 0.85 | 0.75 | 0.79 | 0.86 | 0.80 | 0.80 | 0.82 | 0.91 | **B9** |
|  |  |  |  |  |  |  |  |  | **B10** | 0.93 | 0.93 | 0.84 | 0.85 | 0.85 | 0.92 | 0.85 | 0.77 | 0.87 | 0.92 | **B10** |
|  |  |  |  |  |  |  |  |  |  | **B11** | 0.90 | 0.80 | 0.82 | 0.86 | 0.93 | 0.79 | 0.70 | 0.79 | 0.95 | **B11** |
|  |  |  |  |  |  |  |  |  |  |  | **B12** | 0.75 | 0.90 | 0.92 | 0.91 | 0.76 | 0.66 | 0.79 | 0.89 | **B12** |
|  |  |  |  |  |  |  |  |  |  |  |  | **C13** | 0.66 | 0.66 | 0.76 | 0.87 | 0.92 | 0.88 | 0.86 | **C13** |
|  |  |  |  |  |  |  |  |  |  |  |  |  | **C14** | 0.88 | 0.85 | 0.75 | 0.56 | 0.70 | 0.81 | **C14** |
|  |  |  |  |  |  |  |  |  |  |  |  |  |  | **C15** | 0.88 | 0.67 | 0.51 | 0.65 | 0.85 | **C15** |
|  |  |  |  |  |  |  |  |  |  |  |  |  |  |  | **D16** | 0.79 | 0.65 | 0.85 | 0.93 | **D16** |
|  |  |  |  |  |  |  |  |  |  |  |  |  |  |  |  | **D17** | 0.84 | 0.87 | 0.84 | **D17** |
|  |  |  |  |  |  |  |  |  |  |  |  |  |  |  |  |  | **D18** | 0.87 | 0.77 | **D18** |
|  |  |  |  |  |  |  |  |  |  |  |  |  |  |  |  |  |  | **D19** | 0.83 | **D19** |
|  |  |  |  |  |  |  |  |  |  |  |  |  |  |  |  |  |  |  | **D20** | **D20** |

А – ЕТ JAK2+, B – PV JAK2+, C – PMF JAK2+, D – ET/PMF JAK2 (Mut0)
