# Supplementary material for: Microarray and Proteomic Analyses of Myeloproliferative Neoplasms with a Highlight on the mTOR Signaling Pathway
Source: PLoS One. 2015 Aug 14;10(8):e0135463. doi: 10.1371/journal.pone.0135463 (PMC4537205; doi:10.1371/journal.pone.0135463)
Supplement: S2 Table — (DOCX) [file pone.0135463.s002.docx]

**S2 Table.** Genes upregulated more than 4 fold at least in one of MPN subtypes in CD34^+^ cells and granulocytes determined by microarray analyses.

| **Genes** | **Cells** | **PV** | | **ET** | | **PMF** | | **Mut0** | |
| --- | --- | --- | --- | --- | --- | --- | --- | --- | --- |
|  |  | **Mean** | SD | **Mean** | SD | **Mean** | SD | **Mean** | SD |
| ACRV1 | CD34 | 5.46 | *0.60* | 5.68 | *0.44* | 6.17 | *0.18* | 5.83 | *0.79* |
|  | **GRA** | **5.78** | ***1.27*** | **5.51** | ***0.64*** | **5.84** | ***0.59*** | **4.83** | ***0.99*** |
| ADAM21 | CD34 | 3.73 | *1.02* | 4.71 | *0.93* | 4.39 |  | 4.73 | *0.05* |
| ALOXE3 | CD34 | 3.89 | *0.75* | 4.16 | *1.04* | 5.03 | *0.53* | 4.30 | *0.04* |
|  | **GRA** | **5.55** | ***1.12*** | **5.08** | ***0.50*** | **4.65** |  | **5.71** |  |
| AMICA1 | CD34 | 4.31 | *0.40* | 4.16 | *0.55* |  |  | 2.31 | *0.56* |
| ASTN2 | **GRA** | **5.16** | ***0.38*** | **5.20** | ***0.12*** | **4.08** | ***1.29*** | **5.30** | ***0.50*** |
| ATG4C | CD34 | 4.05 | *0.65* | 4.12 | *1.11* | 5.32 | *0.29* | 3.74 | *1.11* |
|  | **GRA** | **5.90** | ***0.79*** | **5.15** | ***0.07*** | **4.65** | ***0.46*** | **4.90** | ***0.23*** |
| B4GALT6 | CD34 | 5.38 | *0.54* | 5.36 | *0.79* | 5.61 | *0.04* | 4.94 | *0.69* |
|  | **GRA** | **6.64** | ***0.68*** | **6.61** | ***0.17*** | **6.34** | ***0.32*** | **6.19** |  |
| BNIP3L | **GRA** | **3.46** | ***0.87*** | **4.13** | ***0.19*** | **2.91** | ***1.19*** | **4.29** | ***0.13*** |
| C1orf162 | CD34 | 5.16 | *0.25* | 4.18 | *0.81* |  |  |  |  |
| C3orf70 | CD34 | 5.66 | *0.66* | 5.62 | *1.03* | 5.88 | *0.18* | 5.52 | *0.64* |
|  | **GRA** | **5.94** | ***1.08*** | **5.68** | ***0.74*** | **5.73** | ***0.44*** | **4.67** | ***1.00*** |
| C9orf163 | **GRA** | **5.21** | ***0.47*** | **4.46** | ***0.02*** | **4.13** | ***0.31*** | **4.29** | ***0.21*** |
| CARD16 | CD34 | 4.23 | *0.68* | 3.59 | *0.76* | 3.89 |  | 2.07 | *0.89* |
| CASP1 | CD34 | 4.61 | *0.51* | 4.17 | *1.20* | 4.24 |  | 3.10 | *1.07* |
| CCDC144B | **GRA** | **4.02** | ***0.22*** | **4.38** |  | **3.00** | ***0.99*** | **4.26** |  |
| CCDC88A | CD34 | 4.06 | *0.64* | 4.06 | *0.83* | 5.32 |  | 3.35 | *1.13* |
|  | **GRA** | **5.02** | ***0.73*** | **4.87** | ***0.05*** | **5.16** | ***0.11*** | **5.08** | ***0.44*** |
| CCR2 | CD34 | 4.90 | *0.51* | 3.75 | *0.90* | 3.83 |  |  |  |
| CD48 | CD34 | 5.41 | *0.50* | 4.85 | *0.97* | 4.96 |  |  |  |
| CD52 | CD34 | 4.31 | *0.42* | 4.43 | *0.61* | 4.46 | *0.17* | 4.50 | *0.14* |
| CLEC3B | **GRA** | **4.22** | ***0.78*** | **3.34** | ***0.41*** | **3.78** | ***0.29*** | **4.00** | ***0.64*** |
| CORO1A | CD34 | 4.15 | *0.44* | 4.02 | *0.52* | 3.57 | *0.14* | 4.01 | *0.95* |
| CPPED1 | CD34 | 4.16 | *1.01* | 2.92 | *1.02* | 4.43 |  | 3.93 | *0.74* |
| CRABP2 | **GRA** | **3.87** | ***1.23*** | **4.19** | ***0.25*** | **3.33** | ***0.19*** | **3.41** | ***0.51*** |
| CRELD1 | CD34 | 5.89 | *0.69* | 5.84 | *0.97* | 6.41 | *0.50* | 5.45 | *0.83* |
|  | **GRA** | **6.37** | ***1.77*** | **5.91** | ***0.51*** | **6.28** | ***0.13*** | **5.15** | ***1.29*** |
| CTSS | CD34 | 5.70 | *0.48* | 5.36 | *0.83* | 4.89 | *1.18* | 5.28 | *0.52* |
| DNASE1 | CD34 | 3.16 | *0.97* | 4.27 | *0.84* | 5.33 | *0.18* | 4.48 | *0.01* |
|  | **GRA** | **5.39** | ***1.27*** | **3.66** |  | **3.68** | ***0.63*** | **4.84** |  |
| DNM1P46 | **GRA** | **4.07** | ***0.81*** | **4.70** | ***0.10*** | **3.72** | ***0.63*** | **4.76** | ***0.07*** |
| DUSP1 | CD34 | 4.19 | *1.05* | 4.35 | *0.93* | 4.85 |  | 3.66 | *0.89* |
| FAM26F | CD34 | 4.48 | *0.56* | 3.27 | *0.70* | 3.63 | *0.23* | 2.50 | *0.34* |
| FCER1G | CD34 | 4.50 | *0.92* | 4.17 | *0.80* | 5.26 |  | 3.03 | *1.97* |
| FCN1 | CD34 | 4.35 | *0.33* | 3.66 | *0.27* | 3.21 | *0.81* | 3.92 | *0.55* |
| FGL2 | CD34 | 4.17 | *0.57* | 3.54 | *0.83* | 3.97 |  |  |  |
| FLJ42200 | **GRA** | **4.59** | ***0.64*** | **4.89** | ***0.17*** | **3.96** | ***1.47*** | **5.05** | ***0.53*** |
| FLJ44796 | **GRA** | **3.96** | ***0.58*** | **4.03** | ***0.46*** | **3.63** | ***1.41*** | **4.14** | ***0.34*** |
| FNDC8 | CD34 | 2.63 | *0.78* | 3.80 | *1.02* | 4.55 | *0.48* | 3.98 |  |
| FPR1 | CD34 | 4.86 | *0.48* | 4.59 | *1.30* |  |  |  |  |
| FREM2 | **GRA** | **4.28** | ***0.97*** | **4.44** | ***0.14*** | **3.44** | ***1.50*** | **4.27** | ***0.38*** |
| GBP5 | CD34 | 5.07 | *0.76* | 4.48 | *1.13* | 5.33 |  | 4.25 |  |
| GLYR1 | **GRA** | **4.43** | ***0.63*** | **3.98** | ***0.35*** | **3.98** | ***0.86*** | **4.24** | ***0.47*** |
| GNPDA1 | CD34 | 3.31 | *0.77* | 3.89 | *1.31* | 4.92 | *0.51* | 2.77 | *1.77* |
|  | **GRA** | **4.70** | ***1.13*** | **5.21** | ***0.16*** | **4.18** | ***0.63*** | **4.37** | ***0.33*** |
| HBB | CD34 | 6.98 | *0.86* | 5.77 | *1.53* | 6.52 |  | 6.45 | *0.16* |
| HCLS1 | CD34 | 4.36 | *0.35* | 4.28 | *0.76* | 3.77 | *0.10* | 3.70 | *0.81* |
| HLA-DMA | CD34 | 4.38 | *0.37* | 4.35 |  | 4.24 |  | 2.97 |  |
| HLA-DPA1 | CD34 | 5.01 | *0.46* | 4.91 | *0.57* | 4.87 |  | 4.99 | *0.09* |
| HLA-DPB1 | CD34 | 4.51 | *0.56* | 3.84 | *0.94* | 4.77 | *0.31* | 4.61 | *0.04* |
| HLA-DQB1 | CD34 | 5.32 | *0.37* | 4.42 | *0.95* | 5.83 |  | 4.33 | *0.74* |
| HLA-DRA | CD34 | 6.96 | *0.53* | 6.75 | *0.92* | 6.97 | *0.12* | 6.79 | *0.48* |
| HLA-DRB1 | CD34 | 5.71 | *0.35* | 4.77 | *1.01* | 2.06 | *0.23* | 3.24 | *0.57* |
| HLA-DRB3 | CD34 | 5.44 | *0.26* | 5.75 | *0.67* | 5.36 | *0.19* | 5.57 | *0.49* |
| HLA-DRB4 | CD34 | 5.19 | *0.41* | 4.61 | *1.09* | 5.32 |  | 5.63 |  |
| HMCN1 | CD34 | 5.38 | *0.67* | 5.08 | *0.77* | 6.03 | *0.37* | 5.17 | *0.15* |
|  | **GRA** | **6.85** | ***0.76*** | **8.27** |  | **6.37** | ***0.03*** | **7.11** |  |
| HMGN2 | CD34 | 4.86 | *0.61* | 5.15 | *1.09* | 5.85 | *0.44* | 4.68 | *1.11* |
|  | **GRA** | **4.83** | ***1.13*** | **4.64** | ***0.11*** | **5.50** | ***0.83*** | **4.26** | ***0.57*** |
| HMX1 | CD34 | 4.75 | *0.64* | 4.93 | *0.38* | 5.39 | *0.01* | 4.69 | *0.36* |
|  | **GRA** | **5.57** | ***0.96*** | **5.89** | ***0.26*** | **5.43** | ***0.10*** | **5.93** |  |
| HOXA4 | **GRA** | **3.24** | ***0.05*** | **4.31** | ***0.04*** | **3.47** | ***0.40*** | **3.85** |  |
| HOXB-AS3 | **GRA** | **3.85** | ***0.23*** | **4.09** | ***0.04*** | **3.17** |  |  |  |
| HSP90AB2P | **GRA** | **4.75** | ***0.53*** | **4.90** | ***0.18*** | **4.08** | ***1.42*** | **4.79** | ***0.58*** |
| HSPA2 | **GRA** | **3.27** | ***0.25*** | **4.59** | ***0.46*** | **3.32** | ***0.13*** | **3.71** | ***0.47*** |
| HYI | CD34 |  |  | 4.01 | *1.17* |  |  | 4.55 |  |
| IGHG1 | CD34 | 6.49 | *0.71* | 6.09 | *1.28* |  |  | 6.99 |  |
| JAK2 | CD34 | 4.02 | *0.45* | 3.54 | *0.81* | 3.01 |  |  |  |
| KCNQ1OT1 | **GRA** | **4.62** | ***0.71*** | **4.72** | ***0.10*** | **3.75** | ***1.32*** | **4.59** | ***0.47*** |
| KIAA1279 | CD34 | 3.07 | *1.32* | 3.62 | *1.55* | 4.40 | *0.94* | 2.95 | *1.98* |
|  | **GRA** | **4.40** | ***1.09*** | **3.07** |  | **3.45** |  | **4.94** |  |
| KIAA1432 | CD34 | 4.02 | *0.80* | 4.87 | *0.78* | 5.48 |  | 4.72 | *0.26* |
|  | **GRA** | **6.04** | ***0.78*** |  |  | **4.25** |  | **5.54** |  |
| KIAA1841 | CD34 | 5.20 | *0.64* | 5.48 | *0.67* | 6.01 | *0.33* | 4.96 | *0.49* |
| KLF2 | CD34 | 5.98 | *0.52* | 5.81 | *0.92* | 5.08 |  | 6.34 | *0.12* |
| KLF6 | CD34 | 4.11 | *0.22* | 3.68 | *0.90* | 3.30 | *0.25* | 4.49 | *0.29* |
| LAPTM5 | CD34 | 4.18 | *0.51* | 4.11 | *0.71* | 4.14 | *0.02* | 4.34 | *0.38* |
| LCP1 | CD34 | 4.56 | *0.57* | 4.33 | *0.58* | 4.22 | *0.32* | 4.26 | *0.61* |
| LPAR2 | CD34 | 4.45 | *0.28* |  |  |  |  |  |  |
| LRFN1 | **GRA** | **4.97** | ***1.17*** |  |  | **5.04** |  | **4.56** |  |
| MAGEB18 | CD34 | 3.96 | *0.57* | 4.51 | *0.78* | 4.98 |  | 4.59 |  |
| MALAT1 | **GRA** | **4.05** | ***0.77*** | **4.61** | ***0.41*** | **3.63** | ***0.14*** | **3.99** | ***0.74*** |
| MAPK10 | **GRA** | **4.55** | ***0.33*** | **4.72** | ***0.11*** | **4.03** | ***0.56*** | **4.62** | ***0.03*** |
| MAS1 | CD34 | 4.12 | *0.92* | 5.00 | *0.77* | 5.49 | *0.14* | 4.95 | *0.19* |
| MED12L | CD34 | 4.94 | *0.58* | 5.24 | *0.93* | 5.51 | *0.13* | 4.96 | *0.13* |
|  | **GRA** | **6.20** | ***0.79*** | **5.93** | ***0.32*** | **5.55** | ***0.18*** | **5.70** |  |
| MIPOL1 | **GRA** | **4.17** | ***0.41*** | **4.44** | ***0.09*** | **3.42** | ***1.47*** | **4.14** | ***0.56*** |
| MLLT4 | **GRA** | **4.35** | ***0.62*** | **4.80** | ***0.19*** | **4.01** | ***1.27*** | **4.38** | ***0.26*** |
| NCF2 | CD34 | 4.14 | *0.59* | 3.30 | *0.93* | 4.25 |  | 2.10 |  |
| NEK6 | CD34 | 5.33 | *0.54* | 5.36 | *1.09* | 5.73 | *0.34* | 5.34 | *0.54* |
|  | **GRA** | **5.57** | ***1.27*** | **5.33** | ***0.47*** | **5.65** | ***0.26*** | **5.16** | ***0.77*** |
| NFE2 | CD34 | 0.63 | *0.35* | 1.10 | *0.38* | 1.137 | *0.57* | 0.70 | *0.54* |
| NFKBIA | CD34 | 3.62 | *0.73* | 4.21 | *1.04* | 3.34 | *0.51* | 3.30 | *1.10* |
| NKAPP1 | **GRA** | **4.02** | ***1.21*** | **2.89** | ***0.66*** | **1.71** | ***1.11*** | **3.70** | ***0.87*** |
| NLRP8 | CD34 | 3.97 | *0.54* | 4.45 | *0.77* | 5.21 | *0.44* | 4.06 | *0.44* |
|  | **GRA** | **5.72** | ***0.79*** | **4.84** |  | **4.99** |  | **5.37** |  |
| NPC1 | CD34 | 2.40 | *0.91* | 3.87 | *0.20* | 4.27 | *0.40* | 3.76 | *0.25* |
|  | **GRA** | **4.20** | ***1.70*** | **2.41** |  | **3.26** |  | **3.58** |  |
| OFCC1 | CD34 | 4.90 | *0.34* | 5.12 | *0.71* | 5.27 | *0.15* | 4.53 | *0.55* |
|  | **GRA** | **6.37** | ***0.64*** | **5.46** | ***0.33*** | **5.42** | ***0.04*** | **5.09** | ***0.46*** |
| ORC4 | **GRA** | **4.52** | ***0.58*** | **4.90** | ***0.20*** | **4.13** | ***1.06*** | **4.50** | ***0.38*** |
| PBX4 | CD34 | 3.47 | *0.86* | 4.02 | *1.06* | 5.04 | *0.38* | 4.28 | *0.30* |
| PCDH1 | CD34 | 3.06 | *0.66* | 3.47 | *0.87* | 4.57 | *0.33* | 2.48 | *0.94* |
| PCYT1A | **GRA** | **3.85** | ***0.56*** | **4.06** | ***0.06*** | **3.35** | ***0.86*** | **3.58** |  |
| PDPR | CD34 | 3.61 | *0.57* | 4.12 | *0.72* | 4.17 | *0.51* | 3.36 | *0.80* |
|  | **GRA** | **4.76** | ***0.07*** | **4.73** | ***0.18*** | **4.50** | ***0.35*** | **4.90** | ***0.20*** |
| PF4 | CD34 | 6.39 | *0.40* | 6.73 | *0.98* | 7.37 |  | 6.37 | *1.61* |
| PGM5P2 | **GRA** | **4.14** | ***0.57*** | **4.00** | ***0.49*** | **3.62** | ***1.41*** | **4.10** | ***0.44*** |
| PIGZ | CD34 | 5.33 | *0.64* | 5.41 | *0.73* | 5.74 | *0.14* | 5.24 | *0.51* |
|  | **GRA** | **5.56** | ***1.12*** | **5.57** | ***0.84*** | **5.81** | ***0.15*** | **4.79** | ***0.95*** |
| PLEK | CD34 | 4.99 | *0.56* | 4.86 | *1.45* | 5.43 |  | 4.23 | *0.59* |
| PLXNA2 | CD34 | 5.44 | *0.59* | 5.28 | *0.88* | 5.81 | *0.04* | 5.32 | *0.53* |
|  | **GRA** | **5.88** | ***1.33*** | **5.23** | ***0.56*** | **5.56** | ***0.50*** | **5.17** | ***0.43*** |
| PP14571 | CD34 | 6.24 | *0.72* | 6.21 | *0.98* | 6.28 | *0.28* | 5.96 | *1.00* |
|  | **GRA** | **5.90** | ***1.53*** | **6.15** | ***0.74*** | **6.47** | ***0.51*** | **4.58** | ***1.54*** |
| PPBP | CD34 | 5.38 | *0.73* | 4.31 | *0.90* | 6.98 |  | 6.82 | *0.80* |
| PPP1R3F | **GRA** | **5.40** | ***0.52*** | **5.61** | ***0.43*** | **4.81** | ***1.38*** | **5.40** | ***0.44*** |
| RGS2 | CD34 | 5.22 | *0.67* | 5.03 | *0.84* | 3.60 | *1.82* | 4.34 | *1.04* |
| RUNX1 | CD34 | 4.54 | *0.45* | 4.85 | *0.50* | 5.34 | *0.25* | 4.47 | *0.35* |
|  | **GRA** | **5.81** | ***1.05*** | **5.71** |  | **5.18** | ***0.15*** | **5.74** |  |
| S100A12 | CD34 | 4.68 | *0.92* |  | *1.32* |  |  | 5.49 |  |
| S100A8 | CD34 | 6.07 | *0.46* | 5.67 | *1.16* | 6.36 | *0.17* | 5.58 | *0.26* |
| S100A9 | CD34 | 4.98 | *0.40* | 5.14 | *0.60* | 4.43 | *0.62* | 4.95 | *0.65* |
|  | **GRA** | **5.10** | ***0.20*** | **2.04** | ***0.33*** | **5.64** | ***1.53*** | **2.52** | ***1.06*** |
| SAT1 | CD34 | 3.63 | *0.63* | 4.12 | *0.99* | 1.93 | *1.17* | 3.01 | *0.76* |
| SERPINB9 | **GRA** | **3.70** | ***0.30*** | **3.76** | ***0.14*** | **2.88** | ***1.22*** | **4.15** | ***0.45*** |
| SNCB | CD34 | 5.45 | *0.46* | 5.34 | *0.64* | 5.82 | *0.23* | 5.19 | *0.77* |
|  | **GRA** | **6.55** | ***0.87*** | **6.45** | ***0.22*** | **5.71** | ***0.74*** | **6.41** | ***0.42*** |
| SRGN | CD34 | 4.51 | *0.84* | 4.41 | *0.71* | 4.80 | *0.61* | 4.65 | *0.68* |
| STAB1 | CD34 | 4.61 | *0.84* | 4.87 | *0.68* | 5.39 | *0.57* | 4.84 | *0.04* |
| STAT1 | CD34 | 4.76 | *0.50* | 4.68 | *0.85* | 4.18 | *0.39* | 3.35 | *1.10* |
| STRADB | CD34 | 5.08 | *0.53* | 5.18 | *0.90* | 5.65 | *0.03* | 4.28 | *1.38* |
|  | **GRA** | **6.28** | ***0.69*** | **5.76** | ***0.03*** | **5.57** | ***0.04*** | **5.74** | ***0.16*** |
| TAS2R1 | CD34 | 4.51 | *0.75* | 4.50 | *1.02* | 5.80 |  | 4.71 | *0.37* |
|  | **GRA** | **6.14** | ***0.82*** | **5.41** | ***0.13*** |  |  | **4.85** | ***0.65*** |
| THBS3 | CD34 | 4.89 | *0.68* | 5.41 | *0.81* | 5.75 | *0.35* | 4.93 | *0.20* |
|  | **GRA** | **6.26** | ***0.76*** | **5.78** | ***0.09*** | **5.67** | ***0.16*** | **6.14** |  |
| TMEM209 | **GRA** | **4.31** | ***0.33*** | **4.84** |  | **2.92** |  | **4.30** | ***0.18*** |
| TNFRSF19 | CD34 | 2.27 | *1.08* | 2.98 | *1.10* | 4.11 | *0.61* | 2.34 | *1.34* |
|  | **GRA** | **4.36** | ***1.04*** | **4.33** | ***0.35*** | **3.59** | ***0.80*** | **4.55** | ***0.43*** |
| TOB1 | CD34 | 2.33 | *1.09* | 2.72 | *1.30* | 4.15 | *0.23* | 2.87 | *0.63* |
| TRPV1 | CD34 | 5.30 | *0.73* | 5.25 | *0.74* | 5.76 | *0.25* | 4.99 | *0.40* |
|  | **GRA** | **5.62** | ***1.10*** | **5.61** | ***0.52*** | **5.70** | ***0.25*** | **4.45** | ***1.06*** |
| TSC22D3 | CD34 | 4.04 | *0.43* | 3.52 | *0.69* | 3.54 |  | 3.33 | *0.57* |
| TSPYL4 | CD34 | 4.73 | *0.68* | 4.75 | *0.87* | 5.38 | *0.26* | 4.24 | *0.96* |
|  | **GRA** | **5.31** | ***1.02*** | **4.98** |  | **4.89** | ***0.29*** | **4.97** | ***0.41*** |
| TTC13 | CD34 | 5.32 | *0.72* | 5.34 | *0.72* | 5.85 | *0.38* | 5.14 | *0.74* |
|  | **GRA** | **6.20** | ***0.78*** | **6.47** | ***0.26*** | **6.32** | ***0.24*** | **6.41** | ***0.45*** |
| TTYH1 | CD34 | 5.56 | *0.68* | 5.54 | *0.72* | 6.01 | *0.23* | 5.63 | *0.75* |
|  | **GRA** | **5.71** | ***1.26*** | **5.75** | ***0.47*** | **6.02** | ***0.73*** | **4.79** | ***1.23*** |
| TYMP | CD34 | 4.06 | *0.24* | 3.16 | *0.82* | 3.60 |  | 3.60 | *0.39* |
| UBE2Q1 | **GRA** | **4.28** | ***0.47*** |  |  | **2.93** |  |  |  |
| UBXN1 | CD34 | 5.03 | *0.58* | 5.01 | *0.67* | 5.44 | *0.12* | 5.00 | *0.61* |
|  | **GRA** | **5.21** | ***0.83*** | **5.06** | ***0.60*** | **5.00** | ***0.27*** | **4.61** | ***0.68*** |
| UNC13C | CD34 | 5.14 | *0.83* | 4.82 | *0.16* | 5.91 | *0.29* | 3.50 | *2.28* |
| USP6NL | CD34 | 3.26 | *0.91* | 4.08 | *0.81* | 4.81 | *0.02* | 4.44 | *0.04* |
| UTP23 | CD34 | 3.56 | *1.25* | 4.21 | *1.20* | 5.04 | *0.44* | 4.82 | *0.11* |
|  | **GRA** | **5.14** | ***1.59*** | **4.25** | ***0.75*** | **2.82** | ***1.52*** | **5.68** |  |
| XKR9 | **GRA** | **4.50** | ***0.35*** | **4.68** | ***0.36*** | **3.69** | ***1.45*** | **4.15** | ***0.69*** |
| ZNF732 | **GRA** | **3.71** | ***0.15*** | **4.48** | ***0.08*** | **3.08** | ***0.99*** | **4.09** | ***0.39*** |
| ZNF91 | **GRA** | **4.11** | ***0.31*** | **3.90** | ***0.33*** | **3.15** | ***0.89*** | **3.66** | ***0.86*** |

Bolded values correspond to granulocytes (GRA), the rest to CD34^+^ cells
