# Supplementary material for: Microarray and Proteomic Analyses of Myeloproliferative Neoplasms with a Highlight on the mTOR Signaling Pathway
Source: PLoS One. 2015 Aug 14;10(8):e0135463. doi: 10.1371/journal.pone.0135463 (PMC4537205; doi:10.1371/journal.pone.0135463)
Supplement: S3 Table — (DOCX) [file pone.0135463.s003.docx]

**S3 Table.** Genes downregulated more than 3 fold at least in one of MPN subtypes in CD34^+^ cells and granulocytes determined by microarray analyses.

| **Genes** | **Cells** | **PV** | | **ET** | | **PMF** | | **Mut0** | |
| --- | --- | --- | --- | --- | --- | --- | --- | --- | --- |
|  |  | **Mean** | SD | **Mean** | SD | **Mean** | SD | **Mean** | SD |
| ACTB | **GRA** | **-2.87** | ***0.40*** | **-2.30** | ***0.29*** | **-1.22** | ***1.82*** | **-3.41** | ***0.28*** |
| ACTG1 | **GRA** | **-3.20** | ***0.11*** | **-2.19** | ***0.34*** | **-1.19** | ***1.52*** | **-2.83** | ***0.35*** |
| ACTN3 | CD34 | -3.43 | *0.51* | -4.04 | *0.44* | -3.16 | *0.91* | -3.54 | *0.13* |
|  | **GRA** | **-1.90** | ***0.02*** | **-2.58** | ***0.12*** | **-3.16** | ***0.91*** | **-1.91** | ***0.00*** |
| ACVR2B | CD34 | -3.31 | *0.44* | -3.84 | *0.56* | -3.79 | *0.16* | -3.74 | *0.91* |
| AGAP2 | CD34 | -3.36 | *0.30* | -3.84 | *0.40* | -3.71 | *0.66* | -3.71 | *0.66* |
| ALB | CD34 | -4.12 | *0.09* | -4.53 | *0.06* | -4.23 | *0.22* | -4.23 | *0.22* |
| APBA3 | CD34 | -2.62 | *0.58* | -3.01 | *0.37* | -2.67 | *0.77* | -3.16 | *0.90* |
| APOA2 | CD34 | -3.38 | *0.58* | -3.83 | *0.47* | -3.06 | *0.95* | -3.10 | *0.32* |
|  | **GRA** | **-1.62** | ***0.15*** | **-1.95** | ***0.21*** | **-3.06** | ***0.95*** | **-1.81** | ***0.41*** |
| APOB | CD34 | -4.08 |  | -3.71 | *0.16* | -3.10 | *0.58* | -3.28 |  |
|  | **GRA** | **-1.83** | ***0.23*** | **-2.16** | ***0.14*** | **-3.10** | ***0.58*** | **-1.44** |  |
| ATG9B | CD34 | -2.58 | *0.01* | -3.13 | *0.31* | -2.96 | *1.10* | -3.38 | *1.40* |
| ATN1 | CD34 | -2.92 | *0.34* | -3.31 | *0.39* | -3.14 | *0.58* | -3.14 | *0.58* |
| C19orf55 | CD34 | -4.00 | *0.30* | -4.14 | *0.30* | -3.14 | *1.07* | -4.18 | *0.59* |
|  | **GRA** | **-2.08** |  |  |  | **-3.14** | ***1.07*** | **-1.90** | ***0.02*** |
| CAMKK1 | CD34 | -3.39 | *0.60* | -4.10 | *0.42* | -3.53 | *0.33* | -3.53 | *0.33* |
| CASP14L | CD34 | -3.51 | *0.57* | -3.82 | *0.26* | -3.18 | *1.05* | -4.03 | *0.86* |
|  | **GRA** | **-2.07** | ***0.20*** | **-1.96** | ***0.07*** | **-3.18** | ***1.05*** | **-1.68** | ***0.24*** |
| CCT6P1 | CD34 | -3.56 | *0.90* | -3.78 | *0.43* | -3.25 | *0.10* | -3.68 | *0.57* |
| CDC42EP1 | CD34 | -3.17 | *0.28* | -3.46 | *0.29* | -3.67 | *0.10* | -3.37 | *0.71* |
| CELF3 | CD34 | -3.95 | *1.13* | -4.06 | *0.55* | -3.95 | *0.83* | -3.87 | *1.23* |
|  | **GRA** | **-3.01** | ***0.68*** | **-2.82** | ***0.49*** | **-3.95** | ***0.83*** | **-2.66** | ***0.37*** |
| COMMD5 | CD34 | -2.87 | *0.08* | -3.14 | *0.38* | -2.63 | *0.71* | -3.88 |  |
| CSRNP3 | CD34 | -4.12 | *0.57* | -4.33 | *0.38* | -3.69 | *0.86* | -3.98 | *0.64* |
|  | **GRA** | **-2.67** | ***0.12*** | **-2.73** | ***0.34*** | **-3.69** | ***0.86*** | **-2.41** | ***0.21*** |
| CYP2A6 | CD34 | -2.90 | *0.51* | -3.29 | *0.42* | -3.11 | *0.85* | -3.06 | *0.59* |
| DBX1 | CD34 | -3.56 | *0.54* | -4.06 | *0.21* | -3.15 | *0.93* | -3.65 | *0.16* |
|  | **GRA** | **-2.16** | ***0.21*** | **-2.33** | ***0.23*** | **-3.15** | ***0.93*** | **-2.08** | ***0.17*** |
| DHX9 | CD34 | -3.68 | *0.45* | -3.83 | *0.31* | -3.39 | *0.68* | -3.81 | *0.36* |
|  | **GRA** | **-2.51** | ***0.33*** | **-2.65** | ***0.06*** | **-3.39** | ***0.68*** | **-2.13** | ***0.33*** |
| DNAI2 | CD34 | -2.69 | *0.15* | -3.16 | *0.39* | -3.35 | *1.04* | -2.72 | *0.82* |
| EEF1G | **GRA** | **-2.99** | ***0.19*** | **-3.07** | ***0.58*** | **-2.28** | ***0.66*** | **-2.95** | ***0.32*** |
| EFCAB4A | CD34 | -4.19 | *0.56* | -4.40 | *0.48* | -3.62 | *1.09* | -4.09 | *0.67* |
|  | **GRA** | **-2.37** | ***0.02*** | **-2.86** | ***0.01*** | **-3.62** | ***1.09*** | **-2.33** | ***0.32*** |
| ESPNP | CD34 | -4.43 | *0.61* | -4.36 | *0.38* | -3.81 | *1.16* | -4.17 | *0.83* |
|  | **GRA** | **-2.58** | ***0.91*** | **-2.77** | ***0.17*** | **-3.81** | ***1.16*** | **-2.74** | ***0.32*** |
| EXOC3L1 | CD34 | -3.07 | *0.61* | -3.41 | *0.41* | -3.73 | *1.32* | -3.31 | *0.58* |
| FAM216A | CD34 | -3.96 | *0.53* | -4.27 | *0.37* | -3.43 | *0.90* | -4.07 | *0.62* |
|  | **GRA** | **-2.73** | ***0.41*** | **-2.68** | ***0.09*** | **-3.43** | ***0.90*** | **-2.42** | ***0.25*** |
| FAM3C | CD34 | -2.81 | *0.37* | -3.10 | *0.37* | -3.16 | *0.22* | -2.57 | *0.54* |
| FCER2 | CD34 | -3.07 | *0.34* | -3.17 | *0.53* | -3.68 | *0.49* | -2.99 |  |
| FN1 | CD34 | -3.96 | *0.67* | -4.17 | *0.09* | -3.53 | *0.75* | -4.31 | *0.14* |
|  | **GRA** | **-2.25** | ***0.10*** | **-2.34** | ***0.56*** | **-3.53** | ***0.75*** | **-2.64** | ***0.41*** |
| FOXD4 | CD34 | -4.45 | *0.69* | -4.66 | *0.41* | -4.07 | *0.63* | -4.24 | *0.63* |
|  | **GRA** | **-2.95** | ***0.10*** | **-3.21** | ***0.05*** | **-4.07** | ***0.63*** | **-2.77** | ***0.17*** |
| FOXD4L4 | CD34 | -3.64 | *0.39* | -4.20 | *0.43* | -3.49 | *0.95* | -3.94 | *0.47* |
|  | **GRA** | **-2.65** | ***0.21*** | **-2.78** |  | **-3.49** | ***0.95*** | **-2.56** |  |
| FTH1P3 | **GRA** | **-3.09** | ***0.40*** | **-3.33** | ***0.19*** | **-2.43** | ***0.90*** | **-3.13** | ***0.38*** |
| FZD9 | CD34 | -2.63 | *0.74* | -3.31 | *0.33* | -3.40 | *1.23* | -2.92 | *0.93* |
| GAGE12F | CD34 | -3.01 | *0.08* | -3.64 | *0.01* | -3.20 | *0.85* | -3.50 | *0.29* |
|  | **GRA** | **-1.68** | ***0.33*** | **-2.28** |  | **-3.20** | ***0.85*** | **-1.26** | ***0.27*** |
| GAPDH | **GRA** | **-2.65** | ***0.43*** | **-2.40** | ***0.12*** | **-2.66** | ***0.43*** | **-3.44** | ***0.60*** |
| GHRH | CD34 | -3.22 | *0.06* | -3.50 | *0.51* | -3.85 | *0.76* | -3.39 |  |
| GOLGA6L1 | CD34 | -3.16 | *0.39* | -4.03 | *0.38* | -4.20 | *0.79* | -3.58 | *0.62* |
| GOLGA6L6 | CD34 | -3.40 | *0.21* | -4.10 | *0.56* | -3.97 | *0.50* | -3.92 | *0.24* |
| HBE1 | CD34 | -4.10 | *0.82* | -4.26 | *0.43* | -3.60 | *0.78* | -3.58 | *0.71* |
|  | **GRA** | **-2.10** | ***0.12*** | **-2.26** | ***0.38*** | **-3.60** | ***0.78*** | **-2.16** | ***0.41*** |
| HBG2 | CD34 | -3.86 | *1.06* | -2.86 | *1.61* | -3.36 | *0.44* | -3.14 | *0.95* |
|  | **GRA** | **-2.77** | ***0.36*** | **-2.94** | ***0.15*** | **-3.36** | ***0.44*** | **-2.91** | ***0.41*** |
| HMGN2P46 | CD34 | -3.14 | *0.23* | -3.08 | *0.29* | -2.71 | *0.02* | -2.94 | *0.27* |
| ID3 | CD34 | -2.69 | *0.44* | -3.09 | *0.14* | -3.12 | *0.16* | -2.33 | *0.86* |
| IGF2 | CD34 | -3.52 | *0.57* | -3.13 | *0.45* | 2.08 | *1.33* | -2.93 | *0.31* |
| IGLC1 | CD34 | -3.92 | *0.70* | -3.76 | *0.55* | -3.37 | *0.81* | -3.34 | *0.63* |
|  | **GRA** | **-2.28** | ***0.38*** | **-2.43** | ***0.27*** | **-3.37** | ***0.81*** | **-2.25** | ***0.41*** |
| IL1RN | CD34 | -3.46 | *0.52* | -3.81 | *0.46* | -4.01 | *0.79* | -3.36 | *0.76* |
| INCENP | CD34 | -3.94 | *0.32* | -4.45 | *0.58* | -4.59 | *0.80* | -4.19 | *0.72* |
| ITGA3 | CD34 | -3.89 | *0.38* | -4.30 | *0.58* | -4.18 | *0.57* | -3.65 | *0.48* |
|  | **GRA** | **-2.33** | ***0.37*** | **-2.76** |  | **-3.09** | ***0.77*** | **-2.11** | ***0.08*** |
| KIRREL3 | CD34 | -2.64 | *0.30* | -3.42 | *0.25* | -2.97 | *1.21* | -2.02 | *0.47* |
| KLHL38 | CD34 | -3.92 | *0.63* | -3.87 | *0.31* | -3.75 | *0.85* | -4.06 | *0.66* |
| KRT18 | CD34 | -4.23 | *0.38* | -4.26 | *0.19* | -3.48 | *0.91* | -4.31 | *0.70* |
|  | **GRA** | **-2.13** | ***0.27*** | **-2.51** | ***0.40*** | **-3.48** | ***0.91*** | **-2.15** | ***0.12*** |
| KRTAP19-6 | CD34 | -3.29 | *0.45* | -3.84 | *0.44* | -3.81 | *0.57* | -3.53 | *0.57* |
| KRTAP20-1 | CD34 | -3.55 | *0.45* | -3.85 | *0.33* | -3.19 | *0.63* | -3.42 | *0.10* |
|  | **GRA** | **-2.53** | ***0.05*** | **-2.81** | ***0.14*** | **-3.19** | ***0.63*** | **-2.19** | ***0.07*** |
| MLL | CD34 | -2.86 | *0.29* | -2.80 | *0.33* | -2.75 | *0.32* | -3.10 | *0.47* |
| MON1B | CD34 | -2.77 | *0.63* | -3.66 | *0.38* | -3.09 | *1.07* | -1.97 | *0.47* |
| MRAP | CD34 | -2.67 | *0.24* | -3.34 | *0.40* | -3.01 | *1.55* | -2.98 | *1.32* |
| MRPL28 | CD34 | -3.38 | *0.37* | -3.84 | *0.52* | -3.53 | *0.95* | -3.47 | *0.70* |
| MSN | CD34 | -3.49 | *0.37* | -4.24 | *0.15* | -3.05 | *0.84* | -3.88 | *0.70* |
|  | **GRA** | **-2.16** | ***0.04*** | **-2.11** | ***0.13*** | **-3.05** | ***0.84*** | **-1.83** | ***0.31*** |
| MT-CYB | **GRA** | **-3.17** | ***0.83*** | **-1.31** | ***1.09*** | **-2.14** | ***0.02*** | **-3.33** | ***0.31*** |
| NDUFB7 | CD34 | -3.22 | *0.60* | -3.62 | *0.14* | -3.37 | *0.28* | -2.90 | *0.28* |
| NOTUM | CD34 | -2.72 | *0.55* | -3.34 | *0.32* | -3.25 | *0.93* | -2.83 | *0.93* |
| PANX2 | CD34 | -4.31 | *0.73* | -3.70 | *0.35* | -3.99 | *0.42* | -4.33 | *1.43* |
|  | **GRA** | **-3.31** | ***0.91*** | **-2.88** | ***0.43*** | **-3.99** | ***0.42*** | **-3.35** | ***0.50*** |
| PDE4A | CD34 | -3.55 | *0.70* | -3.99 | *0.43* | -3.08 | *0.96* | -3.91 | *0.51* |
|  | **GRA** | **-2.03** | ***0.23*** | **-2.32** | ***0.06*** | **-3.08** | ***0.96*** | **-1.91** | ***0.21*** |
| PGF | CD34 | -2.62 | *0.55* | -3.47 | *0.49* | -3.64 | *0.95* | -2.94 | *0.78* |
| PI4KA | CD34 | -3.28 |  | -3.91 | *0.43* | -3.93 | *0.75* | -3.55 | *0.15* |
|  | **GRA** | **-2.01** |  | **-2.49** |  | **-3.38** | ***1.07*** | **-1.90** | ***0.25*** |
| PITPNM1 | CD34 | -2.57 | *0.16* | -3.06 | *0.46* | -3.34 | *1.07* | -2.50 | *0.53* |
| PKM | CD34 | -2.72 | *0.56* | -3.17 | *0.36* | -2.96 | *0.10* | -2.72 | *0.42* |
| PPA2 | **GRA** | **-2.94** | ***0.18*** | **-3.17** |  | **-3.57** | ***0.86*** | **-2.38** |  |
| PPP4R2 | CD34 | -2.28 | *0.63* | -3.32 | *0.33* | -3.22 | *0.54* | -3.37 | *0.13* |
| PRAME | CD34 | -3.04 | *0.46* | -3.39 | *0.32* | -3.01 | *0.21* | -2.97 | *0.42* |
| Q5T7C0_  HUMAN | CD34 | -4.09 | *0.64* | -3.98 | *0.50* | -4.02 | *0.11* | -3.88 | *0.72* |
|  | **GRA** | **-4.20** | ***0.64*** | **-4.49** | ***0.44*** | **-4.00** | ***0.70*** | **-4.47** | ***0.74*** |
| RAB15 | CD34 | -3.85 | *0.49* | -4.36 | *0.41* | -3.66 | *0.75* | -3.99 | *0.72* |
|  | **GRA** | **-2.65** | ***0.23*** | **-2.33** | ***0.38*** | **-3.66** | ***0.75*** | **-2.15** | ***0.29*** |
| RAI1 | CD34 | -3.06 |  | -3.12 | *0.10* | -2.19 |  | -3.06 | *0.04* |
| RHD | CD34 | -2.98 | *0.43* | -2.90 | *0.43* | -3.10 | *0.81* | -3.14 | *0.46* |
| RPH3AL | CD34 | -2.60 | *0.16* | -3.16 | *0.47* | -3.59 | *0.22* | -3.26 | *0.07* |
| RPL13AP6 | CD34 | -4.00 | *0.47* | -3.71 | *0.34* | -3.63 | *0.66* | -3.52 | *0.44* |
|  | **GRA** | **-2.77** | ***0.02*** | **-2.77** | ***0.33*** | **-3.63** | ***0.66*** | **-2.98** | ***0.56*** |
| RPL19 | **GRA** | **-2.62** | ***0.11*** | **-2.44** | ***0.43*** | **-2.34** | ***0.95*** | **-3.05** | ***0.39*** |
| RPL3 | **GRA** | **-2.43** | ***0.13*** | **-2.43** | ***0.28*** | **-1.78** | ***0.93*** | **-3.06** | ***0.37*** |
| RPL36AP40 | CD34 | -3.39 | *0.28* | -3.32 | *0.30* | -3.14 | *0.37* | -3.04 | *0.42* |
|  | **GRA** | **-2.20** | ***0.04*** | **-2.17** | ***0.27*** | **-3.14** | ***0.37*** | **-2.20** | ***0.48*** |
| RPL7A | **GRA** | **-2.87** |  | **-3.01** | ***0.12*** | **-2.28** | ***0.66*** | **-2.76** | ***0.41*** |
| RPLP0 | **GRA** | **-2.72** | ***0.05*** | **-2.92** | ***0.51*** | **-2.36** | ***0.47*** | **-3.15** | ***0.47*** |
| RPLP0P2 | **GRA** | **-2.68** | ***0.08*** | **-2.96** | ***0.41*** | **-2.75** | ***0.33*** | **-3.01** | ***0.45*** |
| RPS18 | **GRA** | **-2.53** | ***0.09*** | **-2.47** | ***0.43*** | **-1.60** | ***1.52*** | **-3.05** | ***0.49*** |
| RPS25 | **GRA** | **-2.38** | ***0.31*** | **-2.57** | ***0.06*** | **-1.90** | ***1.22*** | **-3.05** | ***0.71*** |
| RPS4X | **GRA** | **-2.63** | ***0.31*** | **-2.99** | ***0.19*** | **-1.73** | ***1.53*** | **-3.29** | ***0.51*** |
| RPS6 | **GRA** | **-2.88** | ***0.35*** | **-2.87** | ***0.26*** | **-2.13** | ***0.82*** | **-3.01** | ***0.47*** |
| RPS8 | **GRA** | **-3.04** | ***0.24*** | **-2.77** | ***0.30*** | **-2.01** | ***0.85*** | **-3.22** | ***0.42*** |
| SEMA3B | CD34 | -2.68 | *0.26* | -3.22 | *0.52* | -3.31 | *0.99* | -3.18 | *0.38* |
| SERPINE2 | CD34 | -3.25 | *0.74* | -3.15 | *0.55* | -3.02 | *0.54* | -2.87 | *0.62* |
|  | **GRA** | **-1.51** | ***0.12*** | **-2.00** | ***0.16*** | **-3.02** | ***0.54*** | **-1.66** | ***0.52*** |
| SHOX | CD34 | -2.73 | *0.74* | -3.29 | *0.48* | -3.49 | *1.20* | -3.16 | *0.53* |
| SIGLEC16 | CD34 | -3.29 | *0.34* | -3.43 | *0.57* | -3.10 | *0.50* | -3.15 | *0.10* |
| SLC9B1 | CD34 | -2.90 | *0.36* | -3.42 | *0.30* | -3.18 | *0.80* | -3.20 | *0.20* |
| SNIP1 | CD34 |  |  | -3.17 | *0.24* | -3.20 | *0.64* | -2.61 | *0.25* |
| SPRR1A | CD34 | -3.20 | *0.60* | -3.76 | *0.27* | -3.09 | *0.87* | -3.27 | *0.97* |
|  | **GRA** | **-2.18** | ***0.12*** | **-2.37** | ***0.33*** | **-3.09** | ***0.87*** | **-2.15** | ***0.23*** |
| SSTR4 | CD34 | -3.86 | *0.39* | -4.14 | *0.52* | -4.13 | *0.81* | -3.92 |  |
| TEF | CD34 | -2.67 | *0.55* | -3.23 | *0.43* | -3.57 | *0.87* | -2.77 | *0.72* |
| TM4SF1 | CD34 | -3.19 | *0.37* | -3.18 | *0.27* | -2.73 |  | -2.91 | *0.50* |
| TMSB4Y | CD34 | -2.98 | *0.24* | -3.20 | *0.34* | -3.86 | *0.46* | -3.01 | *0.80* |
| TNFAIP8L1 | CD34 | -3.01 | *0.38* | -3.46 | *0.15* | -3.54 | *0.77* | -3.12 | *0.27* |
| TPI1 | CD34 | -3.25 | *0.58* | -2.98 | *0.77* | -2.49 | *0.22* | -2.50 | *0.45* |
|  | **GRA** | **-1.95** | ***0.01*** | **-1.96** | ***0.34*** | **-3.28** | ***0.11*** | **-3.32** | ***0.42*** |
| TPI1P2 | CD34 | -3.10 | *0.43* | -3.13 | *0.44* | -2.72 | *0.18* | -2.87 | *0.38* |
| TPT1 | **GRA** | **-2.51** | ***0.20*** | **-2.90** | ***0.20*** | **-1.26** | ***1.87*** | **-3.32** | ***0.40*** |
| TRIM41 | CD34 | -2.81 | *0.49* | -3.62 | *0.37* | -3.62 | *1.03* | -3.13 | *0.58* |
| TUBB | CD34 | -3.01 | *0.53* | -2.79 | *0.61* | -2.16 | *0.82* | -2.61 | *0.66* |
| TUBB8 | CD34 | -4.17 | *0.64* | -4.51 | *0.40* | -3.74 | *0.85* | -4.11 | *0.67* |
|  | **GRA** | **-2.72** | ***0.11*** | **-2.74** | ***0.33*** | **-3.74** | ***0.85*** | **-2.60** | ***0.33*** |
| UBC | **GRA** | **-3.23** | ***0.03*** | **-3.16** | ***0.24*** | **-1.88** | ***1.68*** | **-3.16** | ***0.33*** |
| VCY1B | CD34 | -3.43 | *0.09* | -3.95 | *0.42* | -4.00 | *0.64* | -3.30 |  |
| VIM | **GRA** | **-3.12** | ***0.14*** | **-3.09** | ***0.49*** | **-1.59** | ***1.46*** | **-3.22** | ***0.28*** |
| VWA3B | CD34 | -3.25 | *0.87* | -4.01 | *0.31* | -3.73 | *0.24* | -3.86 | *1.22* |
|  | **GRA** | **-2.90** | ***0.76*** | **-2.75** | ***0.26*** | **-3.73** | ***0.24*** | **-2.63** | ***0.35*** |
| YBEY | CD34 | -3.64 | *0.62* | -4.06 | *0.41* | -3.04 | *1.05* | -3.79 | *0.84* |
|  | **GRA** | **-2.24** | ***0.33*** | **-2.09** | ***0.04*** | **-3.04** | ***1.05*** | **-2.03** | ***0.07*** |
| ZBTB12 | CD34 | -4.57 | *0.70* | -4.44 | *0.37* | -3.79 | *0.70* | -4.23 | *0.67* |
|  | **GRA** | **-2.98** | ***0.27*** | **-2.98** | ***0.22*** | **-3.79** | ***0.70*** | **-3.00** | ***0.30*** |
| ZC3H18 | CD34 | -2.98 | *0.32* | -3.52 | *0.29* | -3.55 | *0.91* | -3.27 | *0.75* |
| ZNF124 | CD34 | -2.88 |  | -3.03 | *0.13* | -2.72 | *0.24* | -2.88 | *0.37* |
| ZNF14 | CD34 | -3.52 |  | -3.10 | *0.37* | -3.21 | *0.85* | -2.91 | *0.26* |
| ZNF181 | CD34 | -3.60 | *0.47* | -3.59 | *0.25* | -3.19 | *0.46* | -3.47 | *0.34* |
|  | **GRA** | **-2.25** | ***0.43*** | **-2.57** | ***0.21*** | **-3.19** | ***0.46*** | **-2.60** | ***0.37*** |
| ZNF30 | CD34 | -2.96 | *0.48* | -2.71 | *0.51* | -2.49 | *0.03* | -2.49 | *0.48* |
|  | **GRA** | **-1.90** | ***0.38*** | **-2.48** | ***0.55*** | **-3.09** | ***0.16*** | **-1.90** | ***0.25*** |
| ZNF334 | CD34 | -3.51 | *0.31* | -3.59 | *0.33* | -3.32 | *0.48* | -3.71 | *0.55* |
|  | **GRA** | **-2.17** | ***0.60*** | **-2.38** | ***0.15*** | **-3.32** | ***0.48*** | **-2.15** | ***0.24*** |
| ZNF501 | CD34 | -3.63 | *0.25* | -3.37 | *0.37* | -3.04 | *0.27* | -3.64 | *0.54* |
|  | **GRA** | **-1.37** |  | **-1.95** |  | **-3.12** | ***0.32*** | **-1.80** | ***0.19*** |
| ZNF84 | CD34 | -3.23 | *0.23* | -2.92 | *0.32* | -2.79 | *0.14* | -3.00 | *0.46* |
|  | **GRA** | **-2.20** | ***0.32*** | **-2.37** | ***0.25*** | **-3.02** | ***0.41*** | **-2.10** | ***0.18*** |

Bolded values correspond to granulocytes (GRA), the rest to CD34^+^ cells
