# Supplementary material for: Microarray and Proteomic Analyses of Myeloproliferative Neoplasms with a Highlight on the mTOR Signaling Pathway
Source: PLoS One. 2015 Aug 14;10(8):e0135463. doi: 10.1371/journal.pone.0135463 (PMC4537205; doi:10.1371/journal.pone.0135463)
Supplement: S4 Table — (DOCX) [file pone.0135463.s004.docx]

**S4 Table.** The statistically significant genes (p<0.05) among MPNs in granulocytes determined by microarray analysis.

| **Genes** | **MD** | **BG** | **Group A - ET** | | **Group B - PV** | | **Group C - PMF** | | **Group D -Mut0** | |
| --- | --- | --- | --- | --- | --- | --- | --- | --- | --- | --- |
|  |  |  | **Mean** | **SD** | **Mean** | **SD** | **Mean** | **SD** | **Mean** | **SD** |
| ARHGEF5 | 1.16 | D-C | -0.16 | 0.04 | -0.24 | 0.20 | -1.19 | 0.14 | -0.03 | 1.16 |
| HLA-DPB1 | 0.71 | D-B | -1.32 | 0.20 | -1.42 | 0.03 | -1.24 | 0.17 | -0.71 | 0.71 |
| NBPF15 | 2.75 | B-A | -1.19 | 0.04 | 1.56 | 0.58 | 0.43 | 0.22 | -0.42 | 2.75 |
| 61E3.4 | 0.93 | A-C | -0.83 | 0.05 | -1.30 | 0.11 | -1.76 | 0.16 | -1.24 | 0.93 |
| ITGB7 | 0.98 | B-C | -1.02 | 0.00 | -1.10 | 0.20 | -2.08 | 0.24 | -1.31 | 0.98 |
| S100A9 | 3.61 | C-A | 2.04 | 0.33 | 5.10 | 0.20 | 5.64 | 1.53 | 2.52 | 3.61 |
| APOE | 0.48 | D-B | -0.25 | 0.01 | -0.71 | 0.12 | -1.46 | 0.00 | -0.23 | 0.48 |
| NR3C1 | 0.33 | D-C | 0.69 | 0.00 | 0.77 | 0.00 | -0.05 | 0.04 | 0.28 | 0.33 |
| TF | 1.58 | A-C | -0.97 | 0.04 | -1.57 | 0.07 | -2.55 | 0.46 | -1.11 | 1.58 |
| TSPAN5 | 1.50 | D-B | 1.33 | 0.00 | 0.01 | 0.31 | 0.59 | 0.18 | 1.51 | 1.50 |
| TMEM63B | 0.30 | D-B |  |  | 0.17 | 0.06 | 1.01 | 0.00 | 0.47 | 0.30 |
| PTMA | 0.69 | D-C | -1.96 | 0.20 | -2.08 | 0.08 | -2.21 | 0.03 | -1.52 | 0.69 |
| IL18R1 | 1.27 | D-C | 1.74 | 0.23 | 1.40 | 0.11 | 0.60 | 0.62 | 1.87 | 1.27 |
| ZFP36L1 | 1.05 | D-C | -0.19 | 0.32 | -0.11 | 0.18 | -0.75 | 0.37 | 0.30 | 1.05 |
| HNRNPUL1 | 0.72 | D-C | -0.81 | 0.10 | -0.88 | 0.09 | -1.29 | 0.09 | -0.57 | 0.72 |
| PRPSAP2 | 0.80 | A-B | 3.01 | 0.06 | 2.22 | 0.12 | 2.41 | 0.12 | 2.37 | 0.80 |
| PDHA1 | 0.99 | D-C | 0.30 | 0.00 | 1.83 | 0.00 | -0.82 | 0.07 | 0.17 | 0.99 |
| EIF4H | 0.72 | A-B | 0.10 | 0.05 | -0.63 | 0.12 | -0.42 | 0.12 | -0.32 | 0.72 |
| MYO7B | 0.39 | A-B | 2.61 | 0.02 | 2.22 | 0.08 | -0.01 | 0.00 | 2.06 | 0.39 |
| DEXI | 0.34 | C-B | 0.75 | 0.00 | 0.47 | 0.04 | 0.81 | 0.06 | 0.85 | 0.34 |
| PITPNM1 | 1.33 | B-C | -1.30 | 0.22 | -1.11 | 0.07 | -2.44 | 0.52 | -1.24 | 1.33 |
| TSPYL2 | 0.40 | A-C | 1.17 | 0.01 | 1.36 | 0.00 | 0.76 | 0.16 | 1.14 | 0.40 |
| BLOC1S3 | 1.32 | D-C | 0.67 | 0.00 | 0.68 | 0.00 | -0.84 | 0.13 | 0.47 | 1.32 |
| MIR424 | 3.14 | B-A | 0.38 | 0.29 | 3.52 | 0.82 | 2.72 | 0.75 | 1.09 | 3.14 |
| RBL2 | 0.84 | A-D | 3.32 | 0.37 | 3.11 | 0.31 | 2.67 | 0.06 | 2.48 | 0.84 |
| FAM127A | 1.06 | A-C | 0.54 | 0.22 | 0.12 | 0.10 | -0.51 | 0.28 | 0.25 | 1.06 |

The negative values represent downregulated genes, while positive values represent upregulated genes compared to HuURNA. BG – Between groups significance with p<0.05, MD – Maximal mean difference of corresponding BG.
