# Supplementary material for: Microarray and Proteomic Analyses of Myeloproliferative Neoplasms with a Highlight on the mTOR Signaling Pathway
Source: PLoS One. 2015 Aug 14;10(8):e0135463. doi: 10.1371/journal.pone.0135463 (PMC4537205; doi:10.1371/journal.pone.0135463)
Supplement: S5 Table — (DOCX) [file pone.0135463.s005.docx]

**S5 Table.** Comparison of statistically significant protein expression in granulocytes, determined by proteomic analysis, with their counterpart gene expression determined by microarray analysis in CD34^+^ cells of MPNs.

|  | **Proteomic** - ratio | | | | | | **Microarray -** mean difference | | | | | |
| --- | --- | --- | --- | --- | --- | --- | --- | --- | --- | --- | --- | --- |
| **Genes** | PV/  ET | PV/  PMF | ET/  PMF | PV/Mut0 | ET/ Mut0 | PMF/ Mut0 | PV / ET | PV / PMF | ET / PMF | PV / Mut0 | ET / Mut0 | PMF/Mut0 |
| ACAT2 |  |  |  |  |  | 1.14 |  |  |  | -0.19 |  |  |
| ACSL1 | 1.22 | 1.11 |  | 1.15 |  |  | 0.59 | -0.46 | -1.05 | 0.87 | 0.28 | 1.33 |
| ACTG |  | 0.72 | 0.72 |  |  |  | 0.44 | 0.003 | -0.44 | -0.37 | -0.82 | -0.38 |
| ACTN1 | 0.91 |  | 1.10 |  | 1.16 |  | -0.12 | -0.36 | -0.24 | 0.01 | 0.12 | 0.37 |
| ACTN4 |  |  |  |  |  | 1.15 |  |  |  |  |  |  |
| ACTR2 | 1.06 |  |  | 0.94 | 0.91 | 0.94 | 0.61 | 0.38 | -0.23 | 0.45 | -0.16 | 0.06 |
| ACTR3 | 1.10 |  | 0.93 |  | 0.92 |  | 0.61 | 0.60 | -0.01 | 0.77 | 0.16 | 0.17 |
| AGL |  |  |  |  |  | 1.07 |  |  |  |  |  |  |
| AKR1B1 | 0.81 |  |  |  |  | 1.08 | -0.02 | -1.16 | -1.15 | 0.03 | 0.04 | 1.19 |
| ALB | 0.90 |  | 1.17 |  | 1.11 | 0.96 | -0.41 | -0.39 | 0.02 | -0.31 | 0.10 | 0.08 |
| ALDOA |  |  | 0.97 |  |  |  | -0.08 | -0.22 | -0.13 | 0.17 | 0.25 | 0.39 |
| ALOX5 |  |  |  |  | 0.88 | 1.39 |  |  |  |  |  |  |
| ALOX5AP | 1.04 | 0.97 | 1.12 |  |  | 0.83 | -0.60 |  |  |  |  |  |
| ANXA1 | 0.95 | 0.97 | 1.05 |  | 1.09 | 1.06 | 0.67 | 0.70 | 0.02 | 0.75 | 0.07 | 0.05 |
| ANXA2 |  |  |  | 0.77 |  |  | 0.54 | 1.37 | 0.83 | 0.60 | 0.06 | -0.77 |
| ANXA3 | 0.93 |  |  | 1.04 | 1.13 | 1.06 |  |  |  |  |  |  |
| ANXA4 |  | 1.11 | 1.09 |  |  | 0.95 | 0.43 | 0.33 | -0.10 | 0.27 | -0.16 | -0.06 |
| ANXA5 | 0.88 | 0.83 | 0.95 |  | 1.16 | 1.22 | -0.23 | 0.89 | 1.12 | 0.27 | 0.50 | -0.62 |
| ANXA6 |  |  |  |  |  | 0.98 | -0.86 |  |  | -0.04 | 0.82 |  |
| AP1M1 | 1.15 |  |  |  |  |  |  |  |  |  |  |  |
| APEX1 |  |  |  |  |  | 0.96 | 1.08 | -0.33 | -1.42 | 0.47 | -0.62 | 0.80 |
| APOB48R |  |  |  |  | 1.12 | 1.08 |  |  |  |  |  |  |
| ARC1B |  |  |  | 0.87 | 0.80 |  |  |  |  |  |  |  |
| ARG1 | 0.95 |  |  |  |  |  |  |  |  |  |  |  |
| ARHGDIA |  |  |  |  |  | 0.95 |  |  |  |  |  |  |
| ARHGDIB | 0.91 | 0.94 |  | 0.86 | 0.93 | 0.93 | -0.10 | -0.04 | 0.07 | 0.29 | 0.39 | 0.33 |
| ARPC1B |  | 0.86 | 0.80 |  |  |  | 0.24 | 0.08 | -0.16 | -0.23 | -0.47 | -0.31 |
| ARPC2 |  |  | 0.92 |  | 0.89 |  | 0.06 | 0.33 | 0.27 | -0.09 | -0.15 | -0.42 |
| ARPC3 |  |  | 0.87 |  |  |  | 0.17 | 0.66 | 0.49 | 0.13 | -0.04 | -0.54 |
| ARPC5 |  |  |  |  |  | 0.96 | 0.39 | 0.61 | 0.22 | 0.48 | 0.10 | -0.13 |
| ASL |  |  |  |  |  | 0.97 |  |  |  |  |  |  |
| ASRGL1 | 1.07 |  |  |  |  |  |  |  |  |  |  |  |
| ATG7 |  |  |  |  |  | 1.13 |  |  |  |  |  |  |
| ATP5A1 |  |  |  |  | 0.92 |  | 0.53 | -0.14 | -0.67 | 0.11 | -0.42 | 0.25 |
| ATP5B | 1.08 |  |  |  | 0.92 | 0.96 | 0.51 | -0.11 | -0.62 | 0.07 | -0.44 | 0.18 |
| ATP6V1E1 | 1.11 |  |  |  |  |  | -0.22 | -0.29 | -0.07 | 0.24 | 0.46 | 0.53 |
| AZU1 | 0.88 | 1.19 | 1.41 |  |  |  |  |  |  |  |  |  |
| BLVRB | 0.91 | 1.09 | 1.21 | 1.18 | 1.31 | 1.09 | -0.13 | 0.15 | 0.28 | -0.20 | -0.08 | -0.36 |
| BPI | 1.09 | 0.90 | 0.87 | 0.84 | 0.81 | 0.94 |  |  |  |  |  |  |
| BST1 |  |  | 1.21 |  | 1.13 |  | 1.17 | -0.05 | -1.22 | 1.18 | 0.01 | 1.23 |
| C9orf19 | 0.90 | 1.02 |  |  | 1.15 | 1.09 |  |  |  |  |  |  |
| CA1 | 0.83 | 1.20 | 1.44 | 1.22 | 1.47 |  |  | 2.82 |  |  |  |  |
| CA2 | 0.79 |  | 1.27 |  | 1.28 |  | -1.49 |  |  |  |  |  |
| CAB39L |  |  |  |  |  | 1.07 |  |  |  |  |  |  |
| CALM2 |  | 0.80 | 0.76 |  | 0.88 | 1.22 | 0.41 | 0.57 | 0.15 | 0.23 | -0.18 | -0.34 |
| CALR |  |  |  | 1.29 | 1.28 | 1.22 |  |  |  |  |  | -0.41 |
| CAMP | 0.92 | 1.15 | 1.28 | 1.08 | 1.20 | 0.94 |  |  |  |  |  |  |
| CAND1 | 1.12 |  |  |  |  |  | 0.14 | -0.73 | -0.87 | 0.43 | 0.29 | 1.16 |
| CANX | 1.08 |  |  |  | 0.94 |  | 0.93 | 0.40 | -0.53 | 0.79 | -0.14 | 0.39 |
| CAP1 |  |  |  | 0.84 |  |  | -0.41 | -0.09 | 0.32 | -0.35 | 0.06 | -0.26 |
| CAP7 |  |  |  | 0.77 | 0.88 | 0.65 |  |  |  |  |  |  |
| CAPG |  |  |  |  |  | 0.96 | 0.46 |  |  | 0.98 | 0.52 |  |
| CAPN1 | 1.25 |  |  |  | 0.91 | 0.96 |  |  |  |  |  |  |
| CAPZA1 |  |  | 0.89 | 0.83 | 0.80 | 0.90 | 0.06 | -0.83 | -0.89 | 0.24 | 0.18 | 1.07 |
| CAPZA2 |  |  | 0.78 |  | 0.87 |  | 0.08 | -0.33 | -0.41 | 0.14 | 0.06 | 0.47 |
| CAT | 0.93 |  |  | 0.90 | 0.97 | 0.94 | 0.75 | 1.05 | 0.29 | -0.03 | -0.78 | -1.07 |
| CBX3 |  |  |  |  | 0.88 |  | 0.73 | -0.46 | -0.67 | 0.65 | -0.08 | 0.58 |
| CCT2 | 1.12 |  | 0.84 |  | 0.86 |  | 1.07 | -0.58 | -1.65 | 0.11 | -0.96 | 0.69 |
| CCT3 | 1.11 |  |  |  |  |  | 0.35 | -0.80 | -1.15 | 0.42 | 0.07 | 1.22 |
| CCT5 | 1.22 |  | 0.87 |  | 0.89 |  | 0.03 | -0.48 | -0.51 | 0.45 | 0.42 | 0.93 |
| CD177 |  | 1.69 | 1.52 |  |  |  |  |  |  |  |  |  |
| CD44 | 1.21 |  |  | 1.30 |  |  | -0.27 | 0.34 | 0.60 | -0.39 | -0.12 | -0.72 |
| CD59 | 0.83 |  |  |  | 1.17 |  |  | -0.90 |  | -0.62 |  | 0.28 |
| CFL1 | 0.95 | 0.85 | 0.92 | 0.90 | 0.94 |  | 0.12 | 0.19 | 0.07 | -0.12 | -0.24 | -0.31 |
| CHI3L1 | 1.10 | 1.18 | 1.10 | 1.14 |  | 0.96 |  |  | 0.32 |  |  |  |
| CHIT1 | 0.82 |  | 1.21 |  |  | 0.89 | -0.36 | -1.10 | -0.74 | -0.36 | 0.00 | 0.74 |
| CHMP5 | 2.24 |  |  |  |  |  | 0.43 | 0.36 | -0.06 | 0.65 | 0.22 | 0.29 |
| CLIC1 |  |  | 0.92 |  |  | 1.12 | 0.14 | -0.33 | -0.26 | -0.57 | -0.07 | -0.23 |
| CLTC | 1.23 |  | 0.88 |  |  |  | 0.47 | -0.49 | -0.96 | 0.27 | -0.20 | 0.76 |
| CORO1A | 0.92 | 0.97 | 1.06 | 0.97 |  |  | 0.13 | 0.58 | 0.45 | 0.15 | 0.02 | -0.43 |
| COTL1 | 0.86 | 0.84 |  | 0.84 |  |  | -0.19 | 0.71 | 0.90 | -0.47 | -0.29 | -1.18 |
| CPNE2 | 1.31 |  |  |  |  |  |  |  |  |  |  |  |
| CPNE3 |  |  | 0.94 |  |  | 1.08 | -0.41 | -1.20 | -0.79 | -1.25 | -0.84 | -0.05 |
| CSK |  |  | 0.84 |  |  |  | 0.08 | 0.61 | 0.53 | 0.23 | 0.15 | -0.38 |
| CTSD | 1.28 |  | 0.80 | 1.13 | 0.89 | 1.13 | -0.16 | 0.08 | 0.24 | -0.28 | -0.13 | -0.37 |
| CTSG | 1.25 | 1.36 | 1.11 |  | 0.81 | 0.74 |  |  |  |  |  |  |
| CTSS |  |  |  |  |  | 0.89 | 0.33 | 0.81 | 0.48 | 0.42 | 0.09 | -0.39 |
| CYTB |  |  |  |  |  | 1.32 |  |  |  |  |  |  |
| DDT | 1.01 |  |  |  | 1.00 | 1.07 | 0.62 | 0.31 | -0.31 | -0.07 | -0.68 | -0.37 |
| DEK |  |  |  |  |  | 1.14 | 0.54 | -0.23 | -0.77 | 0.44 | -0.09 | 0.67 |
| DLST |  |  |  |  |  | 0.91 |  |  |  | 0.37 |  |  |
| DPP3 |  |  |  |  |  | 1.16 | -0.05 | -0.63 | -0.58 | 0.00 | 0.05 | 0.64 |
| DRIP4 |  |  |  |  |  | 0.88 |  |  |  |  |  |  |
| DYNC1H1 | 1.10 |  |  |  |  |  | 0.17 | 0.15 | -0.03 | 0.64 | 0.47 | 0.49 |
| EEF1D |  |  |  |  | 0.95 |  | -0.31 | -0.29 | 0.02 | -0.35 | -0.04 | -0.06 |
| EFHD2 |  |  |  | 1.10 |  |  | 0.32 |  |  | 0.27 | -0.05 |  |
| EHD1 |  |  |  |  |  | 0.93 |  |  |  |  |  |  |
| EIF2S1 |  | 1.08 |  |  |  |  |  |  |  | 0.05 |  |  |
| ELANE |  | 1.15 |  | 0.93 |  | 0.81 |  |  |  |  |  |  |
| EML4 | 2.02 |  |  |  |  |  | 0.54 |  |  | 0.51 |  |  |
| ENO1 | 0.95 |  |  |  |  |  | 0.15 | -0.84 | -0.94 | -0.43 | -0.58 | 0.36 |
| ENO3 |  | 0.90 |  | 0.89 |  |  |  |  |  |  |  |  |
| EPX | 1.25 |  | 0.88 |  | 0.83 | 0.95 |  |  |  |  |  |  |
| EVI2B |  |  |  | 0.79 | 0.81 |  | 0.21 | 0.56 | 0.36 | 1.09 | 0.89 | 0.53 |
| FAM129A | 1.23 | 1.17 |  | 1.34 |  | 1.15 |  | -1.27 |  | -0.75 |  | 0.52 |
| FAM189B | 0.66 |  | 1.60 |  | 1.60 |  |  |  |  |  |  |  |
| FBP1 |  |  |  |  |  | 1.20 | 0.00 |  |  | -0.47 |  |  |
| FCGR3B |  |  |  |  | 1.41 | 1.38 |  |  |  |  |  |  |
| FCN1 | 1.39 |  | 0.74 |  | 0.77 |  | -0.40 | 2.47 | 2.88 | 0.50 | 0.91 | -1.97 |
| FERMT3 | 1.27 |  |  |  | 0.92 |  |  |  |  |  |  |  |
| FLNA |  |  |  | 0.94 | 0.94 | 0.94 | -0.39 | -0.45 | -0.06 | -1.10 |  | -0.66 |
| FNBP1 |  |  |  |  |  | 1.08 | -0.33 | 0.31 | 0.64 | -0.80 | -0.47 | -1.11 |
| FOLR3 | 2.85 | 1.61 | 0.57 |  | 0.36 | 0.63 | 2.90 |  |  | 0.17 |  |  |
| G6PD | 1.17 |  | 0.91 |  |  |  |  |  | 0.63 |  |  |  |
| GAPDH |  |  | 0.98 | 0.94 | 0.94 | 0.98 | 0.16 | -0.67 | -0.55 | -0.39 | -0.58 | -0.03 |
| GDI1 |  |  | 1.09 |  |  |  |  |  |  |  |  |  |
| GDI2 |  |  |  | 0.92 |  | 0.94 | 0.57 | -0.17 | -0.74 | 0.57 | 0.001 | 0.74 |
| GLRX |  | 0.91 | 0.89 |  |  | 1.08 | 0.34 | 1.09 | 0.75 | 1.04 | 0.70 | -0.05 |
| GMFG |  |  |  |  |  | 0.93 | 0.09 | -0.12 | -0.21 | -0.12 | -0.22 | -0.01 |
| GNA13 | 0.92 |  |  |  |  |  | 0.25 | 0.48 | 0.23 | 0.22 | -0.03 | -0.26 |
| GNAI2 | 0.92 |  |  |  |  |  | -0.66 | 0.72 | 0.64 | -0.99 | -0.32 | -0.97 |
| GPI | 1.18 | 0.81 | 0.73 | 0.85 | 0.76 |  | 0.07 | -0.51 | -0.58 | -0.51 | -0.58 | -0.01 |
| GSN | 0.95 |  |  | 0.96 |  |  |  |  |  |  |  |  |
| GSR |  |  |  |  |  | 1.04 | 0.35 |  |  | -0.23 |  |  |
| GSTO1 | 1.07 | 1.17 | 1.10 | 1.09 |  | 0.93 | -0.11 | -0.27 | -0.15 | -0.18 | -0.06 | 0.09 |
| GSTP1 | 0.90 | 1.00 |  |  | 0.94 | 0.91 | -0.15 | -0.36 | -0.20 | -0.57 | -0.41 | -0.21 |
| GYG1 | 1.15 |  | 0.90 |  |  | 1.21 | 0.36 |  |  | -0.40 | -0.76 |  |
| H12 | 0.90 |  |  |  |  |  |  |  |  |  |  |  |
| H15 | 0.89 | 0.92 |  |  | 1.08 |  |  |  |  |  |  |  |
| H2AFY | 0.90 |  |  |  |  |  | 0.22 | -0.73 | -0.95 | -0.47 | -0.70 | 0.26 |
| HBA2 | 0.74 |  | 1.40 |  | 1.41 |  |  |  |  |  |  |  |
| HBB | 0.76 | 1.03 | 1.37 |  | 1.44 | 1.06 | 1.20 | 0.46 | -0.74 | 0.52 | -0.68 | 0.06 |
| HBB | 0.62 |  | 2.17 |  | 1.95 |  |  |  |  |  |  |  |
| HBB | 0.52 |  | 2.23 | 1.11 | 2.18 |  |  |  |  |  |  |  |
| HBD | 0.63 | 1.25 | 1.82 |  | 1.61 | 0.90 | 3.03 | 3.82 | 0.78 | 3.15 | 0.11 | -0.67 |
| HBG1 |  |  | 1.61 |  |  | 0.61 |  |  |  |  |  |  |
| HIST1H2BO |  |  |  | 0.85 |  | 0.84 | -0.60 | -0.47 | 0.13 | -0.35 | 0.25 | 0.12 |
| HK3 | 1.25 | 1.09 | 0.91 | 1.12 | 0.90 |  | 0.95 |  |  | 1.30 | 0.35 |  |
| HNRNPD |  |  |  |  |  | 1.08 | 0.42 | 0.08 | -0.34 | 0.24 | -0.18 | 0.16 |
| HP |  | 1.13 | 1.12 | 1.17 | 1.14 |  | 0.27 | 0.30 | 0.03 | 0.01 | -0.26 | -0.29 |
| HSP90AA1 |  |  |  |  | 0.92 |  | 0.50 | -0.44 | -0.94 | 0.30 | -0.19 | 0.75 |
| HSPA8 |  |  |  |  |  | 0.98 | 0.75 | 0.27 | -0.48 | 0.76 | -0.27 | 0.21 |
| HSP90AB1 | 1.08 |  |  |  | 0.93 | 0.96 | 0.78 | -0.41 | -1.20 | 0.38 | -0.40 | 0.80 |
| HSPA1A | 0.84 | 1.15 | 1.39 | 1.13 | 1.35 |  | 0.66 | 0.66 | 0.00 | 1.17 | 0.51 | 0.51 |
| HSPA5 | 1.13 | 1.13 |  | 1.22 | 1.09 | 1.08 |  |  |  |  |  |  |
| HSPA6 |  |  |  | 1.14 |  |  |  |  |  |  |  |  |
| ICAM3 | 0.87 |  | 1.17 |  | 1.13 |  | 0.16 | -0.32 | -0.47 | -0.35 | -0.51 | -0.04 |
| IFI30 |  |  | 0.82 |  | 1.15 | 1.41 | 0.12 | 0.41 | 0.29 | 0.27 | 0.15 | -0.14 |
| IFI35 | 1.32 |  |  |  |  |  | 0.19 | 0.49 | 0.30 | 0.24 | 0.05 | -0.25 |
| IQGAP1 |  | 0.93 | 0.95 | 0.96 |  |  | 0.12 | 1.07 | 0.95 | 0.23 | 0.11 | -0.84 |
| ITGA2B | 0.68 |  |  |  |  | 0.71 | -0.67 | -0.36 | 0.31 | -1.53 | -0.86 | -1.17 |
| ITGAM | 1.68 | 1.16 | 1.31 |  | 1.15 | 0.90 |  |  |  |  |  |  |
| ITGB2 |  |  | 1.07 |  | 1.06 |  |  |  |  |  |  |  |
| ITGB5 |  |  | 1.17 |  |  | 0.91 |  |  |  |  |  |  |
| KPNB1 | 1.10 |  | 0.91 |  | 0.91 |  | 0.36 | -0.24 | -0.60 | 0.11 | -0.25 | 0.35 |
| KRT1 | 1.41 | 1.30 |  | 0.59 | 0.45 | 0.49 |  |  |  |  |  |  |
| KRT10 | 1.45 | 1.38 |  | 0.70 | 0.55 | 0.56 | 0.28 | -0.08 | -0.35 | -0.29 | -0.57 | -0.22 |
| KRT2 | 1.83 | 1.83 |  |  | 0.59 | 0.60 |  |  |  |  |  |  |
| KRT9 | 1.51 | 1.45 |  | 0.72 | 0.49 | 0.55 |  |  |  |  |  |  |
| LCN2 | 0.98 |  |  |  |  |  |  |  |  |  |  |  |
| LCP1 | 1.05 | 0.98 | 0.96 |  | 0.96 |  | 0.23 | 0.33 | 0.10 | 0.30 | 0.07 | -0.03 |
| LDHAL6B |  |  | 0.88 |  |  | 1.17 |  |  |  |  |  |  |
| LDHB |  |  |  | 0.90 |  | 0.85 | 0.95 | -0.73 | -1.68 | -0.22 | -1.17 | 0.51 |
| LGALS1 | 0.83 | 0.64 | 0.77 | 0.80 |  | 1.30 | -0.33 | 1.00 | 1.33 | -0.72 | -0.39 | -1.72 |
| LGALS10A |  | 0.64 | 0.66 | 0.77 | 0.78 | 1.22 |  |  |  |  |  |  |
| LMNB1 | 1.10 |  |  |  | 0.93 |  |  |  |  |  |  |  |
| LTA4H | 0.95 |  |  | 0.94 |  | 0.94 | 0.56 | 0.57 | 0.01 | 0.81 | 0.25 | 0.24 |
| LXN | 1.45 |  |  |  |  |  | 0.40 | -0.45 | -0.85 | 0.54 | 0.14 | 0.99 |
| LYZ | 0.92 | 1.08 | 1.22 |  | 1.10 | 0.91 | 0.23 | 2.04 | 1.81 | 0.09 | -0.15 | -1.96 |
| MARCKS | 1.31 | 1.19 |  | 1.32 |  | 1.11 |  |  | -0.20 | -0.05 |  |  |
| MCEMP1 | 1.14 | 1.11 |  | 1.33 |  | 1.20 |  |  |  |  |  |  |
| MCTS1 |  | 0.87 |  |  |  |  |  |  |  |  |  |  |
| MDH1 |  |  |  |  |  | 0.95 | -0.08 | 0.11 | 0.19 | -0.01 | 0.07 | -0.12 |
| ME2 | 0.93 |  |  |  |  |  | 0.35 | 0.53 | 0.19 | 1.07 | 0.72 | 0.53 |
| MIF | 0.89 |  |  |  |  |  | 0.17 | -0.74 | -0.91 | -0.65 | -0.82 | 0.09 |
| MMP8 | 1.11 | 1.13 |  | 1.24 | 1.13 | 1.10 |  |  |  |  |  |  |
| MMP9 |  | 1.10 | 1.11 | 1.26 | 1.27 | 1.14 |  |  |  |  |  |  |
| MNDA | 0.94 |  |  | 0.93 |  |  | 1.09 |  |  |  |  |  |
| MPO |  | 1.21 | 1.22 | 0.94 | 0.96 | 0.80 |  | -2.96 |  |  |  |  |
| MSN | 0.92 |  |  |  | 1.06 |  | -0.74 | -0.12 | 0.63 | -0.35 | 0.39 | -0.24 |
| MVP | 1.20 |  |  | 1.38 |  | 1.22 | -0.19 | 0.82 | 1.00 | 0.15 | 0.34 | -0.66 |
| MYH9 | 0.87 | 0.90 | 1.06 | 0.87 |  | 0.96 |  |  |  |  |  |  |
| MYL6 | 0.86 |  |  |  |  |  | -0.51 | 0.51 | 1.02 | -0.49 | 0.02 | -1.00 |
| MYO1F |  |  |  |  | 0.93 |  | 0.08 |  |  | 0.23 | 0.16 |  |
| NAMPT |  |  |  | 1.08 |  | 1.11 | -0.02 | 0.14 | 0.16 | 0.16 | 0.18 | 0.02 |
| NAPRT1 |  |  |  | 1.12 |  | 1.13 |  |  |  |  |  |  |
| NDRG1 | 1.17 | 0.71 | 0.66 |  |  | 0.88 | -0.40 | -0.09 | 0.32 | -0.15 | 0.26 | -0.06 |
| NME1 |  |  |  |  |  | 1.14 | 0.22 | -0.89 | -1.12 | -0.12 | -0.34 | 0.77 |
| NPC2 |  | 0.84 | 0.80 | 0.84 | 0.79 |  | 0.02 | 0.65 | 0.63 | 0.26 | 0.24 | -0.39 |
| NQO2 |  |  |  |  | 1.23 |  | -0.62 |  |  |  |  |  |
| NSF |  |  |  |  |  | 0.92 | -0.05 | 0.56 | 0.61 | 0.33 | 0.37 | -0.24 |
| OLFM4 | 1.21 | 0.85 | 0.72 | 1.21 |  | 1.43 |  |  |  |  |  |  |
| ORM1 | 0.85 |  | 1.18 |  |  |  | 0.49 |  |  | -0.32 |  |  |
| ORM2 |  |  |  |  | 1.25 |  |  |  |  |  |  |  |
| OSTF1 | 0.90 | 0.87 |  | 0.85 |  |  | -0.15 | 0.36 | 0.51 | 0.04 | 0.19 | -0.32 |
| P4HB |  |  |  |  | 0.99 |  | 0.21 | -0.20 | -0.41 | -0.24 | -0.45 | -0.03 |
| PA2G4 |  |  |  |  |  | 0.96 | 0.14 | -0.53 | -0.67 | -0.79 | -0.93 | -0.26 |
| PAICS |  |  |  |  |  | 1.08 |  | -1.16 |  | -0.18 |  | 0.98 |
| PARK7 | 0.93 |  |  |  |  | 0.94 | -0.10 | -0.22 | -0.12 | -0.22 | -0.12 | 0.00 |
| PCBP1 | 1.21 |  |  |  |  |  | 0.37 | 0.45 | 0.08 | 0.43 | 0.06 | -0.02 |
| PDIA4 | 1.21 |  |  |  |  |  |  | 0.42 |  |  |  |  |
| PDIA6 | 1.13 |  |  |  |  |  | 0.72 | 0.05 | -0.67 | 0.44 | -0.28 | 0.39 |
| PFKL |  |  |  |  |  | 1.09 |  |  |  |  |  |  |
| PFN1 | 0.96 | 0.92 | 0.99 | 0.93 |  |  | -0.17 | 0.16 | 0.33 | 0.05 | 0.22 | -0.11 |
| PGD | 0.97 |  |  |  |  |  | 0.19 | -0.19 | -0.38 | 0.29 | 0.10 | 0.47 |
| PGK1 | 1.07 |  | 0.97 |  | 0.95 |  | -0.06 | 0.16 | 0.22 | -0.21 | -0.15 | -0.37 |
| PGLS | 0.82 |  | 1.16 |  | 1.15 |  | 0.05 | -0.60 | -0.64 | -0.27 | -0.32 | 0.32 |
| PGLYRP1 | 1.16 |  |  |  |  |  |  |  |  |  |  |  |
| PLIN3 |  |  | 0.91 |  |  |  | -0.24 | -0.56 | -0.33 | -0.41 | -0.17 | 0.15 |
| PLS1 |  |  | 0.90 |  |  |  |  |  |  |  |  |  |
| PPIA | 0.91 |  | 1.13 |  |  |  | -0.16 | 0.20 | 0.36 | -0.20 | -0.04 | -0.40 |
| PPIB | 0.74 |  | 1.50 |  | 1.42 | 0.94 | 0.05 | -0.26 | -0.31 | -0.55 | -0.60 | -0.29 |
| PPP2R1A |  |  |  |  |  | 0.93 |  |  |  |  |  |  |
| PRDX3 |  |  |  |  |  | 0.92 | 0.00 | -0.19 | -0.20 | 0.18 | 0.18 | 0.38 |
| PRDX5 | 0.94 |  |  |  |  |  | -0.21 | -0.35 | -0.14 | -0.30 | -0.09 | 0.05 |
| PREP | 0.89 |  |  |  |  |  | -0.25 |  |  | 0.67 | 0.92 |  |
| PRG3 | 0.85 | 0.72 | 0.85 | 1.11 | 1.32 | 1.55 |  |  |  |  |  |  |
| PRKAR1A |  |  |  |  |  | 0.91 | 0.38 | 0.62 | 0.24 | 0.31 | -0.08 | -0.32 |
| PRTN3 | 0.93 | 1.18 | 1.23 | 1.12 | 1.16 | 0.96 |  |  |  |  |  |  |
| PSMA5 |  | 0.93 | 0.90 |  |  | 1.10 | 0.56 | -0.13 | -0.70 | -0.01 | -0.57 | 0.12 |
| PSMA7 |  |  |  |  |  | 0.94 | -0.08 | -0.04 | 0.04 | -0.03 | 0.05 | 0.01 |
| PSMC5 |  |  |  |  | 0.90 |  | 0.09 | -0.28 | -0.37 | -0.18 | -0.27 | 0.10 |
| PSME2 |  |  | 1.23 |  | 1.18 |  | -0.37 | 0.70 | 1.06 | 0.67 | 1.03 | -0.03 |
| PTBP1 |  |  |  |  |  | 1.22 | 0.27 | -0.29 | -0.55 | -0.31 | -0.58 | -0.03 |
| PTMAP7 |  | 0.87 | 0.88 |  |  | 1.12 |  |  |  |  |  |  |
| PTPN14 | 0.79 |  |  |  |  |  |  |  |  |  |  |  |
| PTPN6 |  |  | 0.95 | 0.93 | 0.91 |  | -0.53 | 0.35 | 0.88 | 0.44 | 0.97 | 0.10 |
| PYGB |  |  |  |  |  | 0.94 | -0.14 | 0.03 | 0.16 | 0.12 | 0.25 | 0.09 |
| PYGL | 0.92 |  |  | 0.84 | 0.92 | 0.88 | -0.03 | 0.19 | 0.22 | 0.54 | 0.57 | 0.36 |
| QSOX1 |  |  |  |  | 1.17 |  | -0.08 | -0.13 | -0.05 | 0.18 | 0.26 | 0.30 |
| RAB10 | 0.89 |  |  |  |  |  | 0.81 | 0.67 | -0.15 | 0.92 | 0.64 | 0.26 |
| RAB11B | 0.85 |  |  | 0.88 |  |  |  |  |  |  |  |  |
| RAB31 |  |  |  |  |  | 0.93 | 0.09 | 0.85 | 0.76 | 0.11 | 0.01 | -0.75 |
| RAB32 | 1.12 |  |  |  |  |  | -0.30 |  |  | -0.07 | 0.22 |  |
| RAB35 | 0.93 |  |  |  |  | 0.95 |  |  |  |  |  |  |
| RAB7A | 0.93 |  |  |  |  |  | 0.23 | -0.11 | -0.34 | 0.11 | -0.11 | 0.23 |
| RAB8A | 0.90 |  |  |  |  |  | 0.26 | -0.03 | -0.29 | 0.92 | 0.66 | 0.94 |
| RAC2 | 1.33 |  |  |  | 0.74 | 0.85 | 0.21 | 0.46 | 0.24 | 0.07 | -0.14 | -0.38 |
| RAN |  |  | 0.89 |  | 0.92 |  | 0.27 | -0.47 | -0.74 | -0.28 | -0.56 | 0.18 |
| RAP2B | 0.80 |  |  |  |  |  | 0.61 | 0.50 | -0.11 | 0.51 | -0.10 | 0.01 |
| REEP5 |  |  |  | 0.74 |  |  | -0.21 | 0.83 | 1.03 | -0.13 | 0.07 | -0.96 |
| RETN | 1.14 | 1.24 | 1.10 | 1.22 | 1.07 |  |  |  |  |  |  |  |
| RHOA | 0.95 | 0.95 |  | 0.94 |  |  | 0.15 | -0.18 | -0.33 | -0.40 | -0.55 | -0.45 |
| RHOG | 1.19 |  |  |  | 0.85 | 0.88 | 0.61 | 0.28 | -0.33 | -0.28 | -0.89 | -0.56 |
| RNASE2 | 1.14 | 0.95 | 0.85 |  | 0.96 | 1.15 |  |  |  |  |  |  |
| RNASE3 | 0.85 | 0.75 | 0.85 | 0.92 |  | 1.26 |  |  |  |  |  |  |
| RNPEP | 0.93 | 0.93 |  |  |  |  |  |  |  | 0.26 |  |  |
| RP2 | 0.80 |  |  |  |  |  | 0.36 |  |  | -0.33 | -0.69 |  |
| RPS27A |  |  |  | 0.93 |  | 0.93 | 0.43 | 0.27 | -0.16 | 0.02 | -0.41 | -0.25 |
| RTN3 | 1.27 |  |  |  |  |  | 0.42 | -0.23 | -0.65 | -0.20 | -0.62 | 0.03 |
| S100A11 |  |  |  |  |  | 1.12 | -0.23 | 0.76 | 0.99 | 0.04 | 0.27 | -0.72 |
| S100A12 |  | 0.93 | 0.94 | 0.90 | 0.91 | 0.97 | 0.68 |  |  | -0.82 | -1.50 |  |
| S100A4 | 0.77 | 0.80 |  | 0.84 |  | 1.06 | -0.15 | 1.06 | 1.05 | 0.17 | 0.33 | -0.72 |
| S100A6 |  | 0.90 |  |  |  |  | -0.37 | 0.88 | 1.26 | -0.02 | 0.36 | -0.90 |
| S100A8 | 1.05 | 1.03 |  | 1.05 |  | 1.02 | 0.40 | -0.29 | -0.69 | 0.49 | 0.10 | 0.79 |
| S100A9 | 0.93 | 0.96 | 1.06 |  | 1.13 | 1.09 | -0.16 | 0.55 | 0.71 | 0.03 | 0.19 | -0.52 |
| SARS |  |  | 0.81 |  |  |  |  |  |  |  |  |  |
| SDCBP | 1.29 |  |  |  |  |  | 0.78 | 0.34 | -0.44 | 0.66 | -0.12 | 0.32 |
| SEC22B |  |  |  |  |  | 0.98 | -0.26 |  |  |  |  |  |
| SEPT2 |  |  |  |  |  | 1.11 | 0.37 | 0.16 | -0.22 | -0.39 | -0.76 | -0.55 |
| SERPINB1 |  |  |  |  | 0.92 | 0.90 | 0.41 | -0.30 | -0.71 | 0.75 | 0.34 | 1.05 |
| SF3B2 |  |  |  |  |  | 1.18 | 0.26 | 0.24 | -0.02 | 0.62 | 0.36 | 0.38 |
| SFPQ |  |  |  |  |  | 0.89 | 0.21 | -1.03 | -1.24 | -0.14 | -0.35 | 0.89 |
| SH3BGRL | 0.89 |  |  |  |  |  | 0.40 | 0.13 | -0.27 | 0.28 | -0.12 | 0.15 |
| SIGLEC14 | 1.13 |  |  |  |  |  |  |  |  |  |  |  |
| SLC2A3 |  |  | 1.09 |  |  | 0.95 | -0.18 | -0.47 | -0.29 | 0.04 | 0.22 | 0.50 |
| SLC4A1 | 0.67 |  | 1.41 |  | 1.31 |  |  |  |  |  |  |  |
| SLC9A3R1 |  | 1.31 |  |  |  |  | -0.24 | 0.04 | 0.28 | 0.07 | 0.31 | 0.03 |
| SND1 | 1.19 |  |  |  |  |  |  |  |  |  |  |  |
| SNRPF |  | 0.70 |  |  |  |  | 0.11 | -0.10 | -0.21 | -0.16 | -0.27 | -0.06 |
| SOD1 | 0.91 |  | 1.09 |  |  | 0.97 | 0.64 | -0.37 | -1.01 | 0.00 | -0.64 | 0.37 |
| SPC18 |  |  | 0.92 |  |  |  |  |  |  |  |  |  |
| SPCS2 |  |  |  |  |  | 0.92 | 0.68 | 0.20 | -0.48 | 0.49 | -0.19 | 0.29 |
| STK4 |  |  |  |  |  | 0.90 |  |  |  |  |  |  |
| STMN1 |  |  |  |  |  | 0.94 | 0.74 | -1.84 | -2.58 | -0.29 | -1.03 | 1.56 |
| STT3B | 1.11 |  |  |  |  |  |  | -0.33 |  | -1.06 |  | -0.73 |
| SULT1A1 | 0.85 |  |  |  |  |  | -0.11 | 0.22 | 0.33 | -0.50 | -0.40 | -0.73 |
| SURF4 |  |  |  |  |  | 0.95 | 0.44 | -0.81 | -1.25 | 0.19 | -0.25 | 1.00 |
| SYK | 1.46 |  | 0.73 |  |  |  | 0.12 |  |  |  |  |  |
| TALDO1 | 0.89 | 0.90 | 1.02 | 0.91 |  |  | 0.19 | -0.07 | -0.26 | 0.03 | -0.16 | 0.10 |
| TES |  |  |  |  |  | 0.96 |  |  |  |  |  |  |
| TF | 1.16 | 1.22 |  |  |  |  | -0.05 | -0.26 | -0.20 | -0.32 | -0.26 | -0.06 |
| TKTL1 |  | 0.84 | 0.83 | 0.93 | 0.92 | 1.13 |  |  |  |  |  |  |
| TLN1 | 0.98 | 0.90 | 0.93 |  | 1.06 | 1.06 |  |  |  |  |  |  |
| TOLLIP |  |  |  |  |  | 1.13 |  |  |  |  |  |  |
| TPI1 | 0.96 | 0.97 |  | 0.97 |  |  | 0.29 | -0.10 | -0.40 | 0.13 | -0.17 | 0.23 |
| TPM1 |  |  | 0.78 | 0.75 | 0.73 |  | -0.40 | -0.09 | 0.32 | -0.68 | -0.28 | -0.60 |
| TPM3 |  |  | 0.90 | 0.89 | 0.87 | 0.96 | 0.37 |  | 0.20 | 0.17 | -0.20 |  |
| TPT1 |  |  |  |  |  | 0.90 | 0.18 | 0.57 | 0.48 | 0.09 | -0.09 | -0.40 |
| TRA1 | 1.18 |  | 0.89 | 1.16 |  | 1.14 |  | 0.66 |  |  |  | -0.57 |
| TSN |  |  |  |  |  | 0.91 | -0.01 |  | 0.09 | 0.15 | 0.16 |  |
| TWF2 |  |  |  |  |  | 0.91 | 0.31 | 0.07 | -0.37 | 0.25 | -0.06 | 0.07 |
| TXN | 0.87 |  |  |  |  | 0.97 | 0.46 | -0.06 | -0.55 | 0.16 | -0.31 | 0.31 |
| TYMP | 0.87 |  |  | 0.89 |  | 0.96 | 0.90 | -0.09 | -0.44 | 0.47 | -0.44 | 0.24 |
| UBE2L3 | 1.56 |  |  |  |  |  | 0.12 | 0.46 | 0.07 | -0.08 | -0.20 | 0.00 |
| UCHL1 | 1.10 |  |  |  |  |  |  | 0.19 |  |  |  | -0.28 |
| UGP2 | 1.18 |  | 0.82 |  | 0.85 |  | 0.36 |  | -0.26 | 0.18 | -0.18 | 0.08 |
| VAMP8 |  |  |  | 1.56 |  |  | 0.19 | 0.10 | -0.50 | -0.08 | -0.27 | -0.26 |
| VAPA | 0.82 |  |  |  |  |  |  | -0.31 |  | -0.59 |  | 0.23 |
| VCL | 0.94 |  | 1.07 |  |  | 0.98 | -1.03 | -0.17 | 0.49 | -1.03 | -0.01 | -0.42 |
| VCP |  | 1.10 | 1.17 |  | 1.14 |  |  | -0.54 |  | -1.64 |  | -0.49 |
| VPS28 |  |  |  |  |  | 0.87 | -0.21 | -0.67 | 0.01 | -0.24 | -0.03 | -0.97 |
| VSTM1 |  |  |  |  |  | 0.98 |  | -0.20 |  |  |  | -0.04 |
| WAS | 1.16 |  |  |  |  |  |  |  |  |  |  |  |
| WIPF1 |  |  |  |  |  | 0.86 | 0.02 |  | 0.61 | 0.6 | 0.58 |  |
| YWHAB |  |  |  | 0.89 | 0.91 | 0.90 | 0.30 | 1.03 | 0.73 | 0.22 | -0.08 | -0.81 |
| YWHAZ |  |  |  |  |  | 0.95 | -0.03 | -1.10 | -1.07 | -0.41 | -0.38 | 0.69 |

The negative values in microarray represent the higher value for denominator, while positive values represent the higher value for numerator in examined ratio of MPNs
