# Supplementary material for: Microarray and Proteomic Analyses of Myeloproliferative Neoplasms with a Highlight on the mTOR Signaling Pathway
Source: PLoS One. 2015 Aug 14;10(8):e0135463. doi: 10.1371/journal.pone.0135463 (PMC4537205; doi:10.1371/journal.pone.0135463)
Supplement: S6 Table — (DOCX) [file pone.0135463.s006.docx]

**S6 Table.** Gene expression previously determined by proteomic studies in CD34^+^ cells of MPNs, analyzed my microarray.

| **CD34^+^ cells** | **ET** | | **PV** | | **PMF** | | **Mut0** | |
| --- | --- | --- | --- | --- | --- | --- | --- | --- |
| Genes | **Mean** | **SD** | **Mean** | **SD** | **Mean** | **SD** | **Mean** | **SD** |
| ACAT2 |  |  | -0.95 | 0.20 |  |  | -0.76 | 0.00 |
| ACSL1 | 2.8 | 0.70 | 3.39 | 0.40 | 3.84 | 0.00 | 2.52 | 0.80 |
| ACTG | -0.64 | 0.80 | -0.2 | 0.80 | -0.21 | 0.70 | 0.17 | 0.40 |
| ACTN1 | -0.97 | 0.70 | -1.09 | 0.40 | -0.73 | 0.30 | -1.09 | 1.00 |
| ACTR2 | 1.07 | 0.50 | 1.68 | 0.50 | 1.3 | 0.10 | 1.24 | 0.30 |
| ACTR3 | 0.13 | 0.10 | 0.74 | 0.30 | 0.14 | 0.10 | -0.03 | 0.30 |
| AKR1B1 | -1.11 | 0.20 | -1.13 | 0.20 | 0.03 | 0.00 | -1.16 | 0.40 |
| ALB | -4.12 | 0.10 | -4.53 | 0.10 | -4.14 | 0.00 | -4.23 | 0.20 |
| ALDOA | 1.8 | 0.50 | 1.72 | 0.30 | 1.94 | 0.50 | 1.55 | 0.20 |
| ALOX5AP | 1.42 | 0.00 | 0.82 | 0.00 |  |  |  |  |
| ANXA1 | 1.62 | 1.10 | 2.3 | 0.40 | 1.6 | 0.50 | 1.55 | 0.70 |
| ANXA2 | -1.33 | 1.00 | -0.79 | 0.30 | -2.16 | 0.80 | -1.39 | 0.70 |
| ANXA4 | 0.95 | 0.40 | 1.37 | 0.30 | 1.05 | 0.40 | 1.11 | 0.30 |
| ANXA5 | -0.08 | 0.20 | -0.31 | 0.60 | -1.2 | 0.20 | -0.58 | 0.40 |
| ANXA6 | 1.94 | 0.00 | 1.08 | 0.20 |  |  | 1.12 | 0.20 |
| APEX1 | 1.17 | 0.40 | 2.25 | 0.50 | 2.58 | 0.80 | 1.78 | 0.40 |
| ARHGDIB | 3.13 | 0.60 | 3.02 | 0.20 | 3.06 | 0.20 | 2.73 | 0.50 |
| ARPC1B | 1.94 | 0.40 | 2.18 | 0.30 | 2.1 | 0.60 | 2.41 | 0.30 |
| ARPC2 | 1.19 | 0.30 | 1.25 | 0.30 | 0.92 | 0.10 | 1.34 | 0.50 |
| ARPC3 | 0.88 | 0.50 | 1.05 | 0.30 | 0.38 | 0.10 | 0.92 | 0.40 |
| ARPC5 | 2.42 | 0.40 | 2.8 | 0.30 | 2.19 | 0.10 | 2.32 | 0.40 |
| ATP5A1 | 0.95 | 0.10 | 1.47 | 0.40 | 1.61 | 1.00 | 1.37 | 0.80 |
| ATP5B | -0.52 | 0.30 | -0.01 | 0.30 | 0.09 | 0.70 | -0.08 | 0.50 |
| ATP6V1E1 | 0.59 | 0.10 | 0.37 | 0.10 | 0.66 | 0.00 | 0.13 | 0.20 |
| BLVRB | -0.01 | 0.10 | -0.14 | 0.40 | -0.29 | 0.00 | 0.07 | 0.00 |
| BST1 | 1.17 | 0.00 | 2.34 | 0.00 | 2.39 | 0.00 | 1.16 | 0.00 |
| CA1 |  |  | 1.23 | 0.90 | -1.59 | 0.10 |  |  |
| CA2 | 3.69 | 0.00 | 2.2 | 0.20 |  |  |  |  |
| CAB39L | 0.93 | 0.70 | 0.92 | 0.70 | 0.69 | 0.00 | 0.37 | 0.20 |
| CALM2 | 0.17 | 0.70 | 0.58 | 0.30 | 0.02 | 0.50 | 0.35 | 0.40 |
| CALR |  |  |  |  | -1.2 | 0.00 | -0.79 | 0.00 |
| CAMP |  |  | 3.28 | 0.10 |  |  |  |  |
| CAND1 | 0.72 | 0.30 | 0.86 | 0.30 | 1.59 | 0.00 | 0.43 | 0.20 |
| CANX | 0.09 | 0.90 | 1.02 | 0.40 | 0.61 | 0.70 | 0.23 | 0.60 |
| CAP1 | 1.24 | 0.40 | 0.83 | 0.40 | 0.92 | 0.10 | 1.18 | 0.30 |
| CAPG | 1.76 | 0.20 | 2.22 | 0.20 |  |  | 1.24 | 0.00 |
| CAPZA1 | 0.56 | 1.00 | 0.62 | 0.70 | 1.44 | 0.40 | 0.38 | 0.60 |
| CAPZA2 | 2.52 | 0.40 | 2.6 | 0.60 | 2.93 | 0.30 | 2.46 | 0.20 |
| CAT | 2.19 | 0.40 | 2.95 | 0.60 | 1.9 | 0.00 | 2.98 | 0.60 |
| CBX3 | -0.03 | 0.50 | 0.7 | 0.40 | 0.64 | 0.60 | 0.06 | 0.50 |
| CCT2 | -0.28 | 0.50 | 0.8 | 0.40 | 1.38 | 1.00 | 0.68 | 0.90 |
| CCT3 | -1.19 | 0.10 | -0.84 | 0.30 | -0.04 | 0.00 | -1.26 | 0.80 |
| CCT5 | -1.8 | 0.00 | -1.77 | 0.20 | -1.29 | 0.00 | -2.22 | 0.00 |
| CD44 | 0.66 | 0.70 | 0.39 | 0.70 | 0.05 | 0.10 | 0.78 | 0.50 |
| CD59 |  |  | -0.15 | 0.50 | 0.75 | 0.60 | 0.47 | 0.50 |
| CFL1 | -0.29 | 0.60 | -0.16 | 0.40 | -0.36 | 0.30 | -0.04 | 0.60 |
| CHI3L1 | 1.39 | 0.00 |  |  | 1.07 | 0.00 |  |  |
| CHIT1 | -0.92 | 0.80 | -1.28 | 0.40 | -0.18 | 0.80 | -0.92 | 0.90 |
| CHMP5 | 1.67 | 0.50 | 2.09 | 0.40 | 1.73 | 0.20 | 1.44 | 0.40 |
| CLIC1 | 0.72 | 0.30 | 0.86 | 0.40 | 0.98 | 0.60 | 0.79 | 0.40 |
| CLTC | -0.89 | 0.30 | -0.41 | 0.50 | 0.07 | 0.00 | -0.69 | 0.10 |
| CORO1A | 4.02 | 0.50 | 4.15 | 0.40 | 3.57 | 0.10 | 4.01 | 0.90 |
| COTL1 | 1.6 | 0.30 | 1.41 | 0.60 | 0.7 | 0.00 | 1.88 | 0.10 |
| CPNE3 | 1.42 | 0.20 | 1.01 | 0.20 | 2.21 | 0.30 | 2.26 | 0.00 |
| CSK | 2.63 | 0.70 | 2.71 | 0.40 | 2.1 | 0.40 | 2.48 | 0.60 |
| CTSD | 0.33 | 0.40 | 0.17 | 0.30 | 0.09 | 0.20 | 0.46 | 0.40 |
| CTSS | 5.36 | 0.80 | 5.7 | 0.50 | 4.89 | 1.20 | 5.28 | 0.50 |
| DDT | -1.12 | 0.40 | -0.51 | 0.30 | -0.81 | 0.50 | -0.44 | 0.20 |
| DEK | 0.5 | 0.50 | 1.04 | 0.60 | 1.26 | 0.90 | 0.59 | 0.70 |
| DLST |  |  | 1.1 | 0.00 |  |  | 0.73 | 0.00 |
| DPP3 | -0.03 | 0.60 | -0.08 | 0.10 | 0.55 | 0.00 | -0.09 | 0.40 |
| DYNC1H1 | 0.01 | 0.40 | 0.18 | 0.60 | 0.04 | 0.00 | -0.46 | 0.20 |
| EEF1D | 1.05 | 0.50 | 0.74 | 0.40 | 1.03 | 0.90 | 1.09 | 0.90 |
| EFHD2 | 0.97 | 0.00 | 1.29 | 0.50 |  |  | 1.02 | 0.50 |
| EHD1 |  |  | 1.99 | 0.10 | 0.94 | 0.00 |  |  |
| EIF2S1 |  |  | 0.53 | 0.00 |  |  | 0.48 | 0.00 |
| EML4 | -0.17 | 0.40 | 0.37 | 0.10 |  |  | -0.14 | 0.00 |
| ENO1 | -2.1 | 0.30 | -1.95 | 0.40 | -1.16 | 0.90 | -1.53 | 0.40 |
| EVI2B | 2.19 | 0.70 | 2.4 | 0.80 | 1.84 | 1.00 | 1.31 | 1.30 |
| FAM129A |  |  | 0.83 | 0.00 | 2.1 | 0.00 | 1.58 | 0.00 |
| FBP1 | 1.14 | 0.40 | 1.14 | 0.10 |  |  | 1.61 | 0.10 |
| FCN1 | 4.45 | 0.50 | 4.04 | 0.30 | 1.57 | 1.60 | 3.54 | 0.90 |
| FLNA | -0.35 | 0.40 | -0.74 | 0.30 | -0.3 | 0.00 | 0.36 | 0.40 |
| FNBP1 | 2.72 | 0.30 | 2.39 | 0.30 | 2.08 | 0.70 | 3.19 | 0.10 |
| FOLR3 | 1.92 | 0.00 | 4.82 | 0.00 |  |  | 4.66 | 0.00 |
| G6PD | -0.35 | 0.00 |  |  | -0.98 | 0.00 |  |  |
| GAPDH | -2.55 | 0.30 | -2.39 | 0.40 | -2 | 0.50 | -1.97 | 0.40 |
| GDI2 | 0.81 | 0.40 | 1.37 | 0.50 | 1.54 | 0.60 | 0.81 | 0.20 |
| GLRX | 2.17 | 0.50 | 2.51 | 0.50 | 1.42 | 0.60 | 1.47 | 0.40 |
| GMFG | 3.58 | 0.90 | 3.67 | 0.50 | 3.79 | 0.10 | 3.79 | 0.50 |
| GNA13 | 1.84 | 0.00 | 2.09 | 0.30 | 1.61 | 0.00 | 1.87 | 0.00 |
| GNAI2 | 1.83 | 0.70 | 1.16 | 0.60 | 1.18 | 0.30 | 2.15 | 0.20 |
| GPI | -1.67 | 0.30 | -1.59 | 0.20 | -1.09 | 0.40 | -1.08 | 0.30 |
| GSR | -0.59 | 0.00 | -0.24 | 0.30 |  |  | -0.02 | 0.50 |
| GSTO1 | 0.05 | 0.60 | -0.07 | 0.30 | 0.2 | 0.10 | 0.11 | 0.50 |
| GSTP1 | -1.87 | 0.30 | -2.02 | 0.30 | -1.66 | 0.30 | -1.46 | 0.40 |
| GYG1 | -0.19 | 0.00 | 0.16 | 0.20 |  |  | 0.57 | 0.40 |
| H2AFY | 0.52 | 0.20 | 0.75 | 0.50 | 1.47 | 0.80 | 1.22 | 0.60 |
| HBB | 5.77 | 1.50 | 6.98 | 0.90 | 6.52 | 0.00 | 6.45 | 0.20 |
| HBD | -0.54 | 2.20 | 2.49 | 2.50 | -1.33 | 0.80 | -0.65 | 0.80 |
| HIST1H2BO | -0.46 | 0.20 | -1.06 | 0.30 | -0.59 | 1.00 | -0.71 | 0.80 |
| HK3 | 2.64 | 0.80 | 3.59 | 0.00 |  |  | 2.29 | 0.00 |
| HNRNPD | -0.95 | 0.50 | -0.53 | 0.30 | -0.61 | 0.30 | -0.77 | 0.20 |
| HP | -0.18 | 0.40 | 0.09 | 0.20 | -0.21 | 0.30 | 0.08 | 0.80 |
| HSP90AA1 | -2.12 | 0.70 | -1.62 | 0.80 | -1.18 | 0.50 | -1.93 | 0.70 |
| HSPA8 | -2.15 | 0.70 | -1.4 | 0.10 | -1.67 | 0.30 | -1.89 | 1.00 |
| HSP90AB1 | -1.62 | 0.50 | -0.83 | 0.60 | -0.42 | 0.80 | -1.22 | 0.50 |
| HSPA1A | 0.3 | 0.60 | 0.96 | 1.00 | 0.3 | 0.00 | -0.21 | 0.70 |
| ICAM3 | 2.18 | 0.60 | 2.34 | 0.30 | 2.65 | 0.80 | 2.69 | 0.30 |
| IFI30 | 1.41 | 0.90 | 1.53 | 0.80 | 1.12 | 0.00 | 1.26 | 1.10 |
| IFI35 | 0.98 | 0.00 | 1.17 | 0.50 | 0.68 | 0.00 | 0.93 | 0.00 |
| IQGAP1 | 1.66 | 0.50 | 1.78 | 0.70 | 0.71 | 0.50 | 1.55 | 0.60 |
| ITGA2B | -0.03 | 0.70 | -0.7 | 0.90 | -0.34 | 0.00 | 0.83 | 1.10 |
| ITGAM |  |  | 2.83 | 0.40 |  |  |  |  |
| KPNB1 | -1.97 | 0.30 | -1.62 | 0.30 | -1.38 | 0.00 | -1.72 | 0.40 |
| KRT10 | -1.16 | 0.20 | -0.88 | 0.30 | -0.81 | 0.00 | -0.59 | 0.20 |
| LCP1 | 4.33 | 0.60 | 4.56 | 0.60 | 4.22 | 0.30 | 4.26 | 0.60 |
| LDHB | -2.27 | 1.00 | -1.32 | 1.20 | -0.59 | 0.90 | -1.1 | 0.80 |
| LGALS1 | -1.09 | 0.30 | -1.42 | 0.30 | -2.42 | 0.40 | -0.7 | 0.60 |
| LTA4H | 2.01 | 0.50 | 2.57 | 0.50 | 2 | 0.00 | 1.76 | 0.30 |
| LXN | 0.77 | 0.10 | 1.17 | 0.80 | 1.62 | 0.50 | 0.62 | 0.30 |
| LYZ | 3.16 | 2.30 | 3.39 | 1.80 | 1.35 | 1.00 | 3.31 | 1.70 |
| MARCKS |  |  | -0.42 | 0.30 |  |  | -0.37 | 0.00 |
| MDH1 | -0.57 | 0.00 | -0.65 | 0.30 | -0.76 | 0.40 | -0.64 | 0.70 |
| ME2 | 1.28 | 0.40 | 1.63 | 0.40 | 1.09 | 0.10 | 0.56 | 0.30 |
| MIF | -0.82 | 0.40 | -0.65 | 0.80 | 0.09 | 0.70 | 0 | 0.50 |
| MNDA | 3.14 | 0.60 | 4.23 | 0.40 |  |  |  |  |
| MPO |  |  | 2.72 | 0.20 | 5.68 | 0.00 |  |  |
| MSN | -3.49 | 0.40 | -4.24 | 0.20 | -4.12 | 0.90 | -3.88 | 0.70 |
| MVP | 1.31 | 0.30 | 1.13 | 0.40 | 0.31 | 0.40 | 0.97 | 0.70 |
| MYL6 | 1.46 | 0.70 | 0.95 | 0.40 | 0.44 | 0.40 | 1.44 | 1.00 |
| MYO1F | 1.7 | 0.00 | 1.78 | 0.20 |  |  | 1.55 | 0.00 |
| NAMPT | 1.1 | 0.70 | 1.08 | 0.40 | 0.94 | 0.40 | 0.92 | 0.50 |
| NDRG1 | -0.21 | 0.10 | -0.61 | 0.10 | -0.53 | 0.00 | -0.47 | 0.20 |
| NME1 | -2.39 | 0.50 | -2.16 | 0.60 | -1.27 | 0.60 | -2.04 | 0.60 |
| NPC2 | 1.59 | 0.40 | 1.62 | 0.60 | 0.96 | 0.10 | 1.36 | 0.80 |
| NQO2 | 0.35 | 0.00 | -0.27 | 0.00 |  |  |  |  |
| NSF | 0.82 | 0.30 | 0.77 | 0.30 | 0.21 | 0.30 | 0.44 | 0.50 |
| ORM1 | -3.07 | 0.00 | -2.58 | 0.00 |  |  | -2.26 | 0.00 |
| OSTF1 | 1.67 | 0.00 | 1.52 | 0.30 | 1.16 | 0.00 | 1.48 | 0.00 |
| P4HB | -0.88 | 0.50 | -0.68 | 0.70 | -0.47 | 0.50 | -0.44 | 0.50 |
| PA2G4 | -0.1 | 0.50 | 0.04 | 0.40 | 0.58 | 0.70 | 0.83 | 0.60 |
| PAICS |  |  | -0.64 | 0.60 | 0.52 | 0.00 | -0.46 | 1.00 |
| PARK7 | 0.1 | 0.30 | 0 | 0.30 | 0.22 | 0.50 | 0.22 | 0.20 |
| PCBP1 | -0.57 | 0.60 | -0.2 | 0.60 | -0.65 | 0.30 | -0.63 | 0.20 |
| PDIA4 |  |  | -2.37 | 0.00 | -2.79 | 0.00 |  |  |
| PDIA6 | -0.8 | 0.60 | -0.08 | 0.40 | -0.13 | 0.90 | -0.52 | 0.50 |
| PFN1 | -1.33 | 0.40 | -1.5 | 0.30 | -1.66 | 0.20 | -1.55 | 0.40 |
| PGD | 1.36 | 0.40 | 1.55 | 0.40 | 1.73 | 0.10 | 1.26 | 0.60 |
| PGK1 | -0.99 | 0.30 | -1.05 | 0.40 | -1.21 | 0.50 | -0.84 | 0.30 |
| PGLS | 0.12 | 0.40 | 0.17 | 0.30 | 0.76 | 0.00 | 0.44 | 0.30 |
| PLIN3 | 0.12 | 0.30 | -0.11 | 0.40 | 0.45 | 0.50 | 0.3 | 0.50 |
| PPIA | 0.09 | 0.10 | -0.07 | 0.20 | -0.27 | 0.00 | 0.13 | 0.50 |
| PPIB | -2.36 | 0.40 | -2.32 | 0.20 | -2.06 | 0.00 | -1.76 | 0.20 |
| PRDX3 | 0.36 | 0.00 | 0.36 | 0.40 | 0.56 | 0.60 | 0.18 | 0.40 |
| PRDX5 | -0.74 | 0.30 | -0.95 | 0.30 | -0.6 | 0.30 | -0.65 | 0.30 |
| PREP | 2.01 | 0.00 | 1.76 | 0.20 |  |  | 1.09 | 0.00 |
| PRKAR1A | 0.25 | 0.40 | 0.63 | 0.40 | 0.01 | 0.30 | 0.32 | 0.30 |
| PSMA5 | -0.78 | 0.60 | -0.22 | 0.20 | -0.09 | 0.10 | -0.21 | 0.60 |
| PSMA7 | 0.19 | 0.40 | 0.11 | 0.40 | 0.15 | 0.80 | 0.14 | 0.60 |
| PSMC5 | -1.19 | 0.10 | -1.11 | 0.20 | -0.83 | 0.70 | -0.93 | 0.50 |
| PSME2 | -0.03 | 0.10 | -0.39 | 0.30 | -1.09 | 0.00 | -1.06 | 0.00 |
| PTBP1 | -0.5 | 0.40 | -0.24 | 0.50 | 0.05 | 0.80 | 0.07 | 0.40 |
| PTPN6 | 1.35 | 0.40 | 0.82 | 0.60 | 0.47 | 0.00 | 0.37 | 0.40 |
| PYGB | 0.13 | 0.00 | -0.01 | 0.20 | -0.03 | 0.00 | -0.12 | 0.00 |
| PYGL | 0.1 | 0.20 | 0.07 | 0.20 | -0.12 | 0.10 | -0.47 | 0.50 |
| QSOX1 | -0.6 | 0.40 | -0.68 | 0.50 | -0.55 | 0.20 | -0.86 | 0.20 |
| RAB10 | 1.57 | 0.70 | 2.38 | 0.50 | 1.71 | 0.50 | 1.46 | 0.30 |
| RAB31 | 1.73 | 0.40 | 1.83 | 0.40 | 0.97 | 0.00 | 1.72 | 0.50 |
| RAB32 | 1.02 | 0.00 | 0.72 | 0.00 |  |  | 0.79 | 0.00 |
| RAB7A | 0.07 | 0.40 | 0.3 | 0.20 | 0.41 | 0.00 | 0.18 | 0.20 |
| RAB8A | 0.17 | 0.10 | 0.43 | 0.20 | 0.46 | 0.20 | -0.49 | 0.10 |
| RAC2 | 3.22 | 0.50 | 3.43 | 0.30 | 2.97 | 0.30 | 3.36 | 0.50 |
| RAN | -1.56 | 0.30 | -1.29 | 0.30 | -0.82 | 0.70 | -1.01 | 0.60 |
| RAP2B | 1.25 | 0.30 | 1.86 | 0.20 | 1.36 | 0.00 | 1.35 | 0.70 |
| REEP5 | 1.59 | 0.30 | 1.38 | 0.40 | 0.56 | 0.00 | 1.51 | 0.20 |
| RHOA | 1.51 | 0.80 | 1.65 | 0.40 | 1.83 | 0.10 | 2.05 | 0.40 |
| RHOG | 1.64 | 1.00 | 2.26 | 0.30 | 1.97 | 0.00 | 2.53 | 0.30 |
| RP2 | 2.23 | 0.80 | 2.59 | 0.50 |  |  | 2.92 | 0.00 |
| RPS27A | 0.06 | 0.50 | 0.49 | 0.30 | 0.22 | 1.10 | 0.47 | 0.80 |
| RTN3 | 0.39 | 0.70 | 0.81 | 0.30 | 1.04 | 0.10 | 1.01 | 0.60 |
| S100A11 | -1.26 | 0.40 | -1.49 | 0.40 | -2.25 | 1.30 | -1.53 | 0.50 |
| S100A12 | 4 | 1.30 | 4.68 | 0.90 |  |  | 5.49 | 0.00 |
| S100A4 | 3.55 | 0.40 | 3.4 | 0.50 | 2.5 | 0.30 | 3.23 | 0.50 |
| S100A6 | 1.61 | 0.30 | 1.23 | 0.40 | 0.35 | 0.20 | 1.25 | 0.70 |
| S100A8 | 5.67 | 1.20 | 6.07 | 0.50 | 6.36 | 0.20 | 5.58 | 0.30 |
| S100A9 | 5.14 | 0.60 | 4.98 | 0.40 | 4.43 | 0.60 | 4.95 | 0.70 |
| SDCBP | 1.1 | 1.10 | 1.88 | 1.10 | 1.54 | 0.60 | 1.23 | 0.50 |
| SEC22B | 1.34 | 0.10 | 1.08 | 0.30 |  |  |  |  |
| SEPT2 | -0.08 | 0.70 | 0.29 | 0.60 | 0.14 | 0.30 | 0.68 | 0.50 |
| SERPINB1 | 2.39 | 0.60 | 2.8 | 0.60 | 3.1 | 0.00 | 2.05 | 0.00 |
| SF3B2 | -0.42 | 0.50 | -0.17 | 0.50 | -0.41 | 0.70 | -0.79 | 0.30 |
| SFPQ | 0.08 | 0.20 | 0.3 | 0.20 | 1.32 | 0.00 | 0.43 | 0.10 |
| SH3BGRL | 2.2 | 0.40 | 2.6 | 0.40 | 2.47 | 0.00 | 2.32 | 0.40 |
| SLC2A3 | 0.82 | 0.70 | 0.64 | 0.80 | 1.1 | 0.00 | 0.6 | 0.50 |
| SLC9A3R1 | 0.21 | 0.30 | -0.03 | 0.40 | -0.07 | 0.00 | -0.1 | 0.10 |
| SNRPF | -2.07 | 0.40 | -1.97 | 0.30 | -1.86 | 0.10 | -1.8 | 0.60 |
| SOD1 | -1.76 | 0.40 | -1.12 | 0.40 | -0.75 | 0.50 | -1.12 | 0.40 |
| SPCS2 | 0.62 | 0.60 | 1.3 | 0.40 | 1.1 | 0.40 | 0.81 | 0.40 |
| STMN1 | -1.22 | 0.30 | -0.48 | 0.50 | 1.37 | 0.00 | -0.19 | 0.50 |
| STT3B |  |  | 0.37 | 0.50 | 0.7 | 0.00 | 1.43 | 0.00 |
| SULT1A1 | 0.78 | 0.50 | 0.67 | 0.40 | 0.45 | 0.10 | 1.18 | 0.70 |
| SURF4 | -1.05 | 0.20 | -0.61 | 0.40 | 0.2 | 0.00 | -0.8 | 0.30 |
| SYK | 2.72 | 0.50 | 2.84 | 0.60 |  |  |  |  |
| TALDO1 | 1.94 | 0.30 | 2.14 | 0.40 | 2.21 | 0.30 | 2.11 | 0.50 |
| TF | -2.71 | 0.40 | -2.77 | 0.30 | -2.51 | 0.20 | -2.45 | 0.50 |
| TPI1 | -0.78 | 0.30 | -0.49 | 0.30 | -0.38 | 0.90 | -0.62 | 0.50 |
| TPM1 | -0.66 | 0.60 | -1.06 | 0.20 | -0.97 | 0.40 | -0.37 | 0.90 |
| TPM3 | 1.77 | 0.70 | 2.14 | 0.20 | 1.57 | 0.00 | 1.98 | 0.30 |
| TPT1 | 1.29 | 0.50 | 1.47 | 0.40 | 0.81 | 1.00 | 1.38 | 0.70 |
| TSN | 1 | 0.00 | 0.99 | 0.50 | 0.92 | 0.10 | 0.84 | 0.60 |
| TWF2 | 1.09 | 0.40 | 1.39 | 0.20 | 1.46 | 0.00 | 1.15 | 0.10 |
| TXN | -0.52 | 0.50 | -0.05 | 0.70 | 0.03 | 0.40 | -0.21 | 0.60 |
| TYMP | 3.16 | 0.80 | 4.06 | 0.20 | 3.6 | 0.00 | 3.6 | 0.40 |
| UBE2L3 | -0.84 | 0.40 | -0.72 | 0.40 | -0.91 | 0.40 | -0.64 | 0.10 |
| UGP2 | 0.95 | 0.50 | 1.31 | 0.50 | 1.21 | 0.20 | 1.13 | 0.40 |
| VAMP8 | 2.33 | 0.40 | 2.52 | 0.30 | 2.83 | 0.60 | 2.6 | 0.30 |
| VAPA |  |  | -0.8 | 0.10 | -0.63 | 0.10 | -0.2 | 0.00 |
| VCL | 2.37 | 1.10 | 1.34 | 0.80 | 1.88 | 0.00 | 2.37 | 1.10 |
| VCP |  |  | -1.71 | 0.00 | -1.04 | 0.00 | -0.07 | 0.00 |
| VPS28 | 0.63 | 0.00 | 0.43 | 0.40 | 0.63 | 0.00 | 0.66 | 0.30 |
| WIPF1 | 3.61 | 1.10 | 3.63 | 0.60 | 3 | 0.00 | 3.03 | 0.20 |
| YWHAB | -0.13 | 0.60 | 0.17 | 0.50 | -0.86 | 0.20 | -0.05 | 0.20 |
| YWHAZ | 1.98 | 0.50 | 1.95 | 0.70 | 3.05 | 0.50 | 2.36 | 0.50 |

The negative values represent downregulated genes, while positive values represent upregulated genes compared to HuURNA.
