# Supplementary material for: Microarray and Proteomic Analyses of Myeloproliferative Neoplasms with a Highlight on the mTOR Signaling Pathway
Source: PLoS One. 2015 Aug 14;10(8):e0135463. doi: 10.1371/journal.pone.0135463 (PMC4537205; doi:10.1371/journal.pone.0135463)
Supplement: S7 Table — (DOCX) [file pone.0135463.s007.docx]

**S7 Table.** Gene expression previously determined by proteomic studies in granulocytes of MPNs, analyzed my microarray.

| **Granulocyte** | **ET** | | **PV** | | **PMF** | | **Mut0** | |
| --- | --- | --- | --- | --- | --- | --- | --- | --- |
| Genes | **Mean** | **SD** | **Mean** | **SD** | **Mean** | **SD** | **Mean** | **SD** |
| ACTN1 |  | 0.00 | -2.03 | 0.00 | 0.72 | 0.00 | -1.45 | 0.00 |
| ACTN4 | 0.62 | 0.00 | -0.52 | 0.00 | -0.24 | 0.00 | -0.04 | 0.00 |
| ALB | -2.83 | 0.00 | -2.99 | 0.30 | -2.93 | 0.00 | -2.47 | 0.00 |
| ALDOA | 3.27 | 0.01 | 2.82 | 0.31 | 2.21 | 0.55 | 3.06 | 0.34 |
| ANXA2 | -1.36 | 0.13 | -2.14 | 0.35 | -2.53 | 0.53 | -1.78 | 0.60 |
| APEX1 |  |  | 2.79 | 0.00 | 1.21 | 0.00 |  |  |
| ARHGDIB | -1.13 | 0.00 | -0.21 | 0.58 | 1.44 | 2.42 | -1.19 | 0.00 |
| ARPC2 | -0.65 | 0.00 | -0.45 | 0.20 | 2.02 | 0.00 | -0.48 | 0.32 |
| ARPC3 | -1.45 | 0.00 | -1.61 | 0.23 | -0.75 | 1.26 | -1.55 | 0.42 |
| ATP5A1 | -0.71 | 0.31 | -0.70 | 0.18 | -0.43 | 0.89 | -0.75 | 0.18 |
| ATP5B |  |  | -2.88 | 0.00 | -0.56 | 0.00 | -1.85 | 0.00 |
| CALM2 | -2.07 | 0.00 | -2.03 | 0.32 | 0.00 | 0.00 | -1.70 | 0.00 |
| CANX | -0.99 | 0.00 | -1.17 | 0.00 | 0.08 | 0.00 |  |  |
| CAPZA1 |  |  | 2.35 | 0.00 | 1.54 | 0.00 | -0.91 | 0.00 |
| CBX3 | -0.63 | 0.00 | -1.10 | 0.11 | -0.52 | 0.71 | -1.36 | 0.65 |
| CCT5 |  |  | -1.37 | 0.00 | -2.39 | 0.00 |  |  |
| CFL1 | -1.41 | 0.11 | -1.53 | 0.50 | -0.59 | 1.13 | -2.04 | 0.63 |
| CHI3L1 |  |  | 1.82 | 0.00 | 2.81 | 0.00 |  |  |
| CHIT1 |  |  | -0.40 | 0.57 | -1.42 | 0.24 |  |  |
| CLIC1 |  |  | -2.04 | 0.00 | 1.72 | 0.00 |  |  |
| CLTC |  |  | -1.39 | 0.00 | -1.06 | 0.00 |  |  |
| CORO1A | 0.50 | 0.00 | 1.28 | 0.35 | 3.22 | 2.52 | 0.40 | 0.00 |
| CPNE3 | 1.47 | 0.00 | 3.61 | 0.00 | 1.57 | 0.00 | 2.57 | 0.00 |
| CSK |  |  |  |  | 3.34 | 0.00 |  |  |
| CTSD | 1.03 | 0.32 | 1.02 | 0.72 | 1.03 | 0.54 | 1.65 | 0.01 |
| CTSG |  |  | 3.24 | 0.00 | 6.42 | 0.00 |  |  |
| DDT |  |  | -1.06 | 0.00 | -0.84 | 0.00 |  |  |
| EEF1D | 2.56 | 0.13 | 2.52 | 0.34 | 1.78 | 0.61 | 2.50 | 0.63 |
| ENO1 | -2.16 | 0.27 | -2.10 | 0.30 | -2.14 | 0.17 | -2.30 | 0.52 |
| FAM189B | -0.45 | 0.00 | -0.39 | 0.00 | -0.54 | 0.95 | 0.55 | 0.00 |
| FCN1 | 0.44 | 0.00 | 0.57 | 0.00 | 2.98 | 2.08 | 0.75 | 0.20 |
| FNBP1 |  |  |  |  | 2.58 | 0.00 | 2.26 | 0.00 |
| GAPDH | -2.26 | 0.26 | -2.37 | 0.28 | -2.02 | 0.37 | -2.87 | 0.56 |
| GMFG |  |  | 0.98 | 0.00 | 3.04 | 0.00 |  |  |
| GNAI2 |  |  | -0.02 | 0.45 | 1.26 | 2.14 | -0.87 | 0.00 |
| GPI | -1.28 | 0.00 | -1.88 | 0.00 | -0.40 | 0.00 | -1.13 | 0.00 |
| GSR |  |  |  |  | 0.05 | 0.00 | -0.13 | 0.00 |
| GSTP1 | -1.25 | 0.08 | -1.44 | 0.82 | -1.90 | 0.50 | -1.23 | 0.33 |
| GYG1 |  |  | 0.56 | 0.00 | 0.51 | 0.00 |  |  |
| HBD | -2.33 | 0.03 | -1.74 | 0.30 | 0.83 | 3.01 | -1.86 | 0.12 |
| HIST1H2BO | 0.54 | 0.02 | 1.38 | 0.70 | 0.22 | 0.67 | 0.67 | 0.53 |
| HK3 |  |  |  |  | 4.27 | 0.00 | 1.72 | 0.00 |
| HP | 1.32 | 0.32 | 2.19 | 0.97 | 2.26 | 0.97 | 1.57 | 0.78 |
| HSP90AA1 | -2.19 | 0.00 | -2.71 | 0.00 | -0.39 | 0.00 | -1.57 | 0.00 |
| HSPA8 | -3.25 | 0.00 | -3.93 | 0.00 | -2.64 | 0.00 | -3.36 | 0.00 |
| HSP90AB1 | -1.64 | 0.00 | -1.35 | 0.13 | -1.78 | 0.04 | -1.69 | 0.42 |
| ICAM3 |  |  | 0.42 | 0.00 | 3.77 | 0.00 |  |  |
| ITGA2B |  |  |  |  | -1.21 | 0.00 | -0.97 | 0.00 |
| KPNB1 | -1.40 | 0.10 | -1.68 | 0.00 | -1.58 | 0.52 | -1.62 | 0.55 |
| KRT10 |  |  |  |  | -1.05 | 0.00 | -0.07 | 0.00 |
| LCP1 |  |  | 0.41 | 0.00 | 4.98 | 0.00 | -0.45 | 0.00 |
| LDHB | -2.81 | 0.00 | -2.45 | 0.00 | -1.60 | 0.00 | -2.58 | 0.28 |
| LGALS1 |  |  | -2.50 | 0.00 | -1.90 | 0.00 | -1.39 | 0.00 |
| LYZ | 0.47 | 0.00 | 0.20 | 0.00 | 4.84 | 0.00 | 0.56 | 0.00 |
| ME2 |  |  |  |  | 1.85 | 0.00 | 1.72 | 0.00 |
| MIF | -0.89 | 0.00 | -1.85 | 0.00 | -0.44 | 0.30 | -0.64 | 0.04 |
| MSN | -2.16 | 0.04 | -2.11 | 0.13 | -3.05 | 0.84 | -1.83 | 0.31 |
| MYL6 | -1.28 | 0.03 | -0.58 | 0.25 | 0.18 | 1.38 | -1.30 | 0.19 |
| NAMPT | 1.16 | 0.02 | 1.19 | 0.16 | 1.93 | 0.67 | 1.28 | 0.33 |
| NME1 | -1.14 | 0.14 | -1.42 | 0.33 | -1.81 | 0.32 | -1.41 | 0.27 |
| NPC2 |  |  | -0.93 | 0.00 | 0.08 | 0.56 | -0.40 | 0.00 |
| ORM1 |  |  | -1.81 | 0.00 | -0.40 | 0.00 | -0.86 | 0.00 |
| ORM2 |  |  | 0.06 | 0.00 | -0.52 | 0.00 |  |  |
| P4HB | -2.29 | 0.00 | -3.00 | 0.00 | -1.12 | 1.07 | -2.05 | 0.00 |
| PAICS |  |  | -0.74 | 0.00 | -1.45 | 0.00 |  |  |
| PCBP1 | -1.72 | 0.00 | -1.69 | 0.00 | -1.46 | 0.90 | -1.97 | 0.28 |
| PDIA4 | -1.38 | 0.00 | -1.48 | 0.00 | -2.56 | 0.00 | -1.34 | 0.00 |
| PDIA6 | -2.23 | 0.10 | -2.35 | 0.51 | -1.65 | 0.91 | -2.20 | 0.32 |
| PFN1 | -1.67 | 0.08 | -1.69 | 0.58 | -1.24 | 0.76 | -1.79 | 0.25 |
| PGK1 | -0.59 | 0.00 | -0.68 | 0.00 | -0.99 | 0.00 |  |  |
| PLIN3 | 0.21 | 0.24 | 0.09 | 0.00 | 0.01 | 0.46 | 0.05 | 0.39 |
| PPIA | -1.77 | 0.10 | -2.00 | 0.46 | -1.93 | 0.25 | -2.05 | 0.30 |
| PPIB |  |  | -2.52 | 0.00 | -2.54 | 0.00 |  |  |
| PRDX5 | -0.66 | 0.15 | -0.79 | 0.65 | -0.38 | 0.57 | -0.75 | 0.16 |
| PSMA5 | 0.23 | 0.00 | -0.47 | 0.00 | -0.11 | 0.02 | 0.05 | 0.27 |
| PSMA7 | -1.17 | 0.06 | -1.61 | 0.37 | -0.65 | 1.18 | -1.51 | 0.44 |
| PTBP1 |  |  | -1.78 | 0.00 | -0.65 | 0.00 |  |  |
| PTPN6 | 0.20 | 0.00 | -0.27 | 0.00 | -1.05 | 0.00 | 0.64 | 0.21 |
| QSOX1 | 0.11 | 0.00 | -0.52 | 0.00 | 0.16 | 0.19 | 0.11 | 0.00 |
| RAB35 | -2.12 | 0.00 |  |  |  |  | -1.94 | 0.00 |
| RAB7A | 1.30 | 0.00 | 0.49 | 0.00 | 0.40 | 0.13 | 2.12 | 0.00 |
| RAC2 | 0.92 | 0.00 |  |  | 3.18 | 1.62 | 1.40 | 0.21 |
| RPS27A | -2.38 | 0.21 | -2.38 | 0.23 | -1.70 | 1.12 | -2.78 | 0.51 |
| S100A11 | -1.10 | 0.16 | -1.02 | 0.08 | -0.53 | 0.73 | -1.08 | 0.40 |
| S100A4 | -0.94 | 0.19 | -0.63 | 0.73 | 0.99 | 1.52 | -0.82 | 0.02 |
| S100A6 | -1.43 | 0.19 | -1.24 | 0.17 | 0.87 | 2.54 | -1.27 | 0.27 |
| S100A9 | 2.04 | 0.33 | 5.10 | 0.20 | 5.64 | 1.53 | 2.52 | 1.06 |
| SDCBP |  |  |  |  | 3.34 | 0.00 | -1.19 | 0.00 |
| SEPT2 |  |  | 1.52 | 0.00 | -0.70 | 0.00 |  |  |
| SF3B2 | -0.98 | 0.01 | -1.47 | 0.00 | -1.04 | 0.61 | -1.31 | 0.49 |
| SFPQ |  |  | 0.65 | 0.00 | -0.02 | 0.00 |  |  |
| SH3BGRL | 0.82 | 0.27 | 0.22 | 0.20 | 1.22 | 0.68 | 0.97 | 0.39 |
| SNRPF | -2.06 | 0.24 | -2.09 | 0.23 | -2.48 | 0.09 | -2.08 | 0.23 |
| SOD1 | -1.60 | 0.00 | -1.93 | 0.50 | -1.87 | 0.16 | -1.59 | 0.59 |
| SPCS2 |  |  | -1.97 | 0.00 | 0.88 | 0.00 |  |  |
| SULT1A1 | 0.39 | 0.60 | 0.53 | 0.25 | -0.42 | 0.90 | 0.69 | 0.72 |
| TALDO1 | -0.96 | 0.00 | -0.38 | 0.17 | 1.07 | 2.10 | -1.08 | 0.03 |
| TF | -0.97 | 0.04 | -1.57 | 0.07 | -2.55 | 0.46 | -1.11 | 0.47 |
| TPI1 | -1.95 | 0.01 | -1.96 | 0.34 | -1.54 | 0.66 | -2.06 | 0.31 |
| TPM3 | -1.17 | 0.10 | -1.41 | 0.19 | 0.39 | 1.61 | -1.26 | 0.05 |
| TPT1 | -2.91 | 0.27 | -2.51 | 0.20 | -1.26 | 1.87 | -3.32 | 0.42 |
| TXN | -0.67 | 0.98 | -0.80 | 0.51 | -0.42 | 1.51 | -1.54 | 0.12 |
| UGP2 | 0.51 | 0.09 | 0.13 | 0.09 | -0.28 | 0.82 | 0.65 | 0.22 |
| VAMP8 |  |  | 0.38 | 0.00 | 1.77 | 0.60 | 1.35 | 0.00 |
| YWHAB |  |  | -1.28 | 0.00 | -0.14 | 0.00 |  |  |
| YWHAZ | 2.33 | 0.12 | 2.57 | 0.93 | 2.47 | 0.18 | 2.30 | 0.31 |

The negative values represent downregulated genes, while positive values represent upregulated genes compared to HuURNA.
